# Bioactive Ion‐Confined Ultracapacitive Memristors with Neuromorphic Functions

**DOI:** 10.1002/anie.202412674

**Published:** 2024-11-07

**Authors:** Panlong Li, Joanna Feder‐Kubis, Jonas Kunigkeit, Mariola Zielińska‐Błajet, Eike Brunner, Julia Grothe, Stefan Kaskel

**Affiliations:** ^1^ Inorganic Chemistry Center I Technische Universität Dresden Bergstrasse 66 01069 Dresden Germany; ^2^ Faculty of Chemistry Wrocław University of Science and Technology Wybrzeże Wyspiańskiego 27 Wrocław 50-370 Poland; ^3^ Bioanalytical Chemistry Technische Universität Dresden Bergstrasse 66 01069 Dresden Germany; ^4^ Fraunhofer IWS Winterbergstrasse 28 01277 Dresden Germany

**Keywords:** iontronics, supercapacitors, ionic memristor, nanoporous carbon, bioactive ionic liquids

## Abstract

The field of bioinspired iontronics, bridging electronic devices and ionic systems, has multiple biological applications. Carbon‐based ultracapacitive devices hold promise for controlling bioactive ions via electric double layers due to their high‐surface‐area and biocompatible porous carbon electrodes. However, the interplay between complex bioactive ions and porous carbons remains unclear due to the variety of structures of bioactive ions present in biological systems. Herein, we investigate the adsorption behavior of a series of bioactive ammonium‐based cations with varying alkyl chain lengths in nanoporous carbons. We find that strong physisorption results from the synergistic hydrophobic interaction and electrostatic attraction between porous carbons (with a negative zeta potential) and bioactive cations. Bioactive cations with varying alkyl chain lengths can be irreversibly physically adsorbed and confined within nanoporous carbons resulting in anion enrichment and depletion during electric polarization. This situation, in turn, results in a characteristic memristive behavior in all‐carbon capacitive ionic memristor devices. Our findings highlight the relationship between the resistance state of the memristor and ion adsorption mechanisms in all‐carbon capacitive devices, which hold potential for future transmitter delivery, biointerfacing, and neuromorphic devices.

## Graphical abstract

Pronounced physisorption of bioactive cations with a hydrophobic alkyl chain by porous carbons with a negative zeta potential results in confined cations, which triggers anion enrichment and depletion under electric polarization and enables rapid differential ionic memresistance behavior for cations with strong and weak interactions with porous carbons.

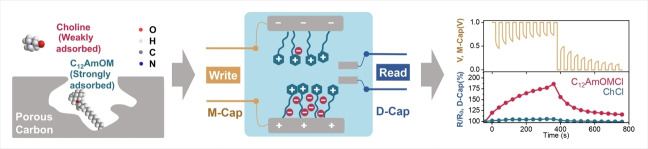

## Introduction

Iontronics, also termed ion‐controlled electronics, involves the modulation of electronic signals to change the behavior of ions. Accordingly, this field bridges electronics and ion‐based biological systems.[^1^, ^2^, ^3^] Iontronic devices, based on ionic charge carriers, have been widely explored as ionic diodes,[^4^, ^5^] ionic transistors,[^6^, ^7^] and ionic memristors.[^8^, ^9^] Electric double layer capacitors (EDLCs, also termed ultracapacitors) electrically adsorb/desorb ions on the surface of electrodes to balance and store opposite charges in a controllable manner.^[10]^ That enables operating ions to build advanced functional iontronic devices.^[11]^ Nanoporous carbons with higher specific surface areas (SSAs) than those of the metal electrodes widely used in iontronics, generate extra‐high unit area capacitances and low gate voltages.^[12]^ Furthermore, nanoporous carbons are compatible with biological systems,^[13]^ which in turn could enable ion manipulation in iontronics for additional biological applications such as drug delivery,[^14^, ^15^] toxins removal,^[16]^ bioelectronic devices in living matter,^[17]^ human‐machine interfacing,^[18]^ and neuromorphic computing.^[19]^

Recently, we proposed a series of EDLC‐based iontronic devices with functions of conventional diodes and transistors realized via electrically‐driven ion adsorption and desorption on the polarized nanoporous carbons.[^4^, ^20^] A 2‐terminal diode with a high rectification ratio was realized in a capacitive analog of a semiconductor‐based diode (CAPode) via the ion size‐sieving effect in two different carbons with small and large pore sizes.^[4]^ A 3‐terminal transistor with a high on‐off ratio was achieved in a switchable carbon‐based supercapacitor using a third gate electrode (G‐Cap).^[20]^ Finally, a 4‐terminal transistor with capacitance‐switching behavior was built based on bioactive choline cations; these choline cations carry specific chemical information that can regulate biological functions in the human body.^[11]^ However, these devices are limited as they assume only two distinct states (“on” and “off”) as required for binary logics. In contrast, neuromorphic devices mimic biological nerve systems based on the representation of information in multiple distinct states.[^11^, ^20^] In particular, memristors have emerged as capable of being utilized for neuromorphic computing.^[21]^ To date, these memristors have been realized mainly in solid‐state resistance‐switchable memristors as causing changes in conductance induced by dendritic metal paths in 2‐terminal memristors,^[22]^ or metal/insulator transitions in 3‐terminal memristors.^[23]^ Due to the limited biocompatibility of these solid‐state devices, polyelectrolyte‐confined fluidic memristors have recently received considerable attention to perform neuromorphic functions in aqueous media.^[8]^ In the following, we describe the rational design of all‐carbon capacitive memristors based on bioactive ions by specifically tuning the interactions and confinement within carbon nanopores. This all‐carbon device shares similar structures and mechanisms with other recently proposed all‐carbon iontronic devices (CAPode and G‐Cap),[^4^, ^20^] suggesting potential for further integration and application in logic computing. Compared to current devices, our proposed all‐carbon capacitive memristors offer excellent advantages in terms of cost, sustainability and biocompatibility.^[21]^

Various kinds of functionally bioactive ions are a prerequisite for complicated psychological, physiological, or behavioral processes; such ions include neurotransmitter ions in the human neural systems[^24^, ^25^] and natural phytohormones in plants.^[26]^ An understanding of the interactions between charged bioactive ions and polarized nanoporous carbon electrodes is crucial for the construction of bioactive ion‐based iontronics. The majority of bioactive ions in the human body are large and complex, which makes it difficult to explore their interplay with carbon electrodes.[^15^, ^27^] Consequently, it is necessary to develop bioactive ions with similar structures (such as cationic ammonium structures, which are common examples of bioactive cations)^[25]^ to determine the relationship between ionic structures and adsorption processes in porous carbons and to elucidate the charge balancing mechanisms of such bioactive ions in capacitive iontronic devices. It has been demonstrated that confined electrolyte ions feature memresistance and memcapacitance in iontronics.[^8^, ^28^] Strongly non‐linear hysteretic effects were predicted in confined ion transport as a theoretical basis for designing neuromorphic memristors.^[28]^ This inspired us to study the irreversible and strong physisorption of bioactive ions in nanoporous carbons to confine ions in a nanoporous structure and induce memresistance.

In our aim to rationally design carbon‐electrode interactions, we focus on a series of bioactive ionic compounds that are examples of ionic liquids (ILs) and serve as model compounds to investigate the adsorption and interaction mechanisms of bioactive ions and polarized nanoporous carbons. These compounds consist of an ammonium‐based cation with a naturally occurring (1*R*,2*S*,5*R*)‐(−)‐menthol moiety and an alkyl chain of various lengths (C_x_AmOMCl, where *x*=2, 6, and 12) paired with a chloride‐based anion.^[29]^ They also contain a previously undocumented compound, namely 2‐hydroxyethyl[(1*R*,2*S*,5*R*)‐(−)‐menthoxymethyl]dimethylammonium chloride (C_2‐OH_AmOMCl). We demonstrate the accessibility of the carbon nanopores via magic angle spinning nuclear magnetic resonance (MAS NMR) and in situ*‐*Raman spectroscopy. The latter results reveal the fast and irreversible physisorption of C_x_AmOMCl within the carbon nanopores. We further compare the memresistance behavior of the confined cations and weakly adsorbed choline cations in 2‐, 3‐, and 4‐terminal devices. Understanding the confinement and interaction mechanisms in nanopores is the basis for the rational design of ultracapacitor‐based ionic memristors.

## Results and Discussion

### Selection of Bioactive Ionic Compounds

**Scheme 1:**
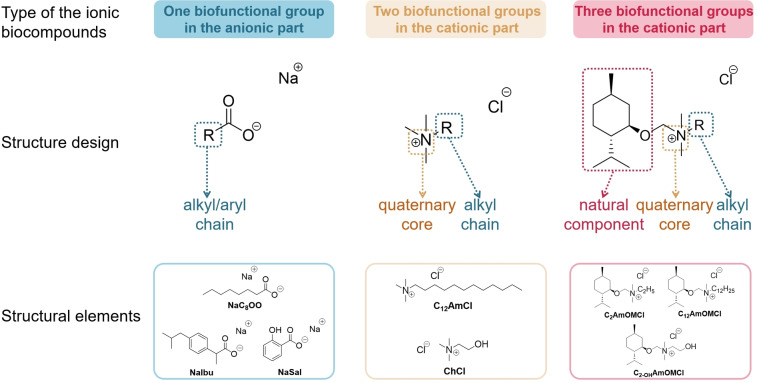
Approach for designing suitable bioactive ionic compounds for current work.

The group of compounds selected to demonstrate the role of interaction mechanisms is based on hierarchy and representation. On the one hand, we selected three representative levels of complexity regarding the number of biofunctional groups in either the anionic or cationic structural subsection (Scheme 1). On the other hand, it was important to select tunable motifs in which chain length, chirality, aromatic vs. aliphatic moieties, etc. can be systematically varied with a clear knowledge of the role of these structural elements in terms of bioactivity.[^30^, ^31^, ^32^] The bioactive compounds are classified into three groups based on their structural characteristics.

1. Well‐known and widely used drugs and/or drug components, including, but not limited to, sodium salicylate (NaSal),^[33]^ sodium ibuprofen (NaIbu),^[34]^ and sodium octanoate (NaC_8_OO).^[35]^ In this group of compounds, the anion is tunable. We also explored various modifications (defined in each case as one biofunctional group) adjacent to the ester group within the anion.
2. Quaternary ammonium salts. This group includes two well‐studied ionic compounds with biotechnological and medical activity: (*i*) dodecyltrimethylammonium chloride (C_12_AmCl)^[36]^ and (*ii*) choline chloride (ChCl).^[37]^ For the salts in this group, we isolated two components in the cation with biological activity: the aliphatic amine core^[30]^ and the type of chain.[^29^, ^38^] However, it should be emphasized that the choice of anions was not arbitrary; the presence of an anion increases these biomolecules’ water solubility and therefore affects their biodegradability and biocompatibility.^[39]^
3. Bioactive ILs synthesized in our group, namely (1*R*,2*S*,5*R*)‐(−)‐menthol‐based ammonium chlorides. It is crucial to emphasize that three factors influence the biofunctions of these ionic compounds: (*i*) the amine core;^[30]^ (*ii*) the length of the aliphatic chain;^[29]^ and (*iii*) the natural terpene component, including the effect of chirality or lack thereof.^[30]^ Furthermore, the additional role of chloride anions in enhancing the compounds’ biocompatibility^[30]^—mirroring the approach taken in the first group—is critical.

**Figure 1:**
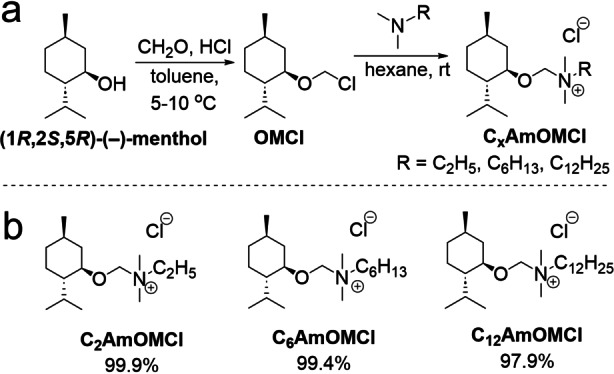
a) Procedure for synthesizing chloromethyl (1*R*,2*S*,5*R*)‐(−)‐menthyl ether (OMCl) and alkyl[(1*R*,2*S*,5*R*)‐(−)‐menthoxymethyl]dimethylammonium chlorides (C_x_AmOMCl). b) The structures of the synthesized ionic compounds with a (1*R*,2*S*,5*R*)‐(−)‐menthol moiety (C_x_AmOMCl).

The synthesis of alkyl [(1*R*,2*S*,5*R*)‐(−)‐menthoxymethyl]dimethylammonium chlorides (C_x_AmOMCl) is shown in Figure 1a. Three chiral quaternary ammonium salts with chloride as the anion and with even‐numbered alkyl chain lengths in the cation part—namely ethyl (C_2_AmOMCl), hexyl (C_6_AmOMCl), and dodecyl (C_12_AmOMCl)—were prepared via a two‐step approach under mild synthetic conditions (Figure 1b). (1*R*,2*S*,5*R*)‐(−)‐Menthol was selected as the primary building block for the cation because it is a naturally occurring compound with a wide range of bio‐applications in fields related to medical[^40^, ^41^] and biotechnology.^[42]^ A detailed description of the quaternization reaction and characterization of the resulting C_x_AmOMCl is provided in the Supporting Information. The physicochemical properties of the obtained quaternary ammonium salts revealed these salts^[29]^ to be ILs.^[43]^ Nuclear magnetic resonance (NMR) and mass spectra of the C_x_AmOMCl products (Figure S1–S19) confirmed the successful synthesis of all chiral ILs (CILs).

The homologous series of C_x_AmOMCl presented here exhibits a broad range of biological activity. These compounds are effective as antimicrobial agents against bacterial rods, cocci, bacilli, and fungi.[^29^, ^30^]

We also observe an enhancement in the biocidal effectiveness of C_x_AmOMCl against pathogenic bacteria and fungi in the presence of the (−) enantiomer of menthol in the structure; such an enhancement is not noted in their racemic counterparts.^[30]^ Additionally, it is worth mentioning that biotests reveal a strong correlation between the chain length and biological activity. C_x_AmOMCl salts with long alkyl chains (ranging from 9 to 12) engage in much higher biological activity than salts with shorter alkyl chains (ranging from 1 to 8). Notably, the biocompound selected as a prominent model system, dodecyl[(1*R*,2*S*,5*R*)‐(−)‐menthoxymethyl]dimethylammonium chloride (C_12_AmOMCl), demonstrates significantly higher antimicrobial activity compared to the commonly used benzalkonium chloride (BAC). For most of the Gram‐positive and Gram‐negative bacterial strains and yeast‐like fungi tested, the minimum inhibitory concentration (MIC) and minimum bactericidal/fungicidal concentration values for C_12_AmOMCl are significantly lower than those for BAC.^[30]^ For example, the MIC against *M. luteus* for C_12_AmOMCl is 0.5 μM, compared to 1.4 μM for BAC, and for *E. faecium*, the MIC is 0.5 μM for C_12_AmOMCl, compared to 5.6 μM for BAC. This result is particularly promising because long‐term use of BAC can lead to BAC resistance in various microbial isolates. These findings suggest that C_12_AmOMCl could be highly effective for antisepsis and disinfection purposes.

Furthermore, our findings reveal that CILs with (1*R*,2*S*,5*R*)‐(−)‐menthol featuring an aliphatic ammonium core exhibit the broadest spectrum of the most potent activity against microorganisms compared to other CILs with (1*R*,2*S*,5*R*)‐(−)‐menthol containing imidazolium or pyridinium cores. In our previous work, we found that C_12_AmOMCl has antifungal and antiadhesive properties against *C. albicans*.^[44]^ Our results reveal that this CIL is able to permeate the membranes of *C. albicans* and degrade their cell walls. For a compound to be considered a viable disinfectant, it must not cause hemolysis. Therefore, we tested the hemolytic activity of quaternary ammonium chloride with a dodecyl alkyl chain at its active concentrations for killing fungal cells, detaching adherent *C. albicans* cells from surfaces, inhibiting filamentation, permeabilizing membranes, and disrupting cell walls.^[44]^ Our results showed that C_12_AmOMCl has very low hemolytic activity, ranging from 3–12 % at concentrations of 10–100 μM, with 1 % sodium dodecyl sulfate serving as a positive control for 100 % hemolysis. The C_12_AmOMCl we investigated has potential as a disinfectant because it not only has antifungal and anti‐adhesive activity, but also does not induce hemolysis.

### Adsorption Characteristics of Ammonium‐Based Cations in Nanoporous Carbons

The calculated dimensions of the cations are summarized in Table S1.^[45]^ The pore size distribution of the chosen model carbon NORIT ROX 0.8 (termed ROX) is broad and ranges from 1–10 nm (Figure S20). The cation length of the selected bioactive ILs ranges from 0.86 nm (choline cations) to 2.48 nm (C_12_AmOM cations),^[45]^ which makes it possible to study ionic structures and adsorption behavior in ROX carbon electrodes. We investigated varying alkyl chain lengths to examine the interaction between the hydrophilic group of the bioactive ions and the surface of the carbon electrodes.

**Figure 2:**
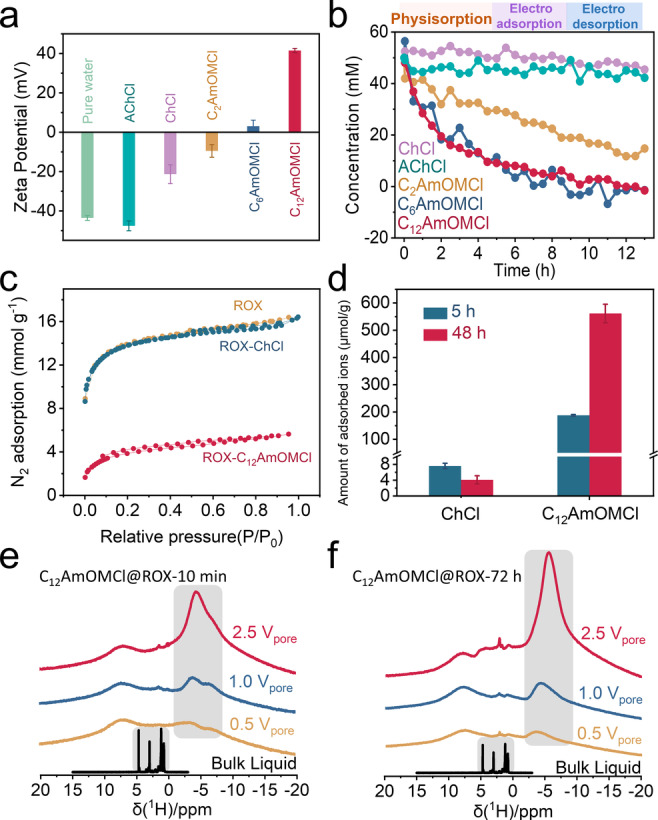
a) The zeta potentials of ROX carbon treated with different aqueous IL solutions (0.1 M). b) The concentration profiles of various aqueous solutions vs. time in ROX‐based symmetric supercapacitors. c) N_2_ physisorption isotherms of ROX, ROX‐ChCl, and ROX‐C_12_AmOMCl at 77 K. d) The amount of physically adsorbed ChCl and C_12_AmOMCl in ROX carbon after 5 and 48 h evaluated by LC‐MS. ^1^H MAS NMR spectra of defined amounts (0.5, 1.0, and 2.5 V_pore_) of 0.1 M C_12_AmOMCl ^2^H_2_O solution in ROX carbon powders e) without equilibration and f) after 72 h of equilibration time.

We evaluated the interaction between the selected bioactive ions and nanoporous carbon by measuring the zeta potential of carbon in suspension solutions; the carbon materials were treated with 0.1 M bioactive ion aqueous solutions (see Figure S21 and the experimental section for details of the zeta potential measurement). As shown in Figure 2a, the zeta potential for carbon with pure water treatment is roughly −43.6 mV, which reveals the negatively charged surface of ROX. The zeta potentials for acetylcholine chloride (AChCl)‐ and ChCl‐treated ROX carbon are −47.6 and −21.4 mV, suggesting a weak interaction of acetylcholine and choline cations with ROX. The zeta potentials of other bioactive ion‐treated ROX carbons exhibited an obvious increase and changed from −9.5 mV (C_2_AmOMCl), 3.1 mV (C_6_AmOMCl) to 41.5 mV (C_12_AmOMCl) as the alkyl chain increases from ethyl to dodecyl. This finding indicated increasing physisorption with increasing alkyl chain length.

In Figure 2b we compared the adsorption‐related properties of bioactive ions in porous carbon electrodes. Relying on an in situ*‐*Raman setup (Figure S22), we finely calibrated the bioactive ion concentration and the Raman intensity (Figure S23 and S24). For ChCl and AChCl (ChCl‐ and AChCl‐treated ROX carbons with negative zeta potentials of −21.4 and −47.6 mV, which indicates a weak interaction between the cations and nanoporous carbons), the measured concentrations exhibited a slight decrease after 13 h (5‐h of physisorption, 4‐h of electroadsorption, and 4‐h of electrodesorption; Figure 2b); the electrosorption‐induced concentration changes in a 0.01 M ChCl electrolyte have been confirmed via ex situ‐liquid chromatography‐mass spectrometry (LC‐MS).^[11]^ We noted a clear trend for stronger adsorption kinetics with increasing alkyl chain length according to our in situ*‐*Raman results (the fluctuations in concentration are mainly due to the limited signal‐to‐noise ratio of Raman spectroscopy under in situ*‐*testing conditions). For C_12_AmOMCl, there were only weak C_12_AmOM signals after 5 h (physisorption) according to the Raman spectra, and no signal was observed after 13 h (Figure S25). The adsorption kinetics increased significantly from C_2_AmOMCl to C_6_AmOMCl, and the corresponding zeta potential values changed from −9.5 mV (C_2_AmOMCl) to 3.1 mV (C_6_AmOMCl) (Figure 2a–b). C_12_AmOMCl exhibited similar adsorption behavior in the ROX electrode as C_6_AmOMCl. The zeta potential data and the in situ*‐*Raman spectra jointly indicated that the hydrophobic interaction depended on alkyl chain rather than on the presence of the monoterpene moiety; furthermore, the physisorption intensified with the increasing alkyl chain length. Notably, C_x_AmOMCl (where *x*=2, 6, and 12) that strongly physically adsorbed in nanoporous carbons could not be desorbed by electric polarization (Figure 2b).

We also treated ROX powders with 0.1 M ChCl and C_12_AmOMCl solutions (washed with pure water three times) and termed the final products ROX‐ChCl and ROX‐C_12_AmOMCl. These products were characterized via N_2_ physisorption at 77 K to evaluate the porosities of ROX after physisorption with ChCl and C_12_AmOMCl (Figure 2c). We noted almost no decrease in SSA of ROX‐ChCl (1162 m^2^ g^−1^) compared to the initial ROX (1182 m^2^ g^−1^). However, we observed a strong decrease in SSA for ROX‐C_12_AmOMCl (198 m^2^ g^−1^), which indicated a strong interaction between C_12_AmOM cations and ROX carbon powders. Given the size difference between ChCl and C_12_AmOMCl, the porosity difference may have also resulted from the large cation size of C_12_AmOMCl. Therefore, we used the independent LC‐MS experiments to confirm that the amount of physically adsorbed C_12_AmOMCl (up to 200 μmol/g after 5 h and 562 μmol/g after 48 h) was hundreds of times higher than that of ChCl (less than 5 μmol/g after 48 h), as shown in Figure 2d.

We employed ex situ‐^1^H MAS NMR spectroscopy to explore the physisorption of C_12_AmOMCl on ROX carbon powders with a broad pore size distribution at the molecular level. The well‐known ring current effect in graphitic carbon materials decreases the chemical shifts of adsorbed molecules.[^46^, ^47^, ^48^, ^49^, ^50^] By studying those so‐called nuclear‐independent chemical shifts (NICS), we were able to discriminate between molecules within the pores of the carbon materials and those in the surrounding bulk electrolyte. ^1^H MAS NMR spectra of ROX loaded with defined amounts of 0.1 M C_12_AmOMCl ^2^H_2_O solutions relative to the available pore volumes (V_pore_) of ROX are shown in Figure 2e and 2f. For the samples shown in Figure 2e, we conducted measurements immediately after we had loaded the carbon material and did not allow for any additional equilibration time except for the sample transfer time necessary to move the sample into the spectrometer (ca. 10 minutes). The alkyl groups of C_12_AmOMCl are well resolved in the bulk phase and occurred between 1 and 5 ppm. They exhibited negative chemical shifts and two poorly resolved maxima at −3.8 and −6.7 ppm for the three different loading volumes, i.e., the signals exhibited severe line broadening in addition to a negative shift. The negative chemical shift resulted from the ring current effect noted above; it revealed that all of the C_12_AmOMCl molecules were adsorbed within the pores immediately after loading. The pronounced line broadening is caused by the immobilization of the adsorbed molecules within the pores; this broadening might be a sign of different states of the adsorbed molecules, which could have resulted in a distribution of chemical shift. After 72 h of equilibration (Figure 2f), the lines of the adsorbed C_12_AmOMCl molecules at negative chemical shifts became somewhat narrower and were then centered in one primary peak. This result attested to a slow redistribution of cations within the pores of the ROX carbon. A larger amount of C_12_AmOMCl electrolyte resulted in more negative chemical shifts (i.e., −3.7 ppm for 0.5 V_pore_, −4.1 ppm for 1.0 V_pore_, and −5.5 ppm for 2.5 V_pore_). In summary, ^1^H MAS NMR spectroscopy revealed a rapid uptake of C_12_AmOMCl by the ROX carbon material. However, the adsorbed molecules rearranged themselves slowly within the material over a longer period of time.

The mechanism of C_x_AmOMCl interaction with ROX carbon was further evaluated by comparing C_2‐OH_AmOMCl and C_2_AmOMCl. The presence of the OH group in C_2‐OH_AmOMCl weakened the adsorption of molecules in porous carbons with hydrophobic surfaces, which also confirmed that the interacting group of the bioactive cations for the exchange of C_2_AmOMCl to C_12_AmOMCl was the alkyl chain rather than the monoterpene moiety (Figure S26–36 and Section 2 in the Supporting Information). Additionally, all of these CILs we investigated were electrochemically stable under electric polarization (Figure S37–S39).

### Investigation on the Synergistic Adsorption Effect of Ions in Nanoporous Carbons

**Figure 3:**
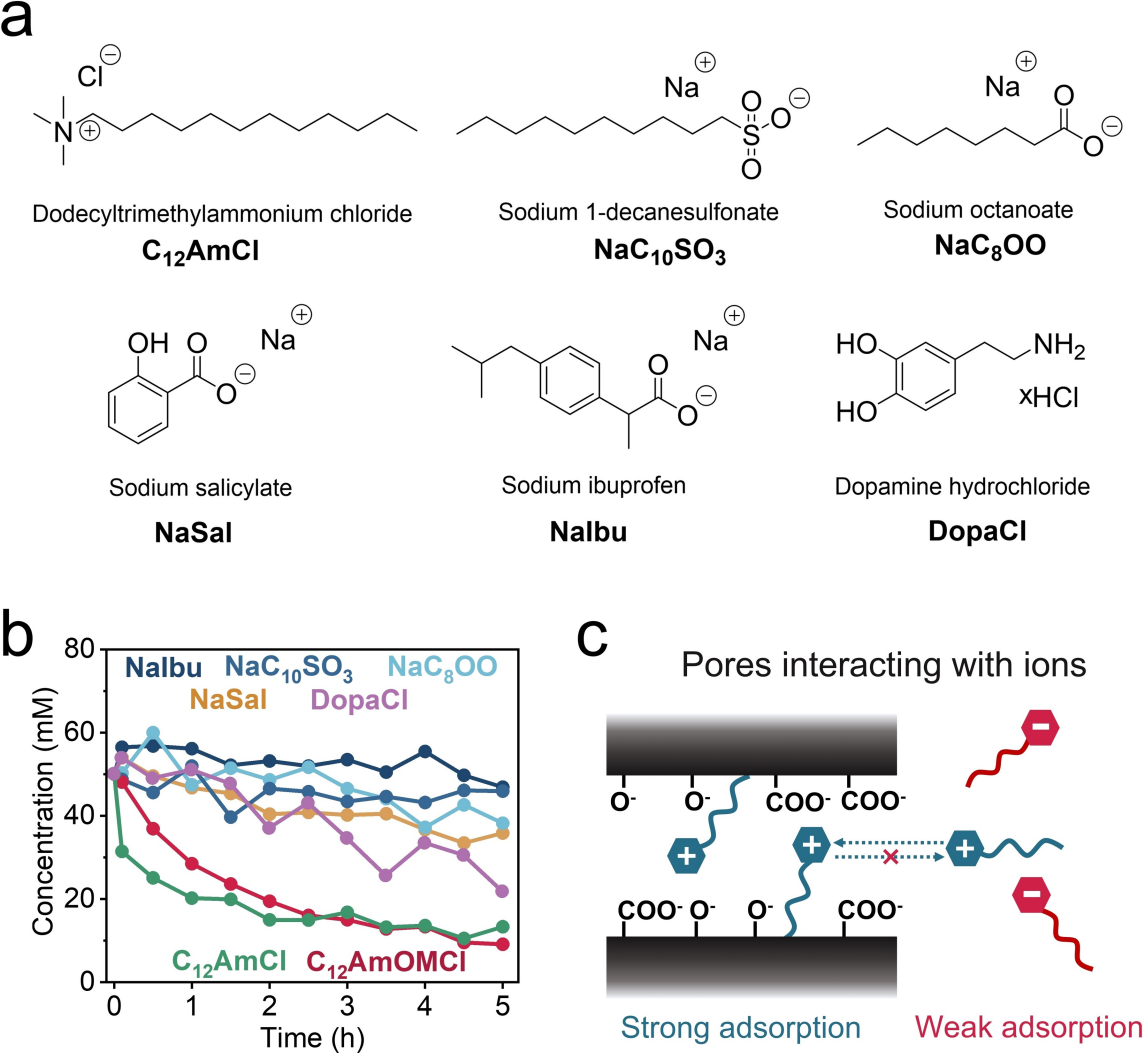
a) Chemical structures of three organic ions with long alkyl chains and three bioactive ions. b) Concentration profiles vs. time for selected ion‐based aqueous solutions induced by the physisorption of porous carbon (ROX). c) Schematic diagram of the strong physisorption behavior induced by the hydrophobic effect and electrostatic attraction.

We have shown that, based on the interaction behavior of bioactive ammonium‐based cations with nanoporous carbons, that a longer alkyl chain led to stronger irreversible physisorption. We selected six typical organic compounds, C_12_AmCl, sodium 1‐decanesulfonate (NaC_10_SO_3_), NaC_8_OO, NaSal, NaIbu, and dopamine hydrochloride (DopaCl), to further investigate the ion physisorption behavior in ROX carbon (Figure 3a) via Raman spectroscopy (see the calibration curves in Figure S40). C_12_AmCl and C_12_AmOMCl exhibited similar interaction behaviors with ROX carbon, which suggested that the alkyl chain length played a key role in the strong physisorption of cations with ROX carbon (Figure 3b).

In the context of other bioactive ionic compounds that do not belong to quaternary ammonium salts, we observed a variety of interaction behaviors of anions with long alkyl chains in NaC_10_SO_3_ and NaC_8_OO; however, we observed only a slight decrease in concentration for NaC_10_SO_3_ and NaC_8_OO compared to C_12_AmOMCl after 5 h (Figure 3b). For these compounds, a long alkyl chain was apparently not a sufficient and necessary condition for strong and irreversible physisorption of C_x_AmOMCl; physisorption is caused by the synergistic effect of hydrophobic and electrostatic attraction (Figure 3c). ROX carbon is a form of activated carbon with negative surface functional groups (such as ‐O^−^ and ‐COO^−^).^[51]^ It has a negative zeta potential (−43.6 mV) (Figure 2a and 3c). C_12_AmOM cations will accordingly adsorb on the surface of ROX via electrostatic attraction, and then the hydrophobic alkyl chain will be attracted to the carbon surface via hydrophobic interactions. That situation leads to strong and irreversible physisorption between ROX carbon and C_12_AmOM cations. However, for anions with long alkyl chains, such as C_10_SO_3_^‐^ and C_8_OO^‐^ anions, the electrostatic repulsive force is responsible for suppressing the adsorption on the negatively charged ROX carbon surface, which results in relatively weak physisorption compared to that observed for C_12_AmOM cations.

We next investigated three commercially important and common bioactive ionic compounds—NaSal, NaIbu, and DopaCl—to examine the theory of the synergistic adsorption effect via in situ*‐*Raman experiments (Figure 3b). The salicylate and ibuprofen anions exhibited smaller concentration changes than C_12_AmOM cations after 5 h of physisorption. Dopamine cations with two OH groups exhibited slightly stronger interactions with ROX carbon than salicylate and ibuprofen anions. Negative zeta potentials are typical for ROX carbon and other common nanoporous carbons, and the hypothesis was also verified for YP‐50F and AC‐CS carbons (Figure S41 and Section 2 in the Supporting Information).

### Irreversible‐Physisorption Induced Confined Cations and Relative Ionic Memresistance

**Figure 4:**
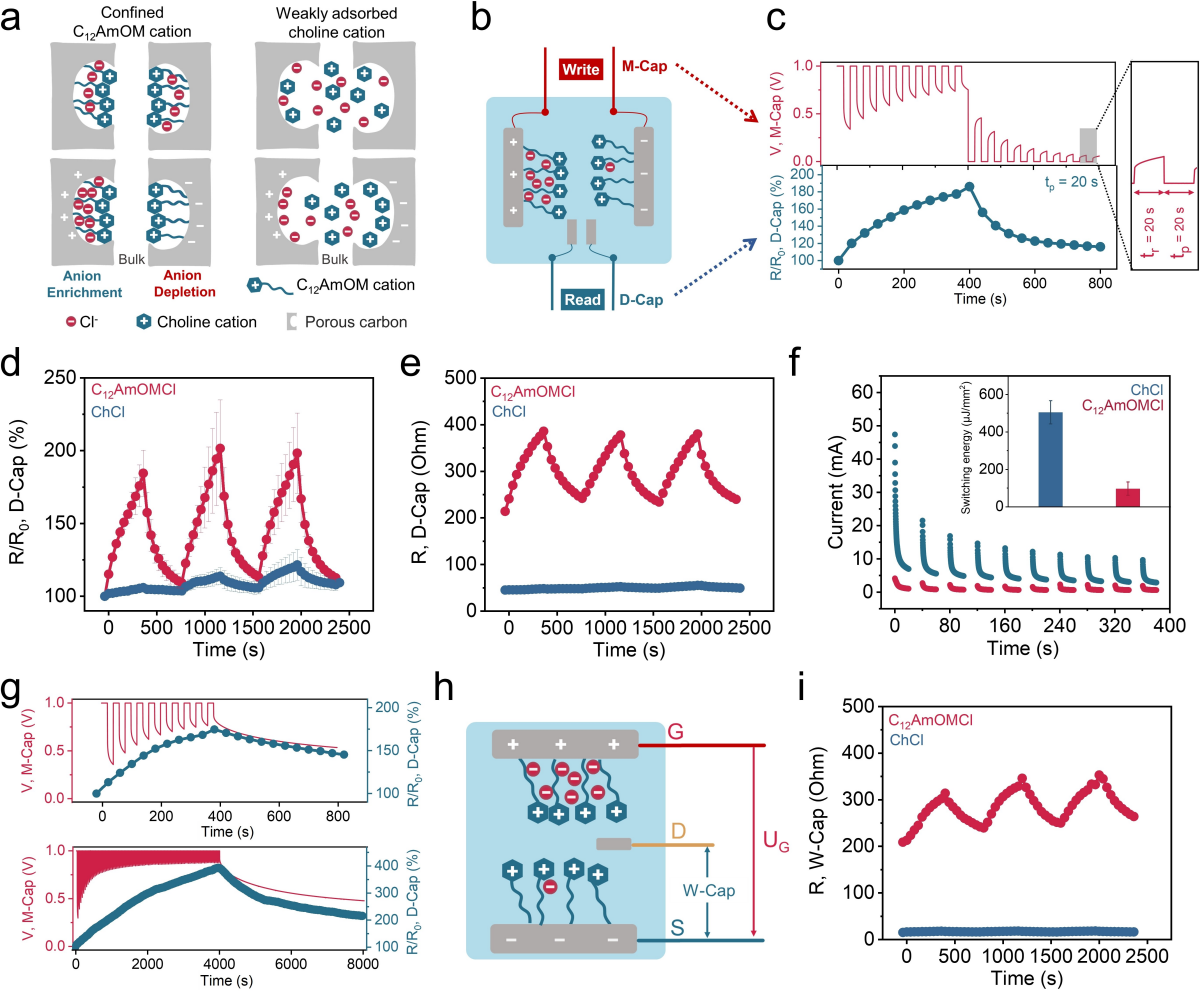
a) Schematic diagram of the different ionic interactions induced by strong (i.e., C_12_AmOM cations) and weak (i.e., choline cations) physisorption. b) Schematic diagram of a 4‐terminal ionic memristor in a C_12_AmOMCl electrolyte. c) The resistance retention (R/R_0_, R_0_: the initial resistance of D‐Cap) of the D‐Cap during voltage pulsing (10 times of 1 V‐pulses and 10 times of 0 V‐pulses) to the M‐Cap (t_p_: pulse time, t_r_: rest time) in a 0.1 M C_12_AmOMCl electrolyte. d) The switching resistance retention (R/R_0_) of D‐Cap during voltage pulsing to the M‐Cap (t_p_=20 s, t_r_=20 s) in 0.1 M C_12_AmOMCl and 0.1 M ChCl electrolytes, and e) the corresponding actual value of the resistance changes of D‐Cap. f) I‐t curves of M‐Cap during 10 times of 1 V‐pulses (t_p_=20 s, t_r_=20 s) in 0.1 M C_12_AmOMCl and ChCl electrolytes and the average switching energy of 1 pulse. g) The resistance state of D‐Cap after 10 and 100 times of 1 V‐pulses (t_p_=20 s, t_r_=20 s) to the M‐Cap in a 0.1 M C_12_AmOMCl electrolyte. h) Schematic diagram of a 3‐terminal ionic memristor in a C_12_AmOMCl electrolyte. i) The switching resistance of W‐Cap during voltage pulsing to U_G_ (t_p_=20 s, t_r_=20 s) in 0.1 M C_12_AmOMCl and ChCl electrolytes.

Due to the irreversible physisorption, C_12_AmOM cations were strongly adsorbed and confined by ROX carbon; chloride anions remained mobile in the solution. During electric polarization, C_12_AmOM cations were fixed on the surface of the polarized carbon electrodes; the chloride anions were adsorbed and desorbed to compensate the charges (Figure 4a). Confining the C_12_AmOM cations resulted in anion enrichment on the positive electrode and anion depletion on the negative electrode during electric polarization, which led to memristive behavior. For the weak interaction mode with ChCl, both choline cations and chloride anions contribute to balancing the opposite charges (Figure 4a). Benefiting from this phenomenon, we developed the all‐carbon ionic memristor shown in Figure 4b. It is composed of an outer main capacitor (M‐Cap, with two large ROX carbon electrodes) for ion electrosorption and an inner detective capacitor (D‐Cap, with two small ROX carbon electrodes) for detecting the ions’ dynamics and relative changes in resistance. We explored different electrolyte systems to study the resistance changes of the D‐Cap while deliberately controlling the M‐Cap voltage (Figure 4c–f). We applied ten 1 V‐pulses followed by ten 0 V‐pulses with the same pulse time (t_p_=20 s) to the M‐Cap in a 0.1 M C_12_AmOMCl electrolyte (Figure 4c). We evaluated the resistances of the D‐Cap using electrochemical impedance spectroscopy (EIS) during the rest time (t_r_=20 s, no bias voltage was applied to the M‐Cap; only the open circuit voltage was recorded).

We noted a 15 % and 85 % increase in resistance of the D‐Cap (compared to its initial resistance) after 1 and 10 times of 1 V‐pulses. We also compared the resistance changes for the D‐Cap in C_12_AmOMCl and ChCl electrolytes (Figure 4d). Due to variations in interaction behaviors of C_12_AmOMCl and ChCl with ROX carbon, we observed pronounced resistance changes in the D‐Cap in 0.1 M C_12_AmOMCl and ChCl electrolytes. While we observed an 85 % increase in resistance of the D‐Cap after the first ten 1 V‐pulses in a 0.1 M C_12_AmOMCl electrolyte, only a 6 % increase was noted in a 0.1 M ChCl electrolyte. The actual values of resistance changes and impedance spectra are shown in Figure 4e and S42. That difference is due to variations in the numbers of confined C_12_AmOM cations and weakly adsorbed choline cations; the confinement of C_12_AmOM cations—additionally led to anion enrichment and anion depletion during electric polarization (Figure 4a). In addition, we observed a 9 % increase in resistance for the first ten 1 V‐pulses in NaC_10_SO_3_, which has a weak interaction with ROX carbon even with a long chain in the anionic moiety (Figure S43). We further evaluated the switching energy of these 4‐terminal devices in C_12_AmOMCl and ChCl electrolytes by integrating the area of I‐t curves of M‐Cap. The average values of switching energy for 1 time of 1 V‐pulse are 504.8 and 97.1 μJ/mm^2^, for 0.1 M ChCl and C_12_AmOMCl electrolytes, respectively (Figure 4f). By decreasing the mass loading of M‐Cap (from 60 to 10 mg) and concentration of C_12_AmOMCl electrolyte (from 0.1 to 0.01 M), the switching energy decreased from 97.1 to 1.5 μJ/mm^2^ for 1 time of 1 V‐pulse (Figure S44). The memory time of this 4‐terminal ionic memristor was investigated and the resistance state of D‐Cap after 1, 10, and 100 times of 1 V‐pulses to the M‐Cap was evaluated in Figure 4g and S45. After one 1 V‐pulse, there was an 18 % increase in resistance of the D‐Cap, and the resistance gradually decreased to 7 % after 240 s. After ten subsequent 1 V‐pulses, we noted a 75 % increase in resistance of the D‐Cap, and a similar decrease in resistance (to 45 %) was observed after 400 s. When increasing pulse times to 100, there was a 293 % increase in resistance of D‐Cap, which decreased to 216 % of the initial resistance after 4000 s. These results indicate a nonvolatile characteristic of the capacitive ionic memristor, which is determined by confined ion transport and sorption rates in the pores of polarized nanoporous carbons. There is no pronounced change in the memory time using a polyvinyl alcohol gel electrolyte demonstrating that ion diffusion in the bulk electrolyte plays only a minor role for the memory time (Figure S46).

4‐terminal devices facilitate the understanding of the mechanism of resistance change induced by electrically‐driven ion adsorption. In addition, 2‐ and 3‐terminal devices as common types of ionic memristors were investigated in Figure 4h–i and Figure S47–48. In a 3‐terminal device (Figure 4h), two large electrodes were used as gate (G) and source (S) electrodes (the voltage applied to G and S was denoted U_G_) to effectively control ion adsorption. S electrode and a third small electrode (drain (D) electrode) form a working capacitor (W‐Cap). After ten subsequent 1 V‐pulses to G and S electrodes, the resistance of W‐Cap showed a similar increasing trend as D‐Cap in a 4‐terminal device (Figure 4i and S47). For a C_12_AmOMCl electrolyte, we observed a 47 % increase in the resistance of W‐Cap in this 3‐terminal device, which is lower than that for a 4‐terminal device (85 %). However, the increase for the ChCl electrolyte (29 %) was higher than that for a 4‐terminal device (15 %). The difference is due to the different resistance detected in 3‐ and 4‐terminal devices (Figure 4b and h). The resistance changes in 2‐terminal devices were also detected and compared for ChCl and C_12_AmOMCl electrolytes (Figure S48), which further confirmed that the resistance changes resulted from electrically‐driven ion adsorption. Moreover, we compared the memory time of 2‐, 3‐, and 4‐terminal memristors (Figure S49 and Section 2 in the Supporting Information). These results demonstrate that ion confinement in nanoporous carbons provides a generalized strategic concept and platform for various memristive device architectures.

### Electrically‐Driven Concentration Change and Resistance Switching Behaviour

**Figure 5:**
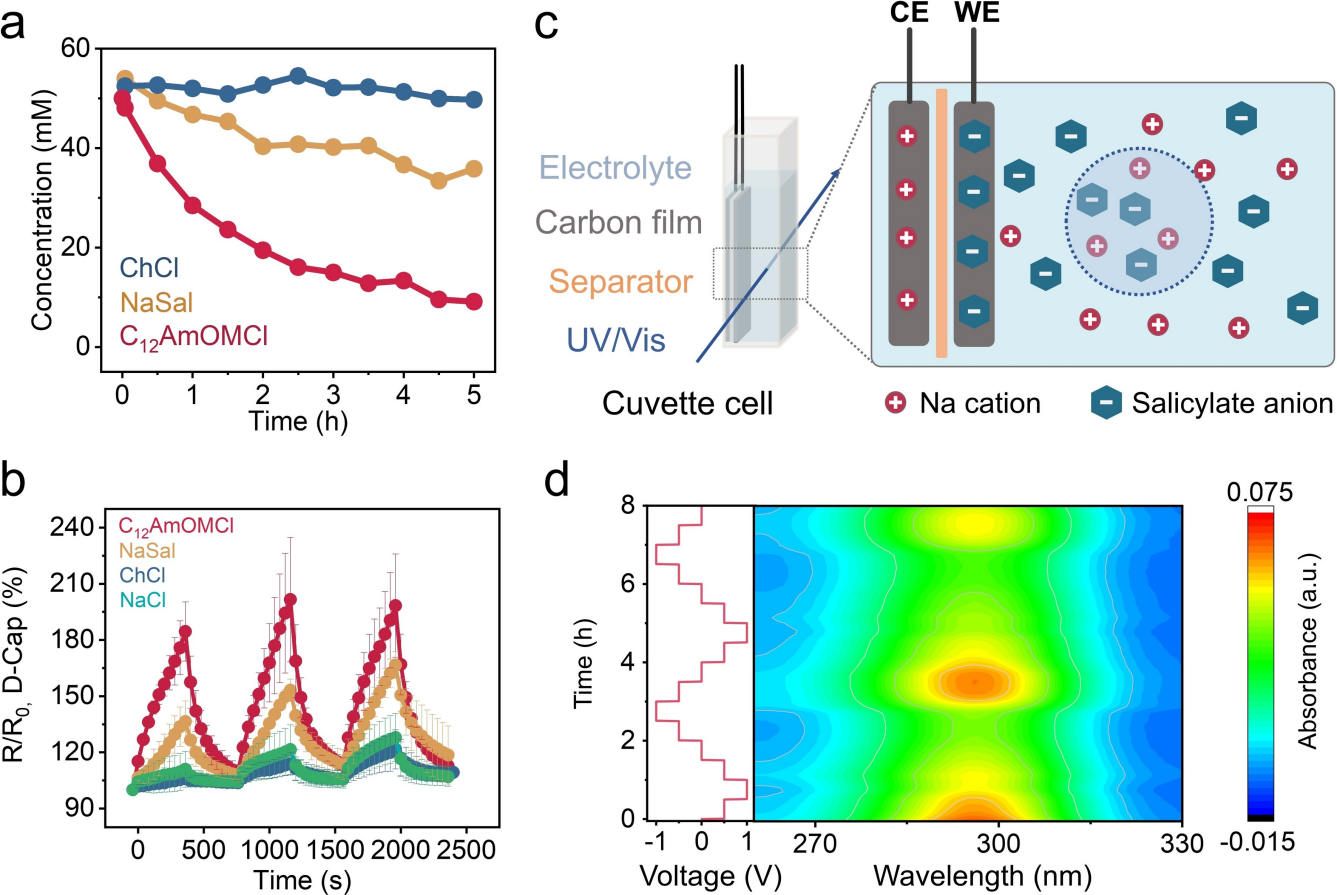
a) Concentration profiles vs. time for ChCl, NaSal, and C_12_AmOMCl aqueous solutions induced by the physisorption of porous carbon (ROX). b) The switching resistance retention (R/R_0_) of D‐Cap during voltage pulsing (10 times of 1 V‐pulses and 10 times of 0 V‐pulses) to the M‐Cap (t_p_=20 s, t_r_=20 s) in 0.1 M C_12_AmOMCl, NaSal, ChCl, and NaCl electrolytes in 4‐terminal devices. c) Schematic diagram of a cuvette cell for NaSal detection. d) Contour plot of UV/Vis spectra of the in situ*‐*cuvette cell with 1 mM NaSal as electrolyte.

Different interactions of C_12_AmOMCl and ChCl lead to the different switching behavior of the resistance and chloride anions remain mobile in both electrolytes. As it is challenging to monitor the local concentration of chloride anions spectroscopically, we selected salicylate anions as a model to investigate the concentration fluctuation induced by electric polarization, based on the characteristic ultraviolet (UV) absorption of salicylate anions (see the calibration curves in Figure S50). The physisorption of NaSal was compared with ChCl and C_12_AmOMCl via in situ*‐*Raman spectra (Figure 5a). We observed the physisorption behavior in ROX carbon, which is intermediate between the ChCl and C_12_AmOMCl, due to the aromatic structure (hydrophobic interaction) of salicylate anions (electrostatic repulsion). The increase in resistance of D‐Cap in a NaSal electrolyte (36 %) was also higher than that in NaCl (11 %) and ChCl (6 %) electrolytes, and lower compared to C_12_AmOMCl electrolyte after applying ten 1 V‐pulses to the M‐Cap (Figure 5b).

The electrically‐driven concentration fluctuation was evaluated in a cuvette cell (Figure 5c). Two ROX carbon electrodes and a separator were assembled inside the cuvette. The concentration of the bulk electrolyte was monitored by ultraviolet‐visible (UV/Vis) spectroscopy after 3 days of equilibration time (Figure 5d). When the cell voltage was increased to 1 V (working electrode (WE) was positively polarized, and counter electrode (CE) was negatively polarized), salicylate anions in the bulk electrolyte were adsorbed by WE and there was a decreasing trend in intensity (concentrations of salicylate anions). When the cell voltage was decreased to −1 V, WE was negatively polarized, salicylate anions were desorbed and reinjected into the bulk solutions (i.e., an increase in concentrations, Figure 5d). The in situ*‐*UV/Vis results indicate that the controlled ion adsorption can be controlled by electric signals. The electrically‐driven concentration changes lead to resistance changes in the proposed capacitive ionic memristor devices, in response to the dynamic ion adsorption processes.

## Conclusion

Modulating the mobility of ions is a key aspect of iontronics. In this work, we demonstrated a novel all‐carbon capacitive ionic memristor principle operating with bioactive ions in all‐carbon ultracapacitor devices. By exploring the general interaction mechanisms and immobilization of various bioactive ions within nanoporous carbons, we confirmed that the hydrophobic interaction and electrostatic attraction act synergistically when bioactive ions are adsorbed in nanoporous carbons. The irreversible physisorption of cations with long alkyl chains resulted in confined cations immobilized in the carbon nanopores. This immobilization leads to electrolyte depletion and an anion dominated charge‐discharge mechanism, resulting in ionic memresistance properties observed in a 4‐terminal ionic memristor device with nonvolatile characteristics. In contrast, ions with weak interactions with carbon electrodes cause only minor memristive signatures. The relationship between the resistance state of the memristor and electrically‐driven bioactive ion adsorption was demonstrated via in situ*‐*UV/Vis spectroscopy. In addition, 2‐ and 3‐terminal devices also exhibit similar memristance characteristic as 4‐terminal devices. Our proposed all‐carbon memristor devices share mechanistic features and structures with reported ionic logic elements (such as CAPode and G‐Cap), which facilitates further integration and ionic logic computing application. The ability to deliberately control biomolecular species concentrations by electroadsorptive dosing in resistive states carrying information signals is a unique feature. Such memristive architectures operating with bioactive ions pave the way to enable bioelectronic devices for organism‐machine interfacing and neuromorphic computing via iontronic devices.

## Supporting Information

The authors have cited additional references within the Supporting Information.[^11^, ^29^, ^45^, ^51^, ^52^]

## Conflict of Interests

The authors declare no conflict of interest.

1

## Supporting information

As a service to our authors and readers, this journal provides supporting information supplied by the authors. Such materials are peer reviewed and may be re‐organized for online delivery, but are not copy‐edited or typeset. Technical support issues arising from supporting information (other than missing files) should be addressed to the authors.

Supporting Information

## Data Availability

The data that support the findings of this study are available in the supplementary material of this article.
