# Supplementary material for: Bioactive Ion‐Confined Ultracapacitive Memristors with Neuromorphic Functions
Source: Angew Chem Int Ed Engl. 2024 Nov 7;63(51):e202412674. doi: 10.1002/anie.202412674 (PMC11627131; doi:10.1002/anie.202412674)
Supplement: Supplementary file 1 — Supporting Information [file ANIE-63-e202412674-s001.pdf]

Angewandte Chemie
Eine Zeitschrift der Gesellschaft Deutscher Chemiker

Supporting Information

Bioactive Ion-Confined Ultracapacitive Memristors with Neuromorphic Functions

*P. Li, J. Feder-Kubis, J. Kunigkeit, M. Zielińska-Błajet, E. Brunner, J. Grothe, S. Kaskel**

Supporting Information

Bioactive Ion-Confined Ultracapacitive Memristors with Neuromorphic Functions

Panlong Li, Joanna Feder-Kubis, Jonas Kunigkeit, Mariola Zielińska-Błajet, Eike Brunner, Julia Grothe, Stefan Kaskel*

Abstract: The field of bioinspired iontronics, bridging electronic devices and ionic systems, has multiple biological applications. Carbon-based ultracapacitive devices hold promise for controlling bioactive ions via electric double layers due to their high-surface-area and biocompatible porous carbon electrodes. However, the interplay between complex bioactive ions and porous carbons remains unclear due to the variety of structures of bioactive ions present in biological systems. Herein, we investigate the adsorption behavior of a series of bioactive ammonium-based cations with varying alkyl chain lengths in nanoporous carbons. We find that strong physisorption results from the synergistic hydrophobic interaction and electrostatic attraction between porous carbons (with a negative zeta potential) and bioactive cations. Bioactive cations with varying alkyl chain lengths can be irreversibly physically adsorbed and confined within nanoporous carbons resulting in anion enrichment and depletion during electric polarization. This situation, in turn, results in a characteristic memristive behavior in all-carbon capacitive ionic memristor devices. Our findings highlight the relationship between the resistance state of the memristor and ion adsorption mechanisms in all-carbon capacitive devices, which hold potential for future transmitter delivery, biointerfacing, and neuromorphic devices.

Table of Contents

1.Experimental procedures……4
1.1. Materials……4
1.2. Methods of analysis and characterization……4
1.2.1. NMR measurement……4
1.2.2. Melting point measurement……4
1.2.3. Cationic active substance content……4
1.2.4. Electrode preparation……4
1.2.5. 2-Electrode capacitor and cyclic voltammetry……4
1.2.6. 3-Electrode measurement……4
1.2.7. Linear sweep voltammetry (LSV)……4
1.2.8. Zeta potential measurement……4
1.2.9. Physisorption evaluation and N_2 physisorption……4
1.2.10. *In situ*-Raman and *in situ*-UV/Vis *experiments*……5
1.2.11. 2-, 3-, and 4-Terminal device construction and resistance switching measurement……5
1.3. General methods for synthesis and storage conditions……5
1.3.1. Synthesis of alkyl[(1*R*,2*S*,5*R*)-(–)-menthoxymethyl]dimethylammonium chlorides (C_xAmOMCl)……5
1.3.2. Synthesis of 2-hydroxyethyl[(1*R*,2*S*,5*R*)-(–)-menthoxymethyl]dimethylammonium chloride ($C_{2\text{-}OH}$AmOMCl)……6
2. Results and Discussion……6
3. Supplementary Figures and Tables……7
Figure S1. ^{1}H NMR (300 MHz) spectra for C_2AmOMCl in D_2O+TMS……8
Figure S2. $^{13}C\{^1H\}$ NMR (151 MHz) spectra for C_2AmOMCl in D_2O+TMS……9
Figure S3. ^{13}C DEPT-135° NMR experiments (151 MHz, D_2O) for C_2AmOMCl……10
Figure S4. $^1H,^1H$ COSY experiment (600 MHz) for C_2AmOMCl in D_2O……10
Figure S5. $^1H,^{13}C$ multiplicity-resolved HSQC (CH_2-blue, CH/CH_3-red) experiments for C_2AmOMCl in D_2O……11
Figure S6. $^1H,^{13}C$ multiplicity-resolved HMBC experiments for C_2AmOMCl in D_2O……11
Figure S7. ^{1}H (600 MHz) NMR spectra for C_6AmOMCl in D_2O+TMS……13
Figure S8. $^{13}C\{^1H\}$ NMR (151 MHz) spectra for C_6AmOMCl in D_2O+TMS……14
Figure S9. ^{13}C DEPT-135° NMR experiments (151 MHz, D_2O) for C_6AmOMCl……14
Figure S10. $^1H,^1H$ COSY experiment (600 MHz) for C_6AmOMCl in D_2O……15
Figure S11. $^1H,^{13}C$ multiplicity-resolved HSQC (CH_2-blue, CH/CH_3-red) experiments for C_6AmOMCl in D_2O……15
Figure S12. $^1H,^{13}C$ multiplicity-resolved HMBC experiments for C_6AmOMCl in D_2O……16
Figure S13. ^{1}H NMR (600 MHz) spectra for C_{12}AmOMCl in D_2O+TMS……17
Figure S14. $^{13}C\{^1H\}$ NMR (151 MHz) spectra for C_{12}AmOMCl in D_2O+TMS……18
Figure S15. ^{13}C DEPT-135° NMR experiments (151 MHz, D_2O) for C_{12}AmOMCl……19
Figure S16. $^1H,^1H$ COSY experiment (600 MHz) for C_{12}AmOMCl in D_2O……19
Figure S17. $^1H,^{13}C$ multiplicity-resolved HSQC (CH_2-blue, CH/CH_3-red) experiments for C_{12}AmOMCl in D_2O……20
Figure S18. $^1H,^{13}C$ multiplicity-resolved HMBC experiments for C_2AmOMCl in D_2O……20
Figure S19. Mass spectra of synthesized bioactive ionic liquids……21
Figure S20. Pore size distribution of ROX carbon based on N_2 physisorption at 77 K……21
Figure S21. Schematic diagram of ROX carbons treated with different ionic liquid aqueous solutions……22
Figure S22. Schematic diagram of *in situ*-Raman setup……22
Figure S23. Raman data for ChCl and AChCl……22
Figure S24. Raman data for C_2AmOMCl, C_6AmOMCl, and C_{12}AmOMCl……23
Figure S25. *In situ*-Raman data of C_{12}AmOMCl……23
Figure S26. Synthesis of $C_{2\text{-}OH}$AmOMCl……24
Figure S27. ^{1}H (600 MHz) spectra for $C_{2\text{-}OH}$AmOMCl in D_2O+TMS……26
Figure S28. $^{13}C\{^1H\}$ NMR (151 MHz) spectra for $C_{2\text{-}OH}$AmOMCl in D_2O+TMS……26
Figure S29. ^{13}C DEPT-135° NMR experiments (151 MHz, D_2O) for $C_{2\text{-}OH}$AmOMCl……27
Figure S30. $^1H,^1H$ COSY experiment (600 MHz) for $C_{2\text{-}OH}$AmOMCl in D_2O……27
Figure S31. $^1H,^{13}C$ multiplicity-resolved HSQC (CH_2-blue, CH/CH_3-red) experiments for $C_{2\text{-}OH}$AmOMCl in D_2O……28
Figure S32. $^1H,^{13}C$ multiplicity-resolved HMBC experiments for $C_{2\text{-}OH}$AmOMCl in D_2O……28
Figure S33. Mass spectrum of $C_{2\text{-}OH}$AmOMCl……29
Figure S34. The comparison of zeta potentials……29
Figure S35. Raman data for $C_{2\text{-}OH}$AmOMCl……30
Figure S36. The comparison of C_2AmOMCl and $C_{2\text{-}OH}$AmOMCl……30
Figure S37. Capacitance evaluation……31
Figure S38. LSV measurement……31

Figure S39. *Ex situ*-liquid NMR data……32
Figure S40. Raman data for NaC_8OO, $NaC_{10}SO_3$, NaSal, NaIbu, $C_{12}AmCl$, and DopaCl……32
Figure S41. The interaction investigation of AC-CS and YP-50F nanoporous carbons……33
Figure S42. The experimental impedance data of D-Cap in 4-terminal devices……33
Figure S43. The switching resistance retention in $C_{12}AmOMCl$ and $NaC_{10}SO_3$ electrolytes……33
Figure S44. The investigation of switching energy……34
Figure S45. The resistance state of D-Cap after 1 time of 1 V-pulses (t_p = 20 s) to the M-Cap in a 0.1 M $C_{12}AmOMCl$ electrolyte……34
Figure S46. The resistance retention comparison with/without polyvinyl alcohol (PVA) gel……34
Figure S47. The resistance changes in 3-terminal devices……35
Figure S48. The resistance changes in 2-terminal devices……35
Figure S49. The comparison of resistance retention in 2-, 3-, and 4-terminal devices……35
Figure S50. UV/Vis spectra of NaSal solutions and the calibration curves under various wavelengths……36
Table S1. Chemical information of all synthesized ionic compounds and purchased ChCl and AChCl……36
Reference……37
Author Contributions……37

1. Experimental Procedures

1.1 Materials

The following materials for the synthesis of bioactive ionic compounds were purchased from Merck: (1*R*,2*S*,5*R*)-(–)-menthol (99%), *N*,*N*-dimethylethylamine (≥99%), *N*,*N*-dimethylhexylamine (98%), *N*,*N*-dimethyldodecylamine (97%), *N*,*N*-dimethyl-2-hydroxyethylamine (≥99.5%), paraformaldehyde (powder, 95%), hydrochloric acid (35-38%), sulfuric acid (≥96%).

Drying agents, such as sodium sulfate (anhydrous, pure) and phosphorus pentoxide (powder, anhydrous, ≥98%), were purchased from Alchem (Gliwice, Poland). Deuterium oxide (deuteration degree min. 99.9% for NMR spectroscopy), purchased from Merck, was used for NMR analysis. All commonly used organic solvents were supplied by Alchem, Merck and Fluka and were dried before synthesis. All the reagents were purified and dried according to standard procedures and stored over molecular sieves.

The commercially available compounds tested in our work, such as choline chloride (ChCl), sodium salicylate (NaSal), ibuprofen sodium (NaIbu), sodium 1-decanesulfonate ($NaC_{10}SO_3$), and sodium octanoate (NaC_8OO), as well as the hydrophilic PVDF membrane with a pore size of 0.1 µm and glass microfiber filters (Whatman), were purchased from Merck (Germany). Polytetrafluoroethylene (PTFE) was purchased from ABCR (Karlsruhe, Germany). Carbon ink (DAG EB-012) was purchased from Ladd Research Industries (USA). Multi-walled carbon nanotubes (MWCNT) were purchased from TCI Deutschland GmbH. ROX carbon extrudates were provided by Norit Group (Germany). Titanium (Ti) wire and mesh were purchased from Guangdong Canrd New Energy Technology (China).

1.2 Methods of analysis and characterization

1.2.1. NMR measurement

The structures and purity of all of the synthesized compounds were confirmed by spectral analysis. The ^{1}H NMR and ^{13}C NMR spectra were recorded on a Bruker DRX with tetramethylsilane as the standard (at 300 or 600 MHz for ^{1}H NMR, and 151 MHz for ^{13}C NMR). MAS NMR analysis was performed according to formed report.[1]

1.2.2. Melting point measurement

Melting points were determined using a model JA 9100 electrothermal digital melting point apparatus. Optical rotations at 578 nm were measured with an Optical Activity Ltd. Model AA-5 automatic polarimeter.

1.2.3. Cationic active substance content

The cationic active content of the obtained C_xAmOMCl was determined by direct two-phase titration according to EN ISO 2871-2,[2] which is suitable for quaternary ammonium salts with a molar mass below 500 g mol^{-1}. Since the tested alkyl[(1*R*,2*S*,5*R*)-(–)-menthoxymethyl]dimethylammonium chlorides at the required concentration levels are completely soluble in water, the titration was performed in a water-chloroform system. The standard solution of sodium dodecyl sulfate (VI) in the presence of the mixed indicator dimidium bromide (CAS: 518-67-2) determined the cationic substances, while the indicator sulfan blue (CAS: 129-17-9) determined the anionic substances.

1.2.4. Electrode preparation

ROX carbon extrudates were ground and sieved to obtain ROX carbon powder with particle size between 50 and 125 µm. The ROX carbon powders were then mixed with conductive additive MWCNTs and binder PTFE, in a mass ratio of 85%: 10%: 5%. Then the mixture was rolled on the hot plate at 383 K to obtain the electrode film with the thickness around 200 µm. The electrodes were activated overnight in vacuum at 333 K.

1.2.5. 2-Electrode capacitor and cyclic voltammetry

Two circular ROX electrodes (10 mm in dimension) were pressed onto titanium (Ti) current collectors coated with carbon ink and heated on a hot plate at 383 K to evaporate the solvent. Then two Ti current collectors with the adhesion of ROX electrode were inserted into customized cell (made of polyether ether ketone (PEEK)), separated with glass microfiber filters (Whatman, 12 mm of dimension). Then, 200 µL of electrolyte was injected through the hole of the PEEK cell and sealed with plastic screws. Cyclic voltammetry was performed in the biologic potentiostat VMP-3 with a scan rate of 1 mV s^{-1} and the voltage window ranging from -1 to 1 V.

1.2.6. 3-Electrode measurement

Based on a 2-electrode capacitor, a silver/silver chloride (Ag/AgCl) reference electrode was inserted through the hole of the PEEK cell. And 300 µL was added to ensure the contact of electrolytes and reference electrode. The potentials of the working and counter electrodes (E_{we} and E_{ce} vs. Ag/AgCl reference) were recorded during the cyclic voltammetry test (scan rate: 1 mV s^{-1}; cell voltage: -1 to 1 V) in the biologic potentiostat VMP-3.

1.2.7. Linear sweep voltammetry (LSV)

In the rubber-covered glass cell, Ti wire was used as the working electrode, with Ti mesh as the counter electrode and Ag/AgCl electrode as the reference electrode to construct a 3-electrode setup for testing electrochemical stability of electrolytes via linear sweep voltammetry. 1 mL of electrolyte (0.1 M) was injected into the glass cell and tested at a scan rate of 10 mV s^{-1} in biologic potentiostat VMP-3.

1.2.8. Zeta potential measurement

10 mg of carbon was added to 150 µL of pure water or electrolyte solutions (0.1 M) for 48 h. Then 2 mL of pure water was added to the mixture of carbon powder and electrolytes. 100 uL of the above suspension solution was obtained and diluted to 2 mL suspension solutions for dynamic laser scattering (DLS) measurement to confirm the zeta potential of carbon powders treated with different electrolyte ions.

1.2.9. Physisorption evaluation and N_2 physisorption

100 mg of ROX powders were treated with 500 µL of 0.1 M electrolytes for 24 h. Then the electrolyte treated carbons were washed with pure water ultrasonically (10 min) and centrifuged 3 times. The washed carbons were treated by lyophilization to obtain ROX samples adsorbed by electrolyte ions. These carbon samples were then degassed overnight at 423 K before physisorption characterization. A Quadrasorb apparatus was used to test nitrogen physisorption at 77 K.

1.2.10. *In situ*-Raman and *in situ*-UV/Vis experiments

DXR SmartRaman Spectrometer (532 nm laser) was used to collect Raman spectra. An *in situ*-cylindrical quartz glass tube was prepared by inserting two ROX carbon electrodes (30 ± 3 mg and 0.5 cm x 1.5 cm for each electrode, pressed on Ti mesh and separated by PVDF separator). After assembly, the cell was filled with ~250 µL of the appropriate electrolyte (50 mM). The high-purity gold was used as the container material for the in-situ cell to reflect and amplify the Raman signal. The duration of each test was set to 5 minutes, with a laser power of 1 mW, and maintained a 25-minute interval between successive tests to reduce the influence of the laser for ion adsorption processes. The IVIUM pocketSTAT 2 potentiostat was connected to the *in situ*-cell for electrochemical testing. Cary 4000 UV-Vis spectrometer (Agilent) was used to detect UV/Vis absorbance. An *in situ*-cuvette cell was prepared by inserting two ROX electrodes (30 ± 3 mg and 0.5 cm x 1.5 cm for each electrode, pressed on Ti mesh and separated by PVDF separator) in the cuvette (pathlength 10 mm, chamber volume 3.5 mL). After assembly, the cell was filled with 3 mL of the appropriate electrolyte (1 mM). After 3-day rest, the cell was connected to the biologic potentiostat VMP-3 for electrochemical chronoamperomtery testing and measured once every 5 min. For one cycle, the bias of 0.5 V for 0.5 h was applied to the cell, followed by 1 V for 0.5 h, 0.5 V for 0.5 h, 0 V for 0.5 h, -0.5 V for 0.5 h, -1 V for 0.5 h, -0.5 V for 0.5 h, 0 V for 0.5 h.

1.2.11. 2-, 3-, and 4-Terminal device construction and resistance switching measurement.

For a 4-terminal device, ROX carbon electrodes (two of 30 ± 3 mg and 1.5 cm x 1.5 cm electrodes as M-Cap, and two of 1.2 ± 0.3 mg and 0.2 cm x 0.2 cm electrodes as D-Cap) were pressed onto the Ti mesh current collector. Four electrodes were sealed in a plastic bag in 200 µL of electrolyte (200 µL per 60 mg of two large electrodes) with hydrophilic PVDF membranes as separator. The devices were tested after 5-h rest. M-Cap was tested by chronoamperometry in one potentiostat channel and D-Cap was tested by EIS (frequencies from 200 kHz to 100 Hz at an amplitude alternative voltage of 10 mV) in another potentiostat channel.

A pulse time was followed by the resting time (no bias voltage is applied to M-Cap during resting time, and only the open circuit voltage is recorded) within 40 s to the M-Cap. 10 times of 1 V-pulses were applied to the M-Cap followed by 10 times of 0 V-pulses with different pulse time (t_p = 20 s) and the resistances of D-Cap were tested by electrochemical impedance spectroscopy (EIS) during the resting time (t_r = 20 s). The pulse time and resting time were controlled by a potentiostat channel in Biologic VMP-3 potentiostat. EIS measurement was conducted with frequencies from 200 kHz to 100 Hz under an amplitude of 10 mV in Bio-Logic VMP-3 potentiostat.

3- and 2-terminal devices were tested based on 4-terminal devices. For a 3-terminal device, two small electrodes (two of 1.2 ± 0.3 mg and 0.2 cm x 0.2 cm electrodes) were connected as drain (D) electrode, two large electrodes (two of 30 ± 3 mg and 1.5 cm x 1.5 cm electrodes) were used as gate (G) and source (S) electrodes, respectively. For a 2-terminal device, two large electrodes (two of 30 ± 3 mg and 1.5 cm x 1.5 cm electrodes) were connected to a potentiostat channel in Biologic VMP-3 potentiostat, and 10 times of 1 V-pulses were applied to these two electrodes, followed by EIS test through the same channel during the rest period. EIS experiment performed impedance measurements into potentiostatic mode in applying a sinus around a DC potential that was set to the fixed value (i.e., the value of recorded cell voltages after each 1 V-pulse applied to the cell); frequencies from 200 kHz to 100 kHz at an amplitude alternative voltage of 10 mV. The ratio of electrolytes and electrodes was 200 µL of electrolytes per 60 mg of two large electrodes.

Gel electrolytes were prepared by adding equal volumes of 0.2 M C_{12}AmOMCl electrolyte and polyvinyl alcohol (PVA) gel. PVA gel was prepared by adding 0.5 g PVA (Merck; molecular weight 145 000 g mol^{-1}) in 7 mL water (stirring at 85 °C).

1.3. General methods for synthesis and storage conditions

1.3.1. Synthesis of alkyl[(1*R*,2*S*,5*R*)-(–)-menthoxymethyl]dimethylammonium chlorides (C_xAmOMCl)

Chloromethyl (1*R*,2*S*,5*R*)-(–)-menthyl ether (OMCl) was synthesized according to the following procedure. A suspension of paraformaldehyde and (1*R*,2*S*,5*R*)-(–)-menthol in toluene was prepared, and HCl gas was passed through the reaction mixture. This method, as reported by Pernak and Feder-Kubis,[3] was carried out under isothermal conditions no warmer than 5 °C. The obtained chiral ether (OMCl) was purified using vacuum distillation to obtain a pure, transparent, and colorless liquid with a yield above 98.5%. OMCl is an excellent quaternary agent.

Prior to its use in the quaternization reaction, OMCl—as well as purchased commercial amines (*N*,*N*-dimethylethylamine, *N*,*N*-dimethylhexylamine, and *N*,*N*-dimethyldodecylamine)—were purified via vacuum distillation to ensure its purity and reliability for further reactions.

Alkyl[(1*R*,2*S*,5*R*)-(–)-menthoxymethyl]dimethylammonium chlorides (C_xAmOMCl) were synthesized according to a method previously described by our group procedure.[3] Briefly, chloromethyl (1*R*,2*S*,5*R*)-(–)-menthyl ether (OMCl) (0.062 mol) was added dropwise to a round-bottomed flask containing a vigorously stirred mixture of 30 mL dry *n*-hexane and freshly distilled appropriate aliphatic amine (0.06 mol). The quaternization process was almost instantaneous, and the product precipitated within several minutes of combining the reactants. The progress of the reaction was monitored using ascending thin layer chromatography on silica gel G (Merck 1.05570.0001) and visualized under UV light. The reaction mixture was stirred at room temperature for the next 90 min. Next, the phases were separated, and the crude product (3) was washed with dry *n*-hexane (3 x 30 mL). Volatiles were removed under reduced pressure (0.3 mmHg) at 60 °C overnight. The resulting salt was further dried under vacuum (0.3 mmHg) to obtain an analytically pure product.

Prior to any measurements, the alkyl[(1*R*,2*S*,5*R*)-(–)-menthoxymethyl]dimethylammonium chlorides (C_xAmOMCl) were dried under vacuum for 48 h. The structure and purity of the synthesized C_xAmOMCl were confirmed via spectral analysis.

Ethyl[(1*R*,2*S*,5*R*)-(–)-menthoxymethyl]dimethylammonium chloride (C_2AmOMCl)

^{1}H-NMR (300 MHz, D_2O): δ [ppm] = 4.58 (s, 2H, 11-H), 3.56 (td, *J*=10.7, 4.3 Hz, 1H, 1-H), 3.29 (q, *J*=7.3 Hz, 2H, 14-H_2), 2.91 (s, 6H, 12-H_3, 13-H_3), 2.07–1.97 (m, 2H, 6b-H and 7-H), 1.65–1.65 (m, 2H, 3b-H and 4b-H), 1.35–1.19 (m, 5H, 5-H and 2-H, 15-H_3), 1.02–0.875 (m, 2H, 3a-H and 6a-H), 0.855-0.82 (m, 6H, 8-H_3 and 10-H_3), 0.80–0.74 (m, 1H, 4a-H), 0.72 (d, *J*=6.9 Hz, 3H, 9-H_3). **^{13}C{^{1}H}-NMR** (150 MHz, D_2O): δ [ppm] = 87.955 (t, $J^{1,2}_{C,N}$ = 4.5 Hz, $J^{2,1'}_{C,N}$ = 3.0 Hz, 11-CH_2), 82.83 (1-CH), 56.84 (br, 14-CH_2), 48.04 (2-CH), 46.815 (t, $J^{1,2}_{C,N}$ = 4.5 Hz, $J^{2,1'}_{C,N}$ = 3.0 Hz, 12-CH_3), 46.785 (t, $J^{1,2}_{C,N}$ = 3.0 Hz, $J^{2,1'}_{C,N}$ = 4.5 Hz, 13-CH_3), 40.21 (6-CH_2), 33.63 (4-CH_2), 30.90 (5-CH), 25.25 (7-CH), 22.38 (3-CH_2), 21.43 (10-CH_3), 20.37 (8-CH_3), 15.11 (9-CH_3), 7.18 (15-CH_3).

Hexyl[(1*R*,2*S*,5*R*)-(–)-menthoxymethyl]dimethylammonium chloride (C_6AmOMCl)

^{1}H-NMR (600 MHz, D_2O): δ [ppm] = 4.565 (d, *J*=6.0 Hz, 1H, 11b-H, system AB), 4.535 (d, *J*=6.0 Hz, 1H, 11a-H, system AB), 3.50 (td, *J*=12.0, 6.0 Hz, 1H, 1-H), 3.17-3.14 (m, 2H, 14-H_2), 2.91 (s, 6H, 12-H_3 and 13-H_3), 2.05–1.97 (m, 2H, 6b-H and 7-H), 1.63–1.53 (m, 4H, 15-H_2, 3b-H and 4b-H), 1.37–1.17 (m, 8H, 5-H, 2-H, 16-H_2, 17-H_2 and 18-H_2), 0.95-0.84 (m, 2H, 3a-H and 6a-H), 0.825 (d, *J*=6.0 Hz, 3H, 8-CH_3), 0.815 (d, *J*=6.0 Hz, 3H, 10-CH_3), 0.77 (t, *J*= 6.0 Hz, 3H, 19-H_3), 0.75–0.72 (m, 1H, 4a-H), 0.705 (d, *J*=6.0 Hz, 3H, 9-CH_3). **^{13}C{^{1}H}-NMR** (150 MHz, D_2O): δ [ppm] = 87.30 (br, 11-CH_2), 81.76 (1-CH), 61.07 (br, 14-CH_2), 48.07 (2-CH), 47.76 (br, 12-CH_3), 47.54 (br, 13-CH_3), 40.09 (6-CH_2), 33.80 (4-CH_2), 30.80 (5-CH), 30.36 (17-CH_2), 25.31 (7-CH), 25.24 (16-CH_2), 22.42 (3-CH_2), 21.77 (18-CH_2), 21.51 (10-CH_3), 21.49 (15-CH_2), 20.55 (8-CH_3), 15.27 (9-CH_3), 13.34 (19-CH_3).

Dodecyl[(1*R*,2*S*,5*R*)-(–)-menthoxymethyl]dimethylammonium chloride (C_{12}AmOMCl)

^{1}H-NMR (600 MHz, D_2O): δ [ppm] = 4.715 (d, *J*=6.0 Hz, 1H, 11b-H, system AB), 4.665 (d, *J*= 6.0 Hz, 1H, 11a-H, system AB), 3.605 (td, *J*=12.0, 6.0 Hz, 1H, 1-H), 3.26-3.20 (m, 2H, 14-H_2), 3.082-307 (m, 6H, 12-H_3 and 13-H_3), 2.26-2.24 (m, 1H, 6b-H), 2.18-2.10 (m, 1H, 7-H), 1.73–1.67 (m, 3H, 4b-H, 15-H_2), 1.60–1.58 (m, 1H, 3b-H), 1.50–1.47 (m, 1H, 5-H), 1.39–1.26 (m, 19H, 2-H and 9xCH_2:16-H_2÷24-H_2), 1.12-1.02 (m, 1H, 3a-H), 0.95-0.73 (m, 14H, 10-CH_3, 8-CH_3, 6a-H, 25-H_3, 9-CH_3 and 4a-H). **^{13}C{^{1}H}-NMR** (150 MHz, D_2O): δ [ppm] = 86.25 (11-CH_2), 80.37 (1-CH), 60.62 (14-CH_2), 48.53 (2-CH), 48.45 (12-CH_3), 48.39 (13-CH_3), 40.24 (6-CH_2), 34.46 (4-CH_2), 32.10 (CH_2), 30.85 (5-CH), 30.09 (CH_2), 30.04 (CH_2), 29.99 (CH_2), 29.63 (CH_2), 29.59 (CH_2), 28.76 (CH_2), 25.93 (CH_2), 25.72 (7-CH), 22.71 (3-CH_2, CH_2), 21.97 (10-CH_3), 21.71 (CH_2), 21.12 (8-CH_3), 15.75 (9-CH_3), 13.87 (25-CH_3).

1.3.2. Synthesis of 2-hydroxyethyl[(1*R*,2*S*,5*R*)-(–)-menthoxymethyl] dimethylammonium chloride ($C_{2\text{-}OH}$AmOMCl)

The synthesis procedure for the newly developed ammonium salt ($C_{2\text{-}OH}$AmOMCl) is consistent with that described above for the preparation of C_xAmOMCl. To ensure the proper preparation of reagents, *N*,*N*-dimethyl-2-hydroxyethylamine—with a boiling point of approximately 134–135°C—must be purified prior to each use.

First, we synthesized a quaternary reagent (OMCl). That reagent then underwent a quaternization reaction with *N*,*N*-dimethyl-2-hydroxyethylamine. The resulting product, $C_{2\text{-}OH}$AmOMCl, formed almost immediately as a clear powder. Upon crystallization in an ethyl acetate/acetone system, a crystalline compound with irregular, short needles were observed. The melting point of the salt obtained, measured to range from 113.8–114.1 °C, indicates its status as a quaternary ammonium salt.

^{1}H-NMR (600 MHz, D_2O): δ [ppm] = 4.645 (d, *J*=6.0 Hz, 1H, 11b-H, system AB), 4.73 (d, *J*= 7.4 Hz, 1H, 11a-H, system AB), 3.39-3.92 (m, 2H, 15-H_2), 3.545 (td, *J*=12.0, 6.0 Hz, 1 H, 1-H), 3.35-3.37 (m, 2H, 14-H_2), 2.99 (s, 6H, 12-H_3 and 13-H_3), 2.02–1.95 (m, 2H, 6b-H and 7-H), 1.59–1.53 (m, 2H, 3b-H and 4b-H), 1.34–1.23 (m, 2H, 2-H and 5-H), 0.95–0.88 (m, 2 H, 3a-H and 6a-H), 0.815 (d, *J*=6.0 Hz, 3H, 8-CH_3), 0.795 (d, *J*=6.0 Hz, 3 H, 10-CH_3), 0.78-0.70 (m, 1H, 4a-H), 0.685 (d, *J*=6.0 Hz, 3 H, 9-CH_3). **^{13}C{^{1}H}-NMR** (150 MHz, D_2O): δ [ppm] = 89.59 (11-CH_2), 82.93 (1-CH), 62.35 (14-CH_2), 55.25 (15-CH_2), 48.17 (br, 12-CH_3, 13-CH_3), 48.05 (2-CH), 40.20 (6-CH_2), 33.62 (4-CH_2), 30.91 (5-CH), 25.26 (7-CH), 22.37 (3-CH_2), 21.42 (10-CH_3), 20.39 (8-CH_3), 15.11 (9-CH_3).

2. Results and Discussion

Details for alkyl[(1R,2S,5R)-(–)-menthoxymethyl]dimethylammonium chlorides (C_xAmOMCl) synthesis

In the first step chloromethyl (1*R*,2*S*,5*R*)-(–)-menthyl ether (OMCl) was prepared via chloromethylation of (1*R*,2*S*,5*R*)-(–)-menthol according to a previously described procedure.[3] The ether-based terpene derivative OMCl is an attractive reagent for quaternization. Notably, the process of preparing this quaternary agent should be carried out under strictly anhydrous conditions; otherwise, the resultant ether will readily undergo hydrolysis.[3] In the next step, alkyl[(1*R*,2*S*,5*R*)-(–)-menthoxymethyl]dimethylammonium chlorides (C_xAmOMCl) were synthesized in a Menschutkin reaction. Quaternization was performed under anhydrous conditions using distilled appropriate *N*,*N*-alkyldimethylamine and freshly distilled OMCl. The Menschutkin reaction occurred immediately at room temperature with anhydrous hexane as a solvent and produced high yields of C_xAmOMCl (ranging from 97.9 to 99.9%)

Further discussion of the interaction mechanism

With the goal of analyzing the interactions between various ionic compounds and porous carbons, we synthesized 2-hydroxyethyl[(1*R*,2*S*,5*R*)-(–)-menthoxymethyl]dimethylammonium chloride ($C_{2\text{-}OH}$AmOMCl) (Figure S26). This novel salt is a derivative of ChCl wherein the methyl group attached to the nitrogen atom is replaced by a (1*R*,2*S*,5*R*)-(–)-menthoxymethyl group (Figure S26). The crude product, $C_{2\text{-}OH}$AmOMCl, was subjected to crystallization to obtain a final yield of 99.2%. We refer the reader to the NMR spectra and mass spectrum shown in Figure S27-S33. The melting points of $C_{2\text{-}OH}$AmOMCl range from 113.8 to 114.1 °C.

We evaluated the differences in physisorption between $C_{2\text{-}OH}$AmOMCl and C_2AmOMCl using zeta potentials and *in situ*-Raman experiments. The zeta potential of $C_{2\text{-}OH}$AmOMCl-treated ROX carbon was similar to that of ChCl (i.e., roughly -21.6 mV). This value is quite a bit lower than the value for C_2AmOMCl (-9.5 mV), which indicated a weaker interaction between $C_{2\text{-}OH}$AmOM cations and ROX compared with C_2AmOM cations and ROX (Figure S34). Furthermore, a strong Raman signal for $C_{2\text{-}OH}$AmOMCl persisted after 13 h of monitoring encompassing physisorption, electroadsorption and electrodesorption; the concentration decreased from 50 mM to approximately 40 mM (Figure S36) (see the calibration curve for $C_{2\text{-}OH}$AmOMCl shown in Figure S35). However, for C_2AmOMCl—which lacks an OH group compared with $C_{2\text{-}OH}$AmOMCl (Figure S36a)— the concentration decreased below 20 mM after 13 h. The different

adsorption kinetics of C_2AmOMCl and $C_{2\text{-}OH}$AmOMCl additionally indicated that the interacting group of bioactive cations for the exchange of C_2AmOMCl to C_{12}AmOMCl was the alkyl chain rather than the monoterpene moiety (Figure S36b). The presence of the OH group in $C_{2\text{-}OH}$AmOMCl weakened the adsorption effect of molecules in porous carbons with hydrophobic surfaces.

Further discussion on various carbon materials

On this account, we investigated YP-50F (produced by Kuraray) and AC-CS (produced by Jacobi) to verify this interaction theory (Figure S41). We found weak physisorption for choline cations in AC-CS and YP-50F carbons; these carbons exhibited strong adsorption for C_{12}AmOM cations, similar to ROX carbon. The more rapid adsorption kinetics associated with C_{12}AmOM cations within the pores of AC-CS and YP-50F resulted from the higher surface areas and pore volumes of AC-CS (1394 m^2 g^{-1} and 0.631 cm^3 g^{-1}) and YP-50F (1431 m^2 g^{-1} and 0.750 cm^3 g^{-1})[4] compared with ROX carbon (1182 m^2 g^{-1} and 0.515 cm^3 g^{-1}).

Further discussion on memory time of 2-, 3-, and 4-terminal memristors

We evaluated the memory time of 2-, 3-, and 4-terminal devices (Figure S49). After 10 times of 1 V-pulses, we noted a 49%, 42%, and 75% increase in the resistance for 2-, 3-, and 4-terminal memristors, respectively. The 4-terminal device showed the highest increase in resistance, which in turn resulted in rapid attenuation after the pulses stopped. Compared to 4-terminal devices, 2-terminal devices showed a sharper drop in resistance after the pulse, because of the ions adsorbed by the electrode desorbed back into the bulk electrolytes. The 3-terminal device experienced a 42% increase, resulting in a slow decay compared to the 2- and 4-terminal devices. Moreover, we observed much higher increase in the resistance for these memristors after 100 times of 1 V-pulses, 174%, 100%, and 293% for 2-, 3-, and 4-terminal memristors, respectively. These increased values of resistance decreased to 56%, 38%, and 116% after 4000 s of rest.

3. Supplementary Figures and Tables

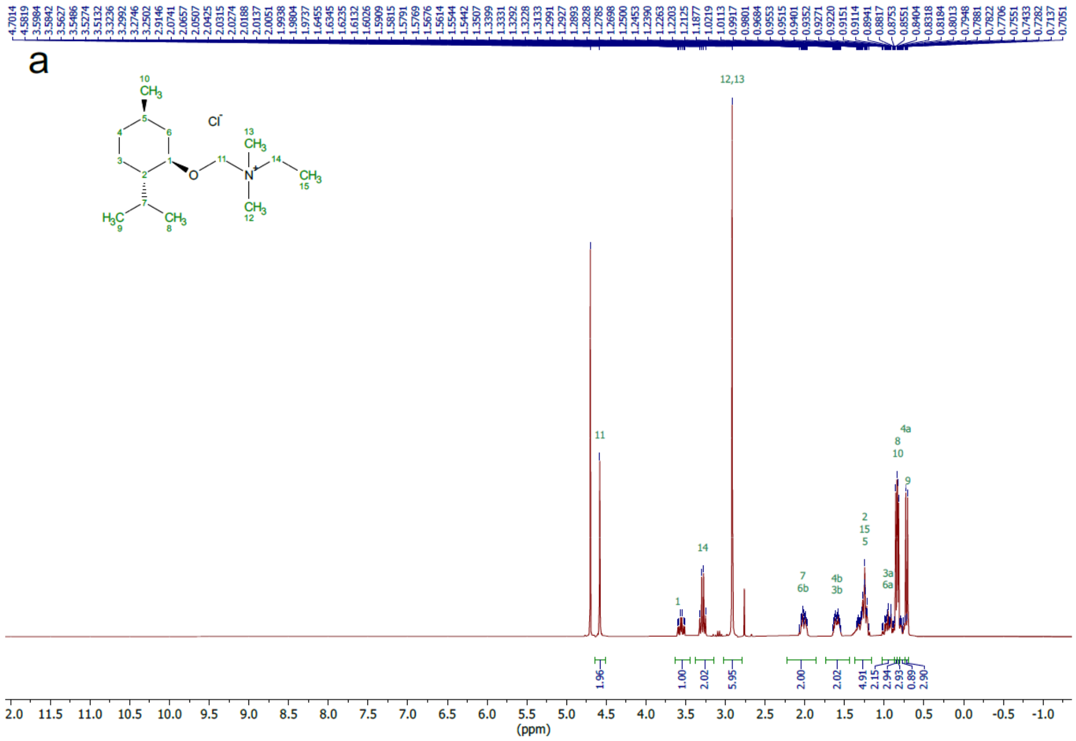

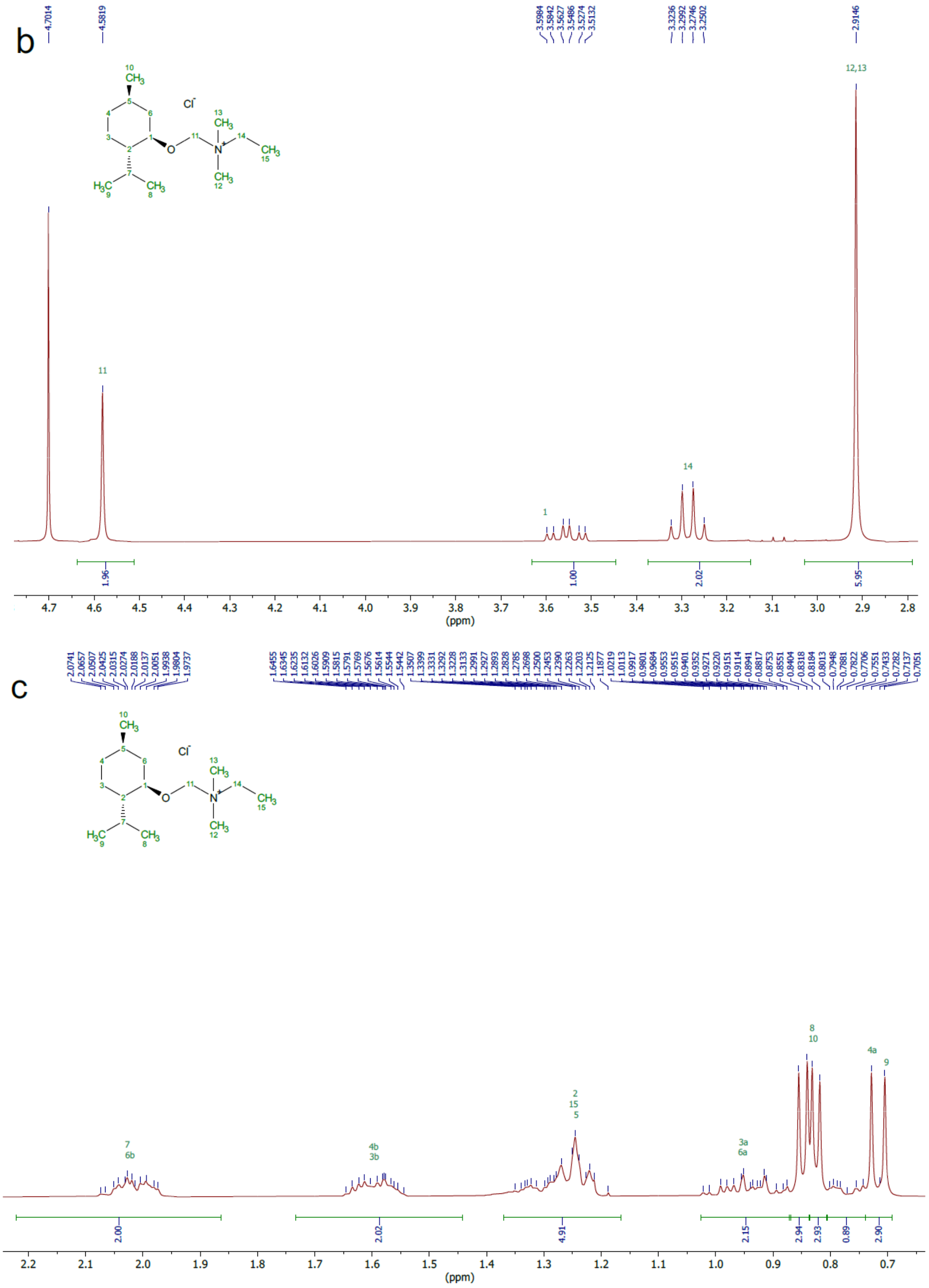

Figure S1. ^{1}H NMR (300 MHz) spectra for C_2AmOMCl in D_2O+TMS. a) region -1.0-12.0 ppm, b) region 2.8-4.7 ppm, and c) 0.7-2.2 ppm.

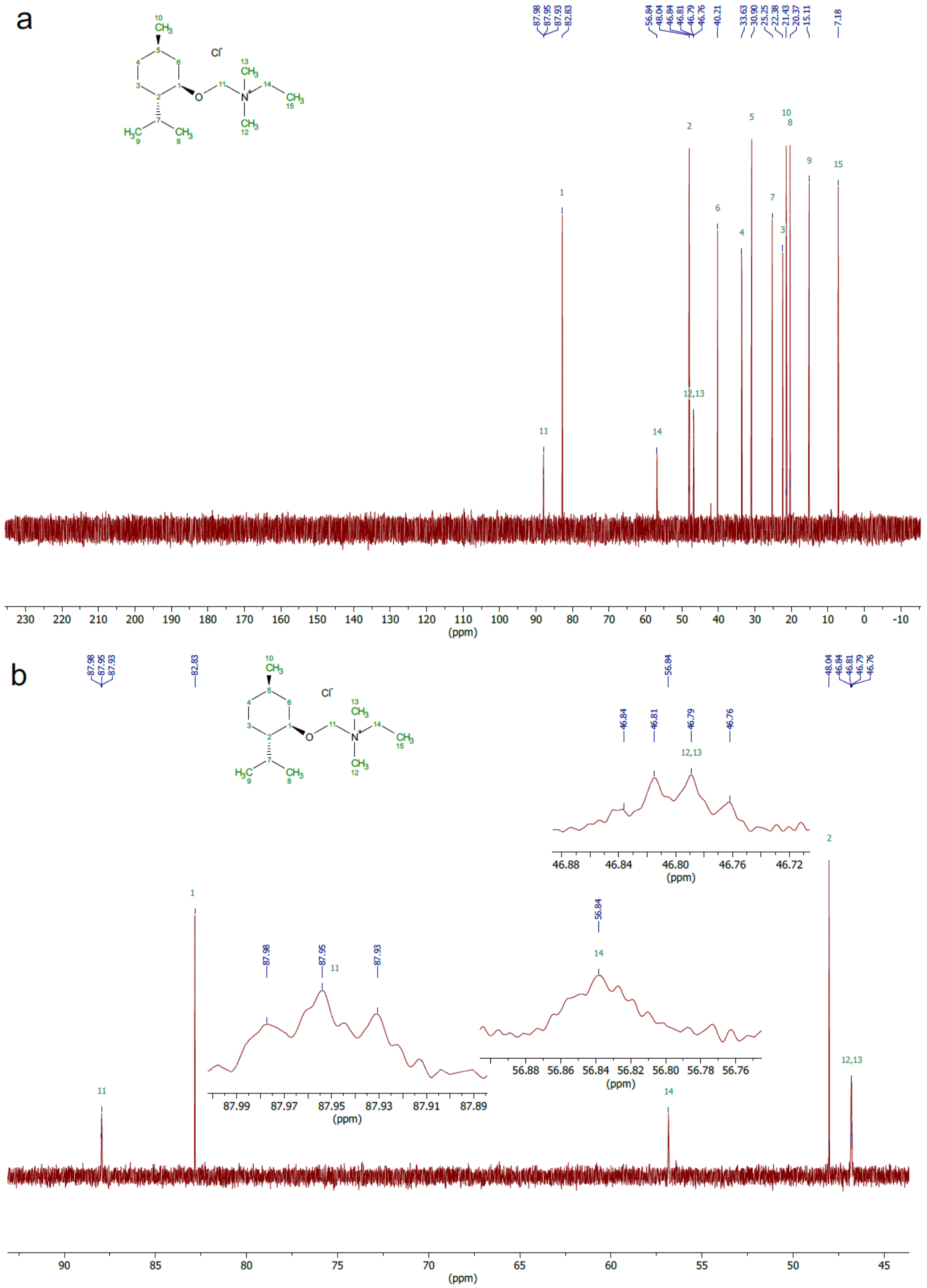

Figure S2. $^{13}C\{^{1}H\}$ NMR (151 MHz) spectra for C_2AmOMCl in D_2O+TMS. a) region -10-230 ppm, and b) region 45-90 ppm.

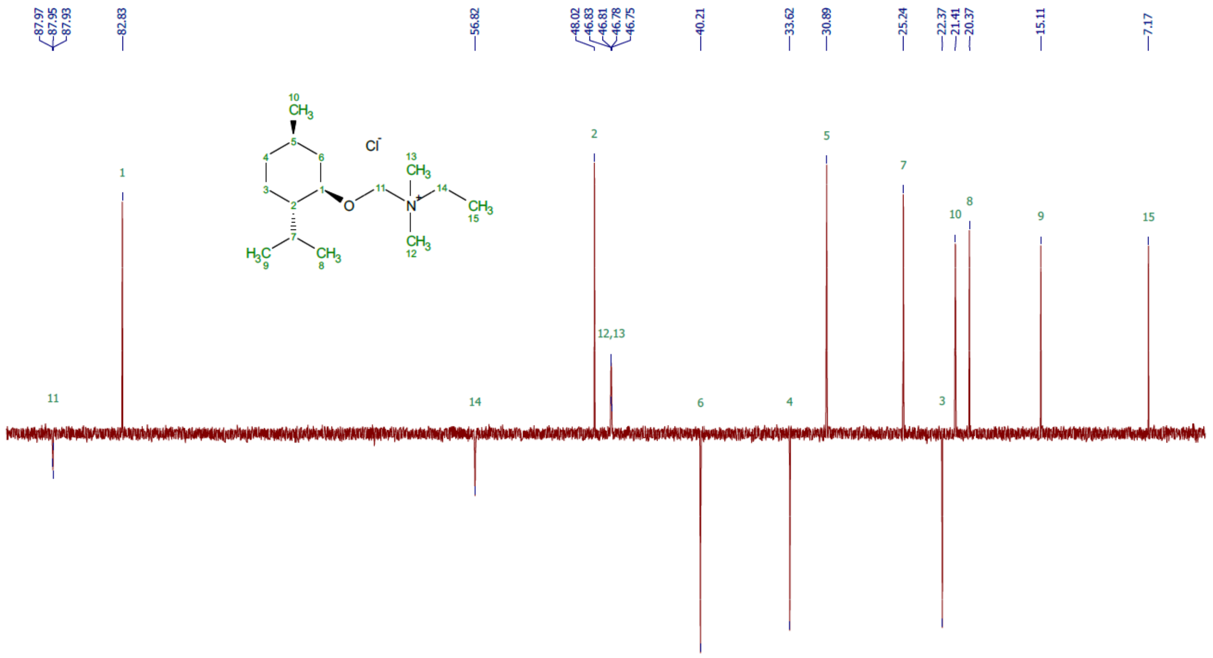

Figure S3. ^{13}C DEPT-135° NMR experiments (151 MHz, D_2O) for C_2AmOMCl.

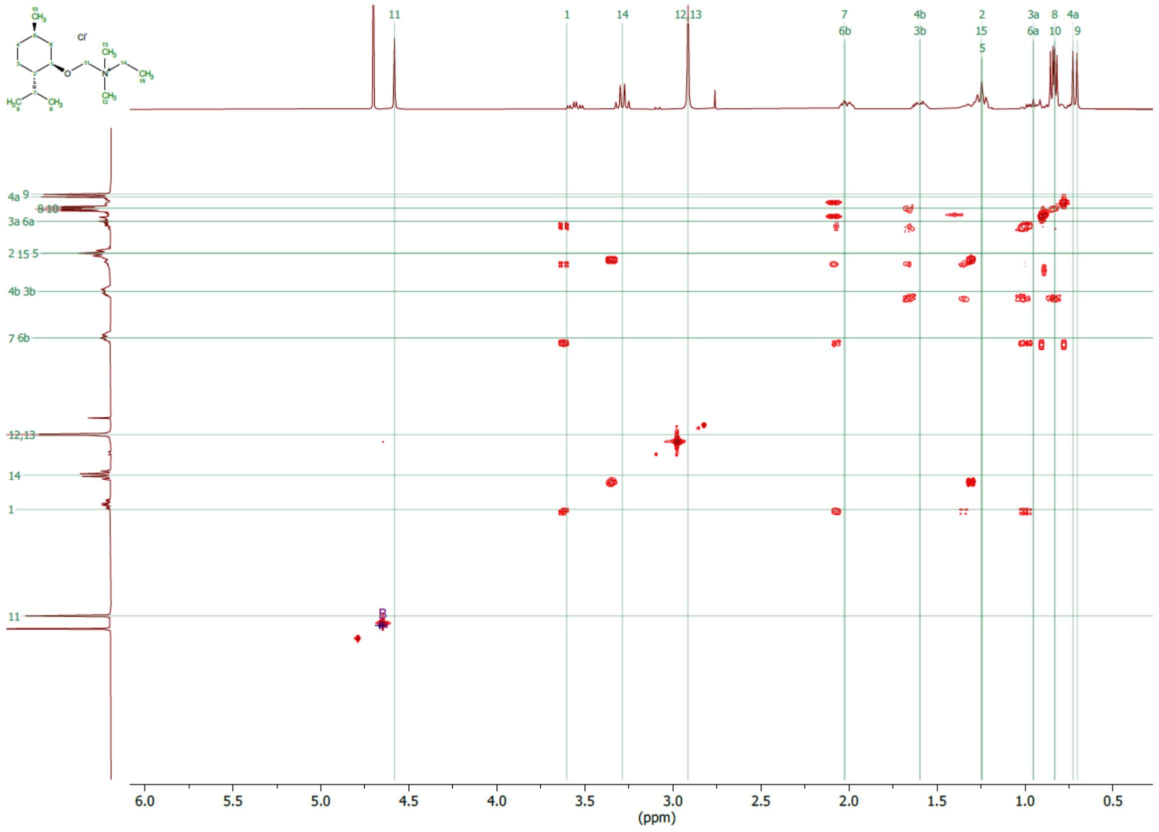

Figure S4. ^{1}H,^{1}H COSY experiment (600 MHz) for C_2AmOMCl in D_2O.

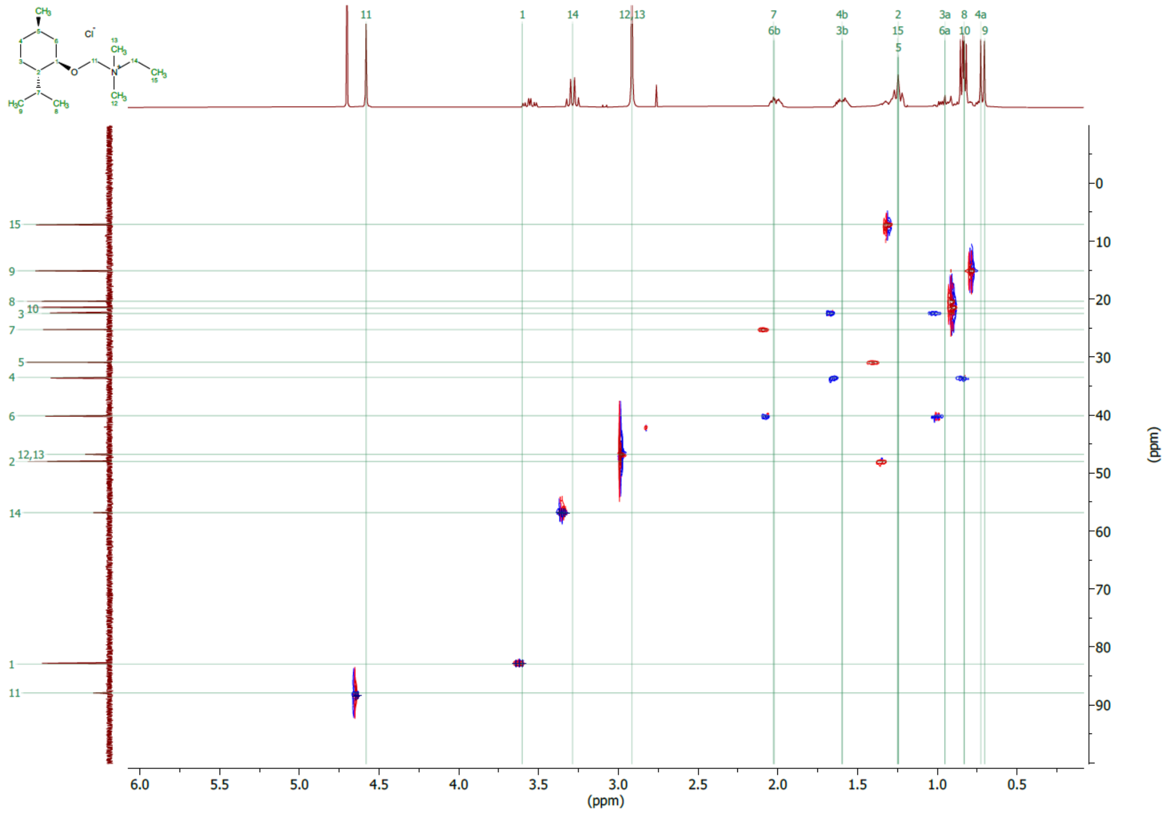

Figure S5. ^{1}H,^{13}C multiplicity-resolved HSQC (CH_2-blue, CH/CH_3-red) experiments for C_2AmOMCl in D_2O.

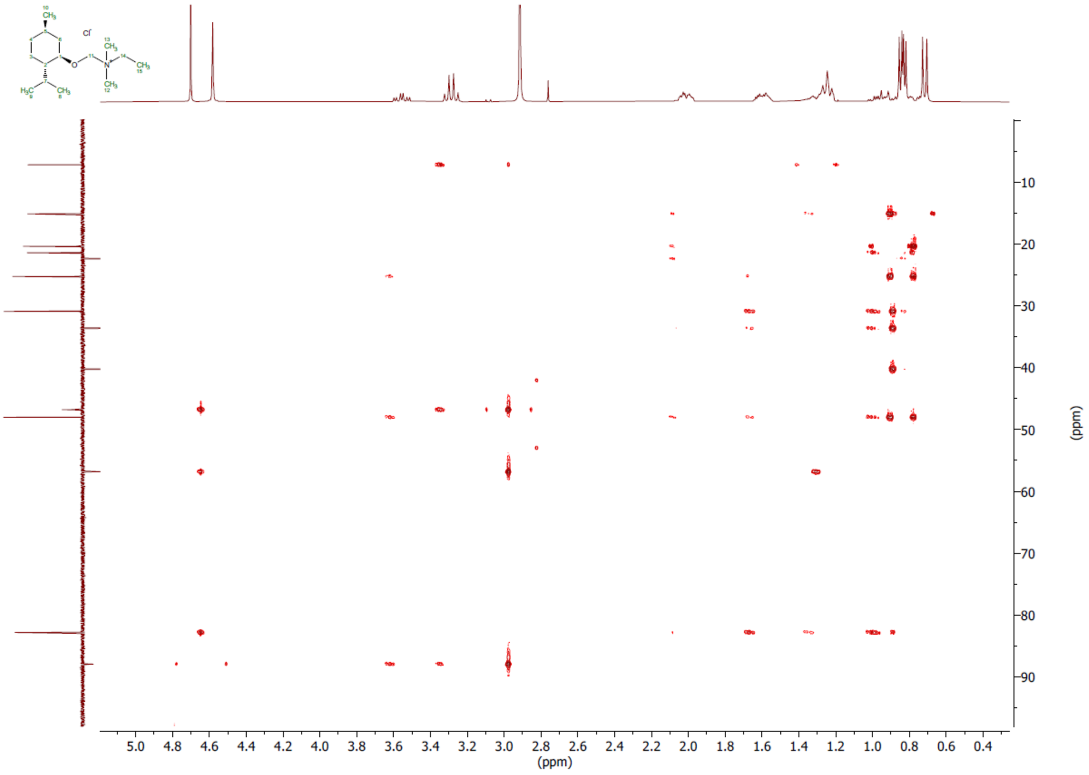

Figure S6. ^{1}H,^{13}C multiplicity-resolved HMBC experiments for C_2AmOMCl in D_2O.

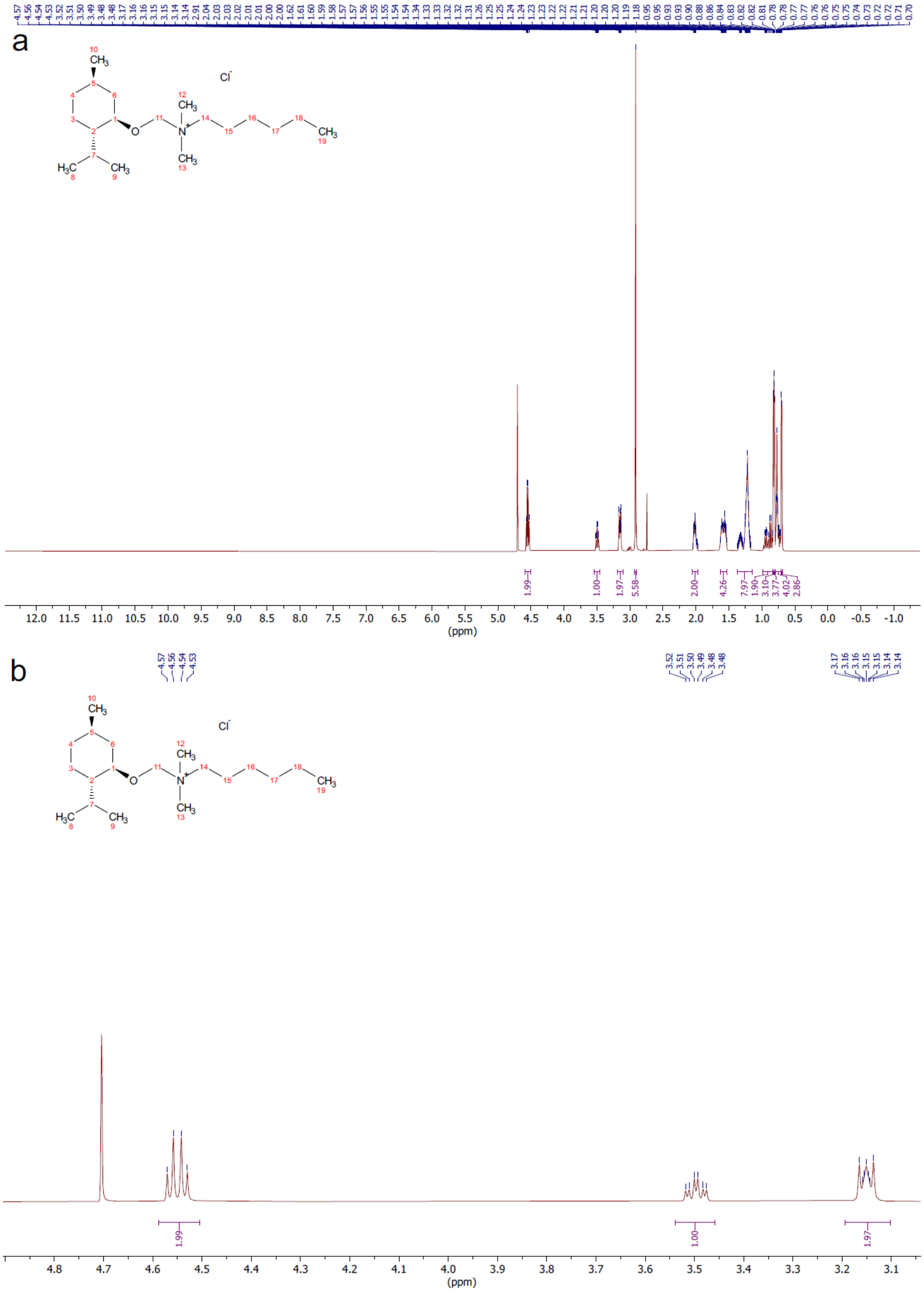
a
b
Cl⁻
(ppm)

c

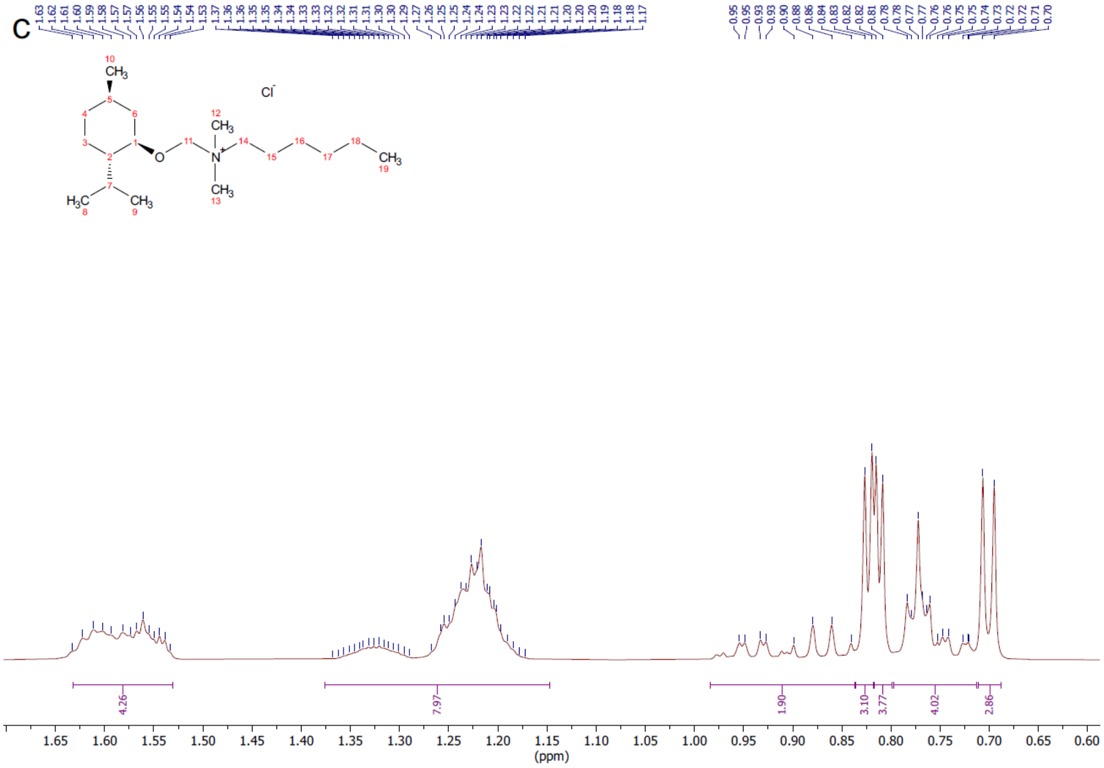

Figure S7. ^{1}H NMR (600 MHz) spectra for $C_6AmOMCl$ in D_2O+TMS. a) region -1.0-12.0 ppm, b) region 3.1-4.8 ppm, and c) 0.65-1.65 ppm.

a

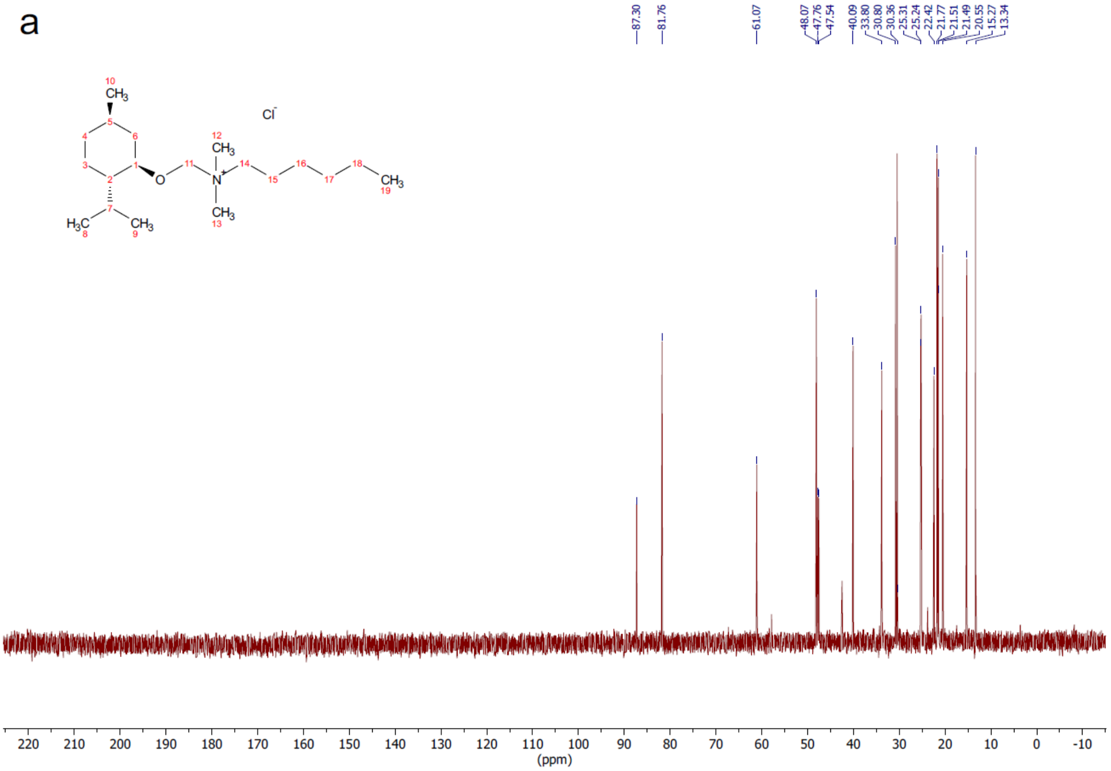

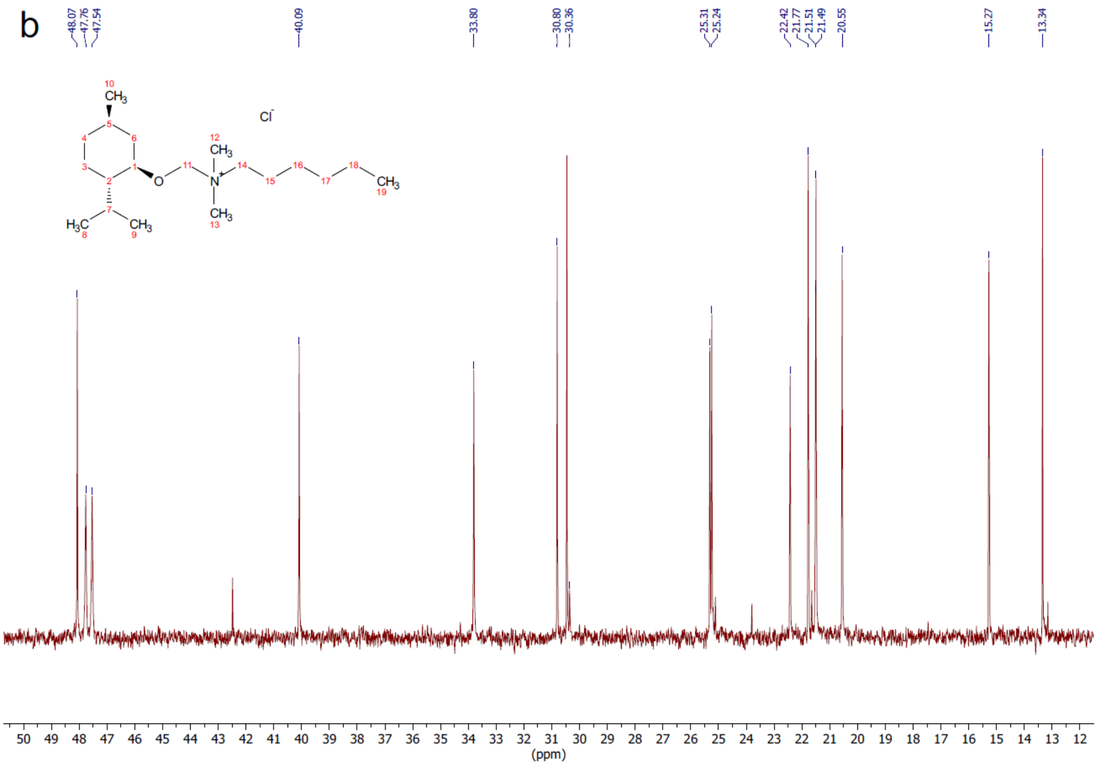

Figure S8. $^{13}C\{^{1}H\}$ NMR (151 MHz) spectra for C_6AmOMCl in D_2O+TMS. a) region -10-220 ppm, and b) region 12-50 ppm.

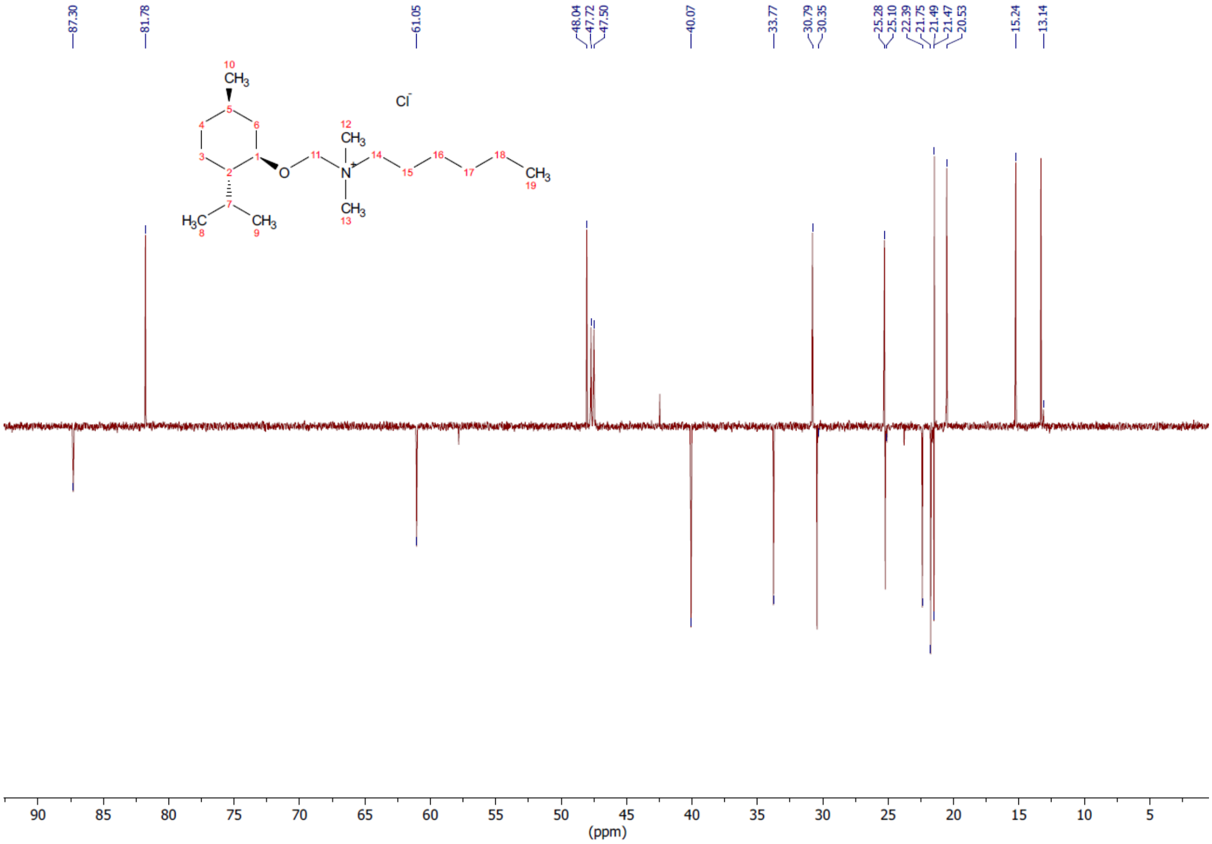

Figure S9. ^{13}C DEPT-135° NMR experiments (151 MHz, D_2O) for C_6AmOMCl.

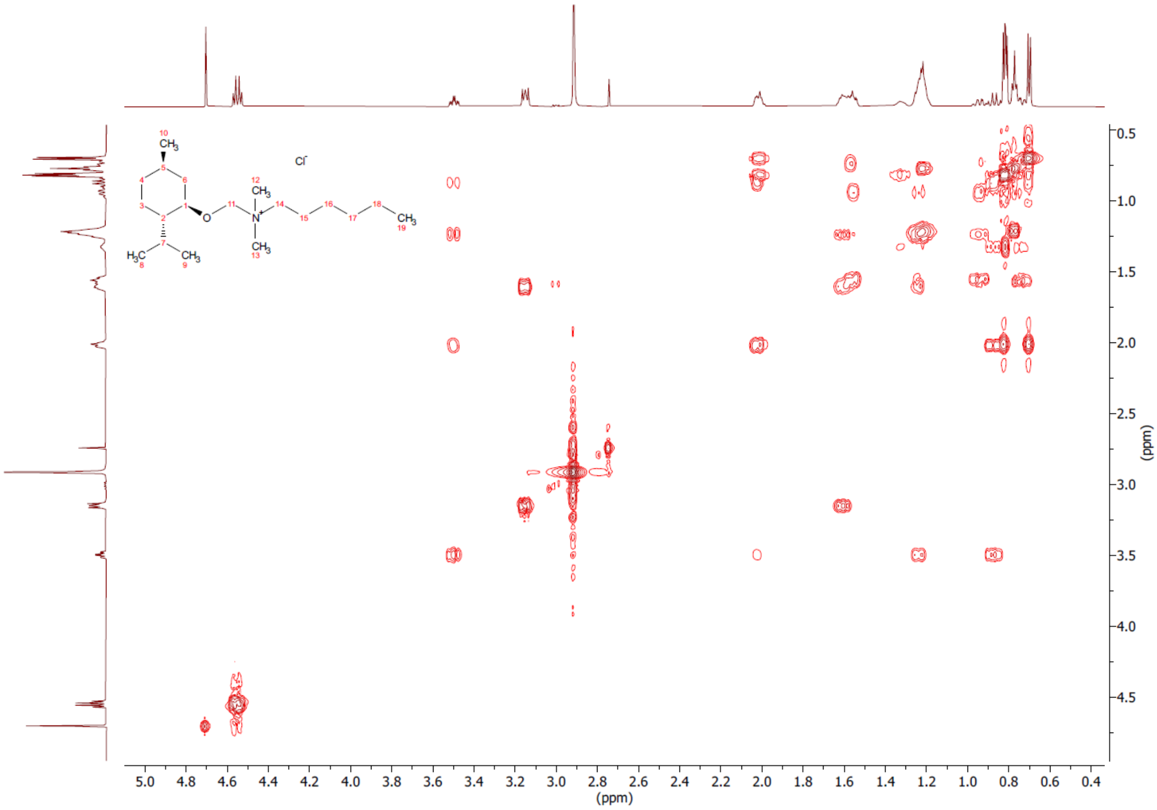

Figure S10. ^{1}H,^{1}H COSY experiment (600 MHz) for C_6AmOMCl in D_2O.

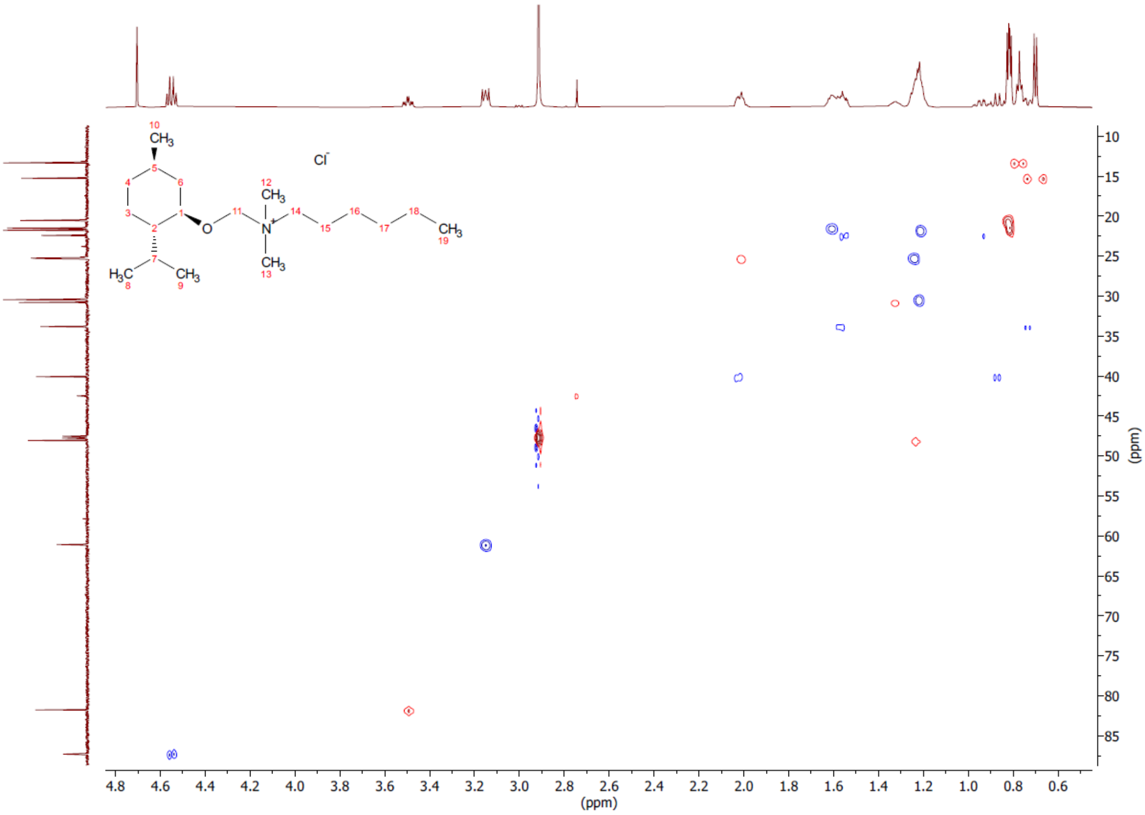

Figure S11. ^{1}H,^{13}C multiplicity-resolved HSQC (CH_2-blue, CH/CH_3-red) experiments for C_6AmOMCl in D_2O.

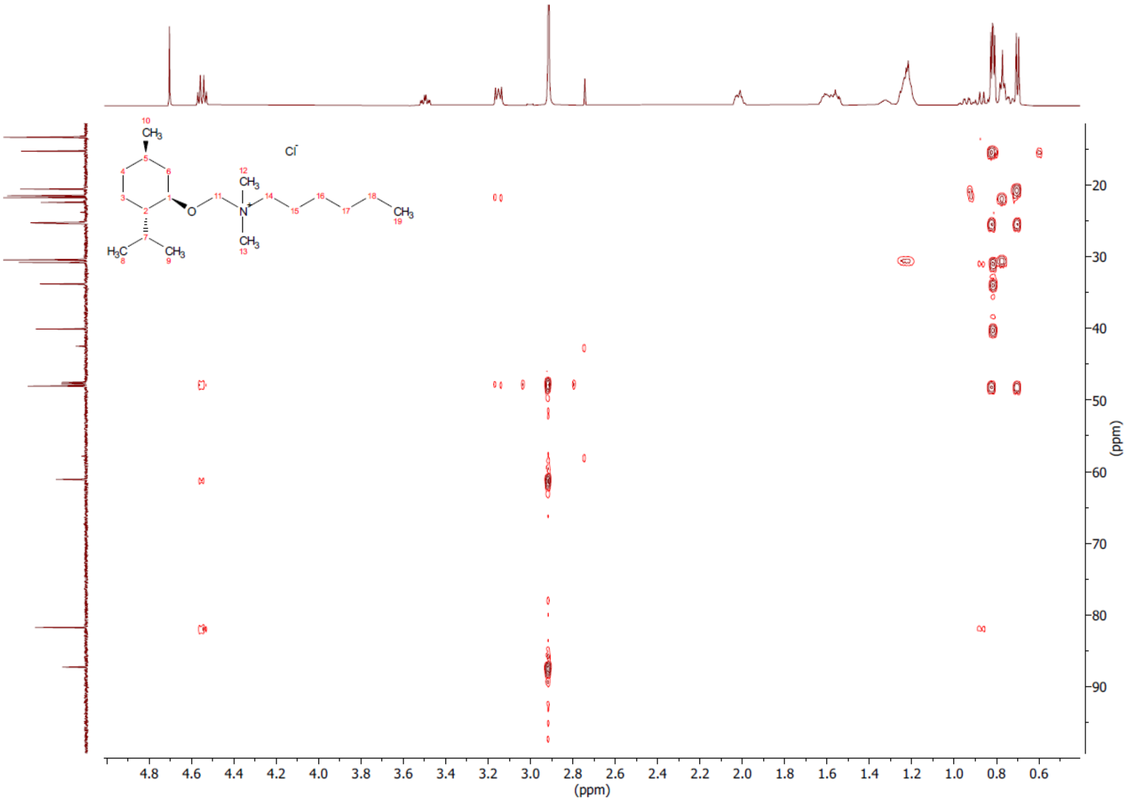

Figure S12. $^{1}H,^{13}C$ multiplicity-resolved HMBC experiments for C_6AmOMCl in D_2O.

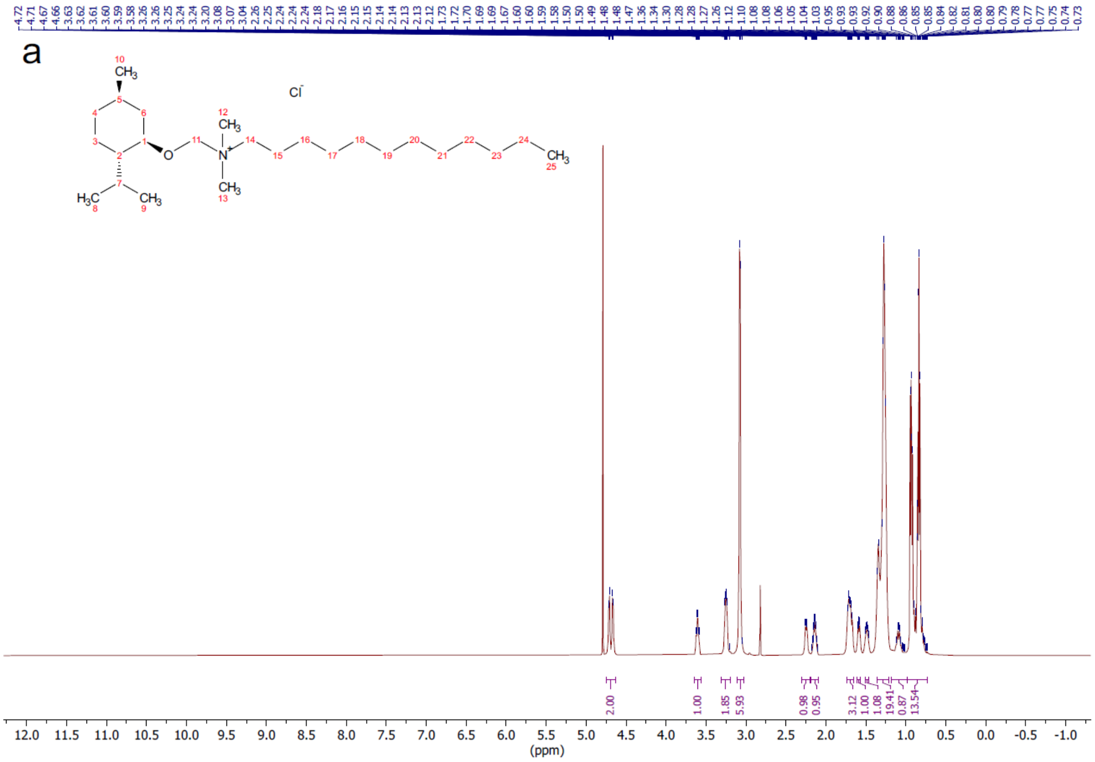

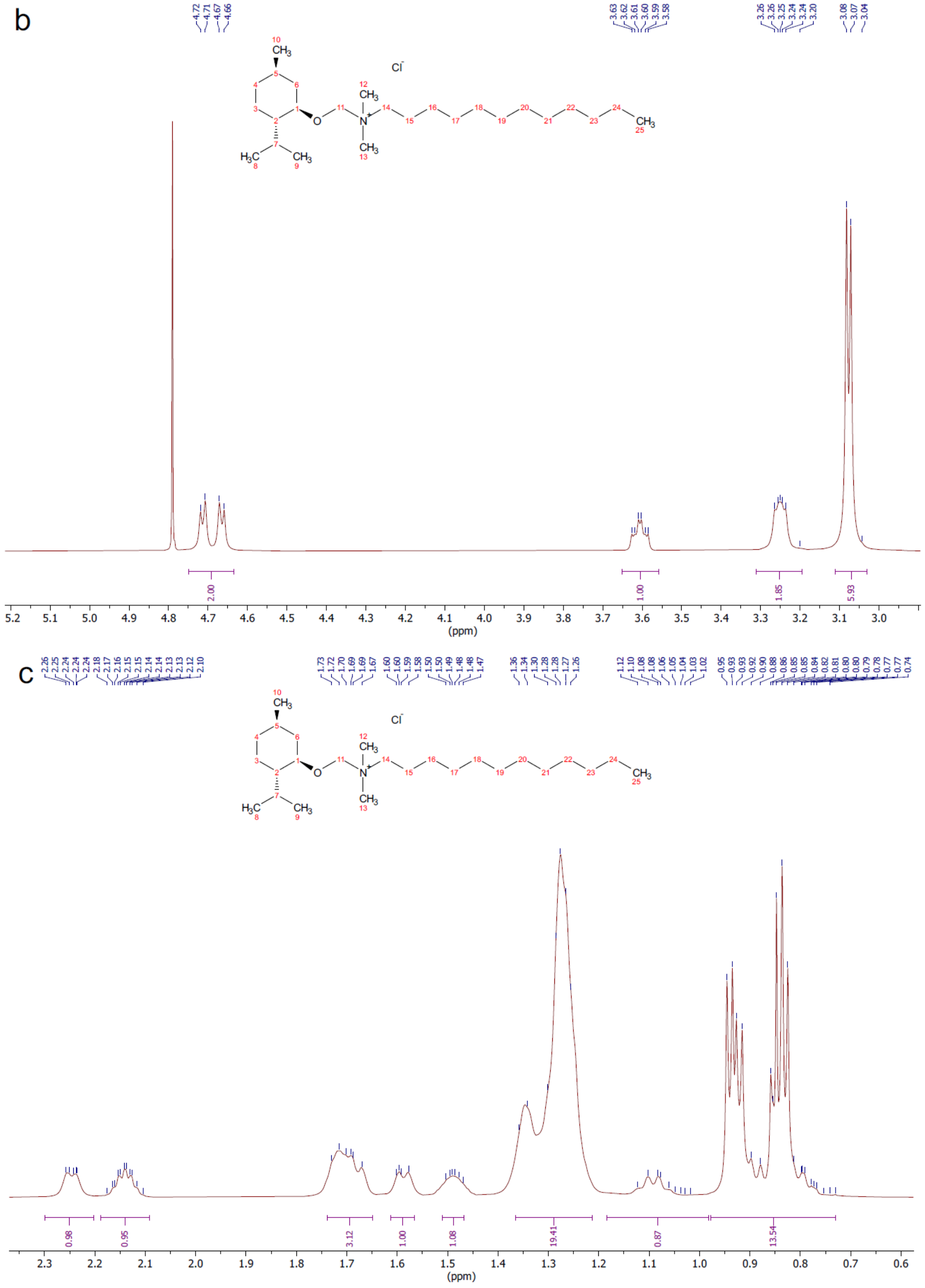

Figure S13. ^{1}H NMR (600 MHz) spectra for C_{12}AmOMCl in D_2O+TMS. a) region -1.0-12.0 ppm, b) region 3.0-5.2 ppm, and c) 0.6-2.3 ppm.

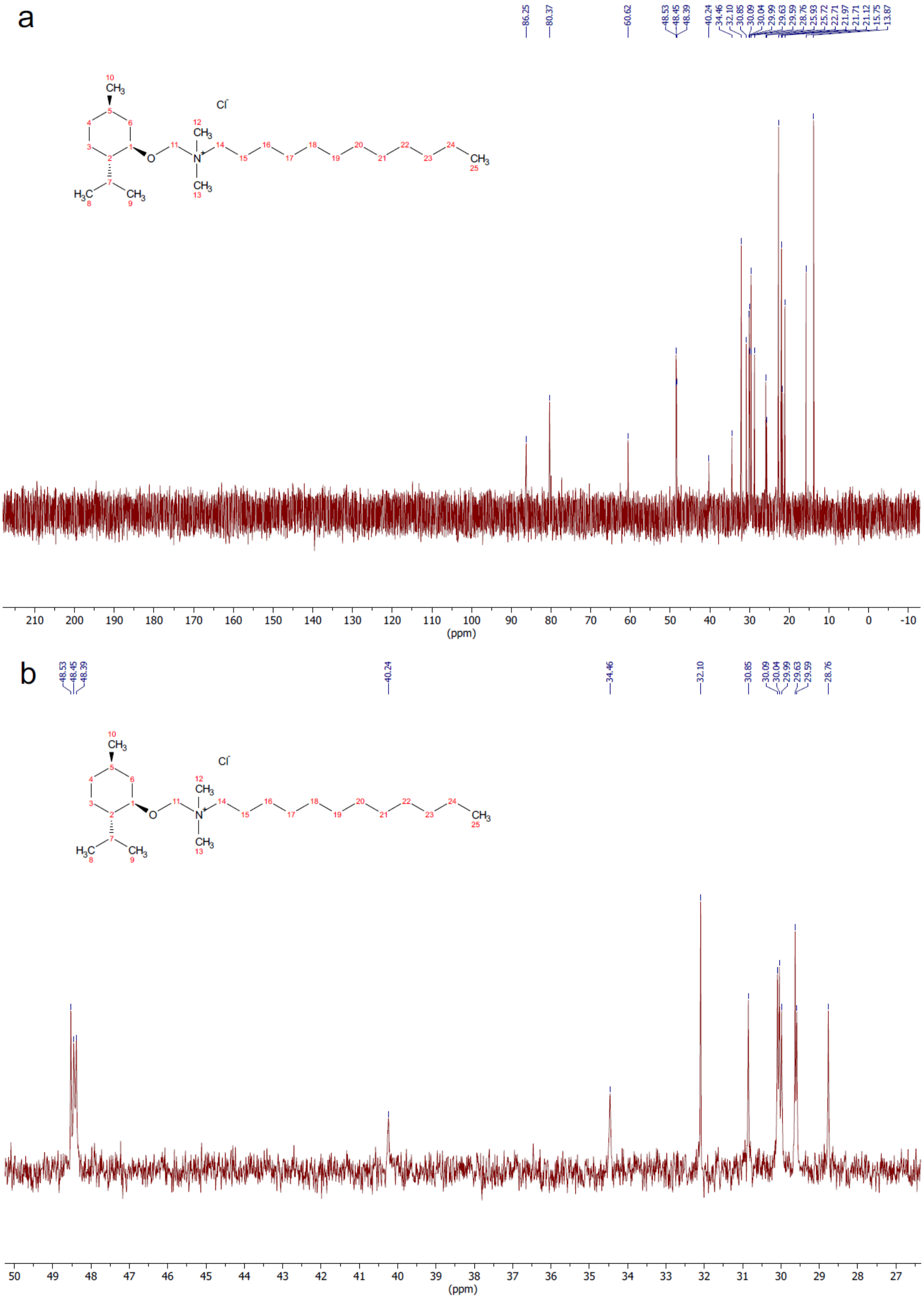

Figure S14. $^{13}C\{^{1}H\}$ NMR (151 MHz) spectra for C_{12}AmOMCl in D_2O+TMS. a) region -10-210 ppm, and b) region 27-50 ppm.

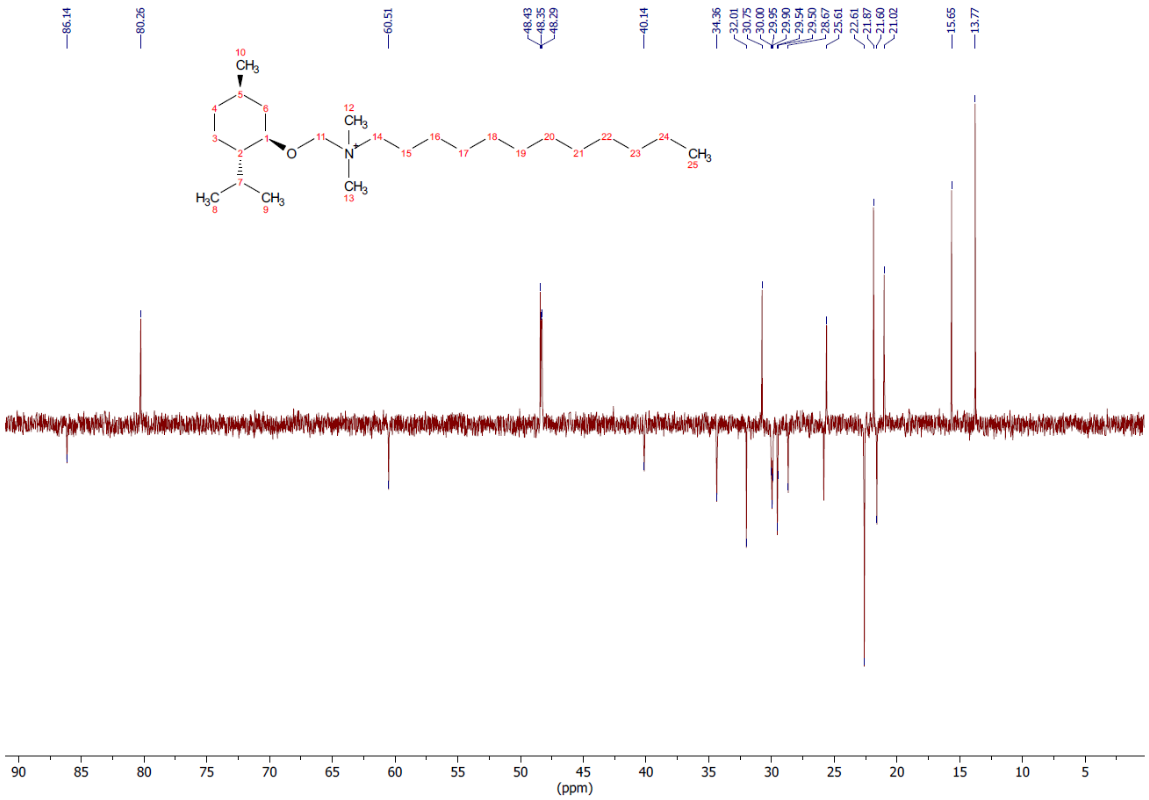

Figure S15. ^{13}C DEPT-135° NMR experiments (151 MHz, D_2O) for C_{12}AmOMCl.

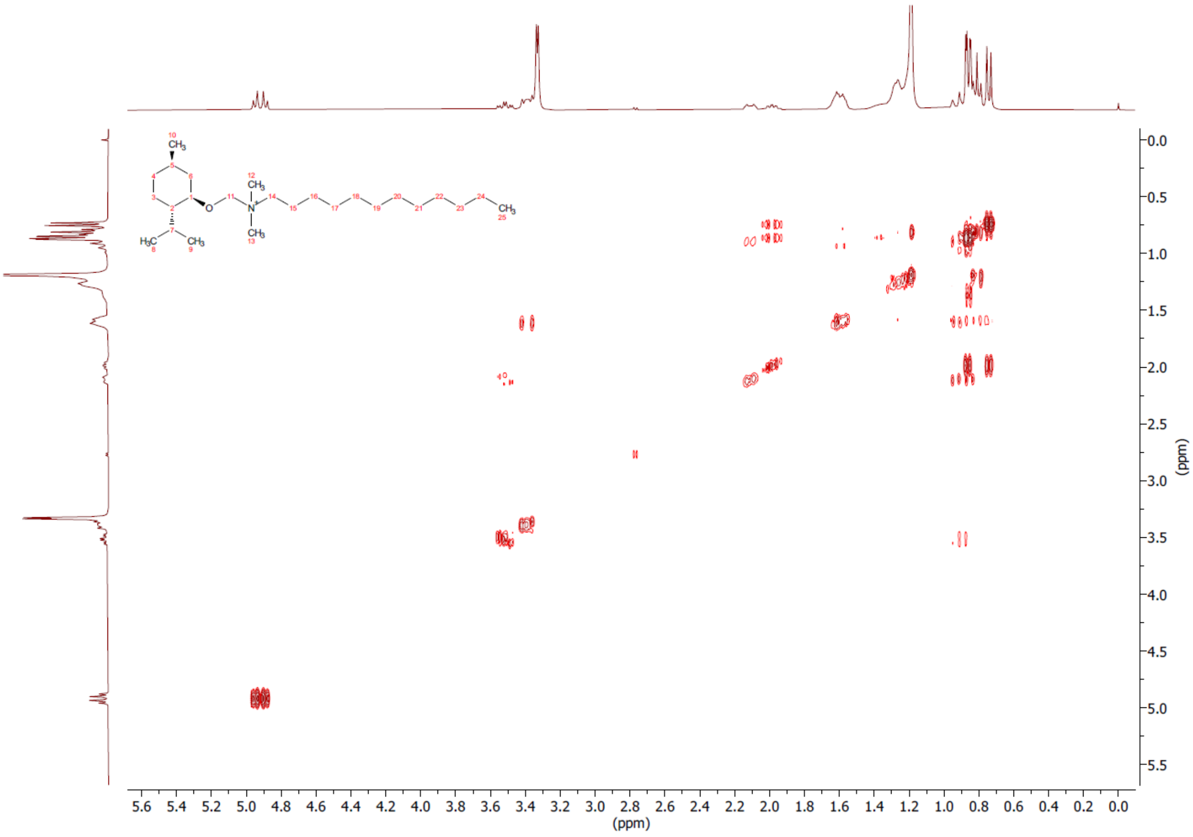

Figure S16. $^{1}H,^{1}H$ COSY experiment (600 MHz) for C_{12}AmOMCl in D_2O.

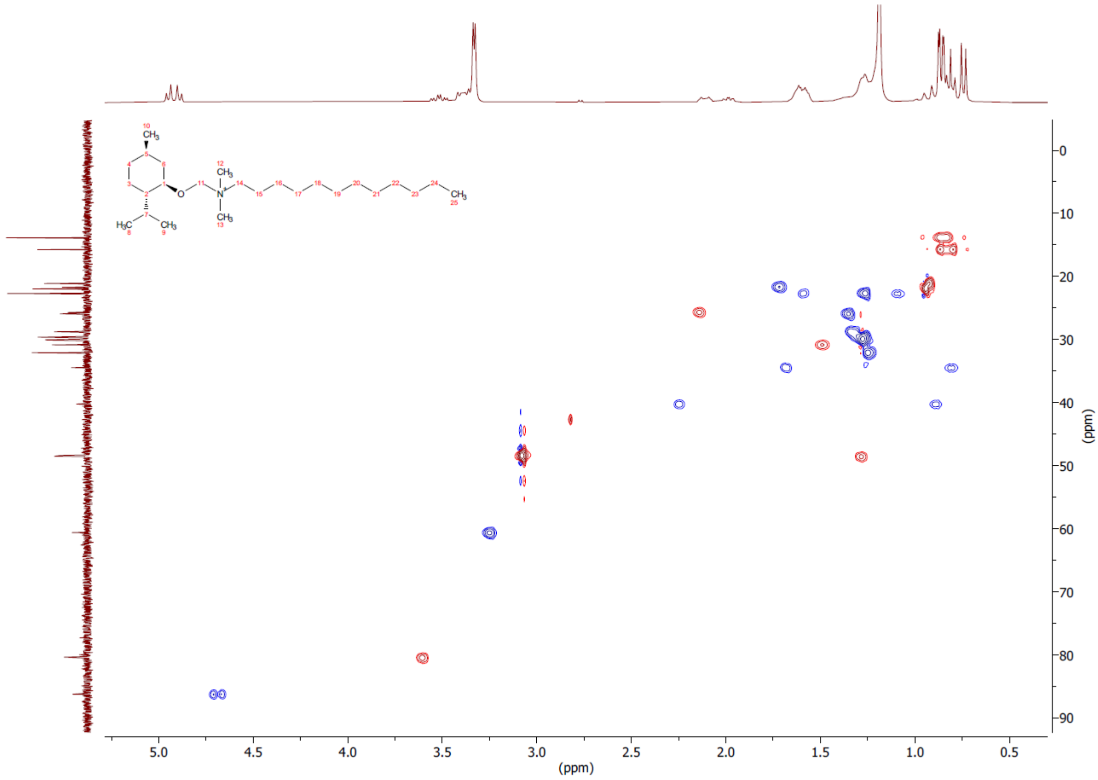

Figure S17. $^{1}H,^{13}C$ multiplicity-resolved HSQC (CH_2-blue, CH/CH_3-red) experiments for C_{12}AmOMCl in D_2O.

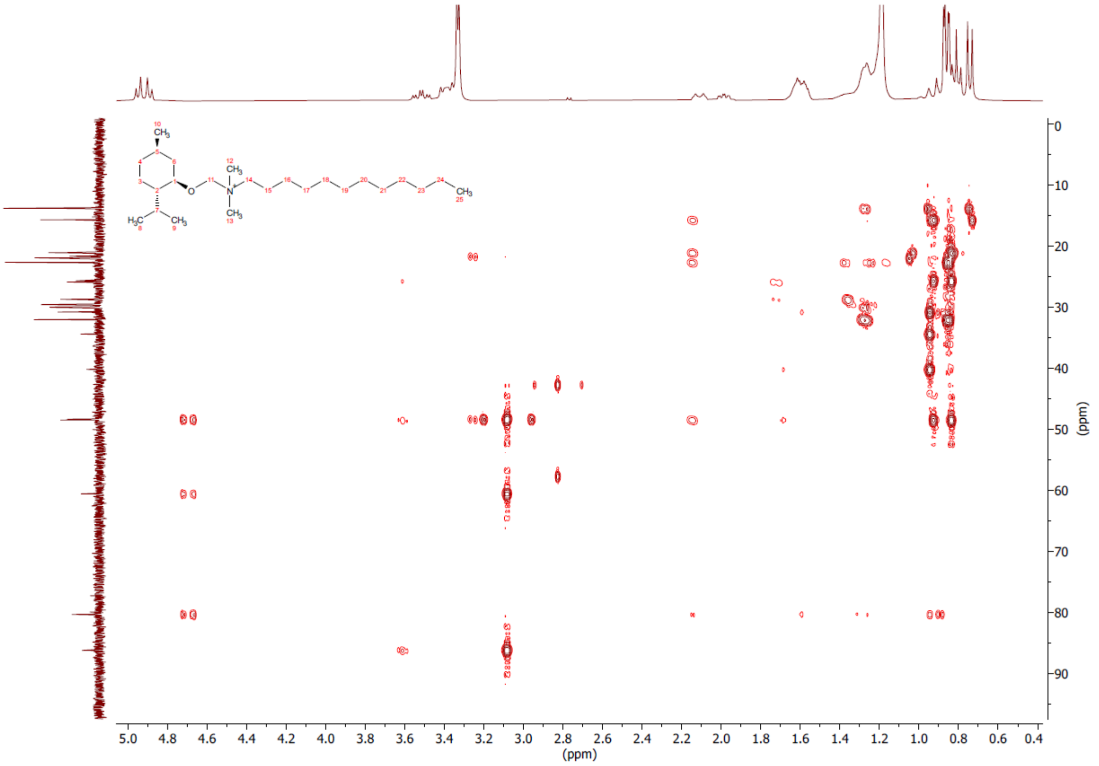

Figure S18. $^{1}H,^{13}C$ multiplicity-resolved HMBC experiments for C_{12}AmOMCl in D_2O.

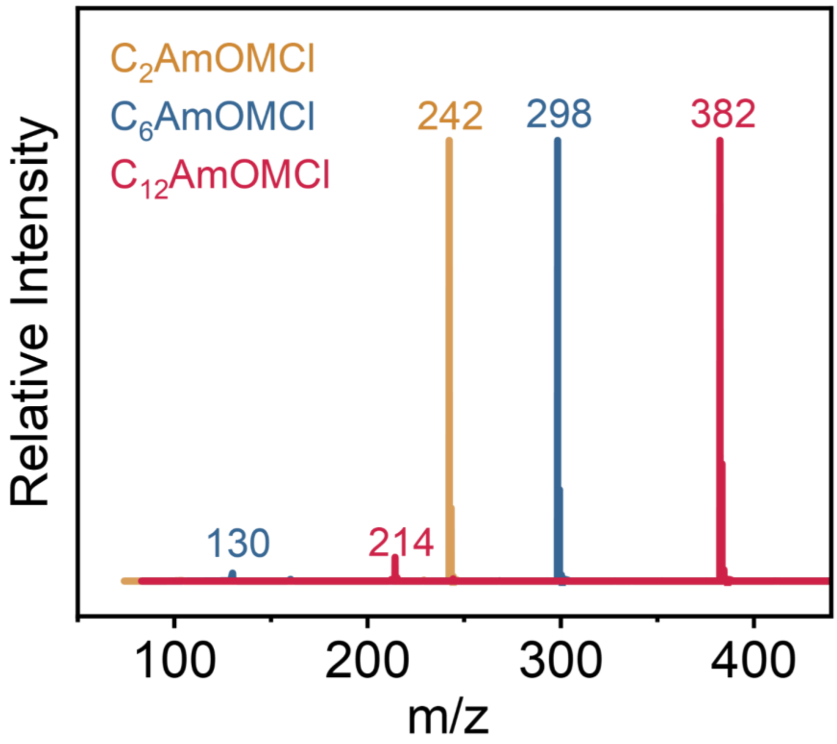

Figure S19. Mass spectra of synthesized bioactive ionic liquids.

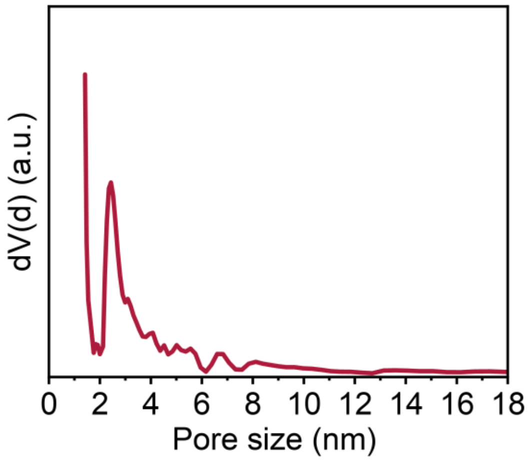

Figure S20. Pore size distribution of ROX carbon based on N_2 physisorption at 77 K (calculated via Density-functional theory (DFT) theory).

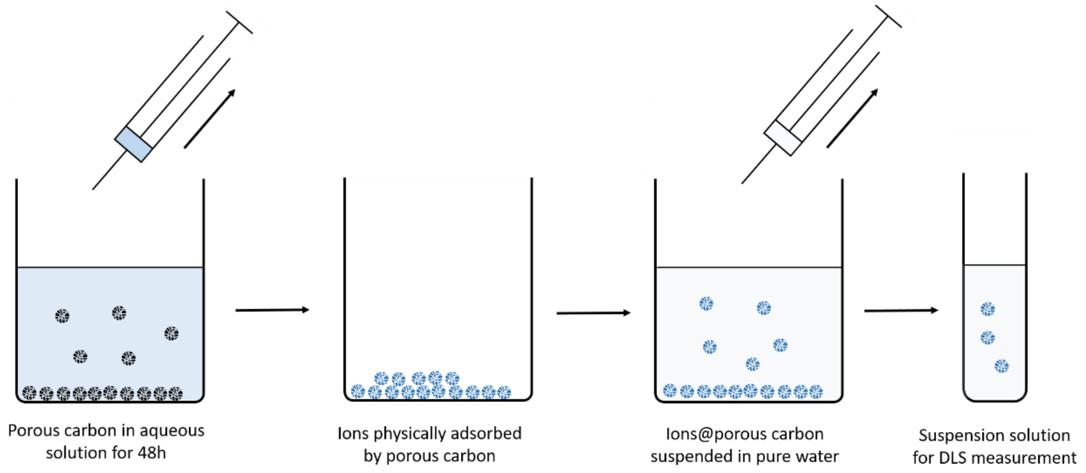

Figure S21. Schematic diagram of ROX carbons treated with different ionic liquid aqueous solutions (0.1 M) for zeta potential measurement.

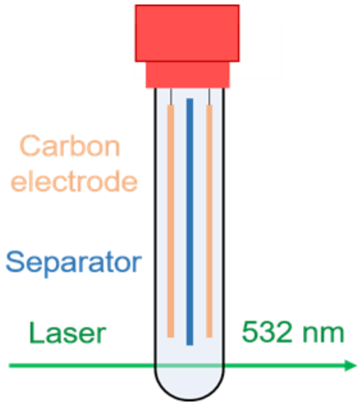

Figure S22. Schematic diagram of *in situ*-Raman concentration measurement.

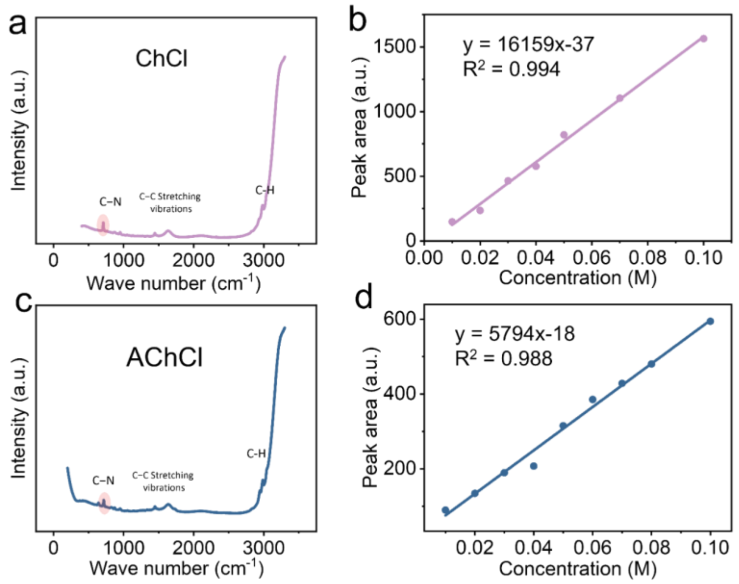

Figure S23. Raman data for ChCl and AChCl. Raman spectra and the fitting line of peak area (red zone) vs. concentration of aqueous a-b) choline chloride (ChCl) and c-d) acetylcholine chloride (AChCl) solutions.

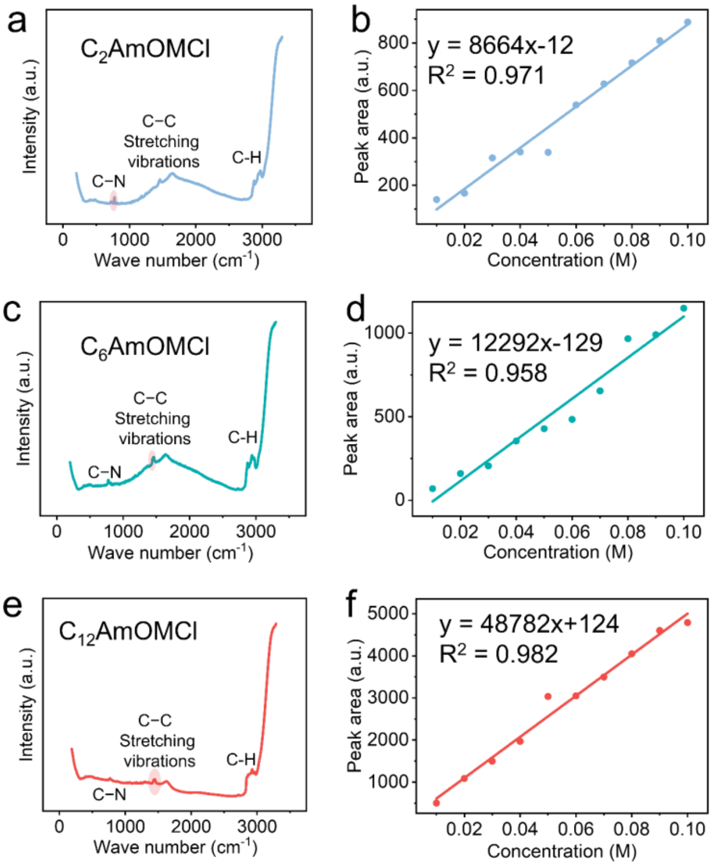

Figure S24. Raman data for C_2AmOMCl, C_6AmOMCl, and C_{12}AmOMCl. Raman spectra and the fitting line of peak area (red zone) vs. concentration of aqueous a-b) C_2AmOMCl, c-d) C_6AmOMCl, and e-f) C_{12}AmOMCl solutions.

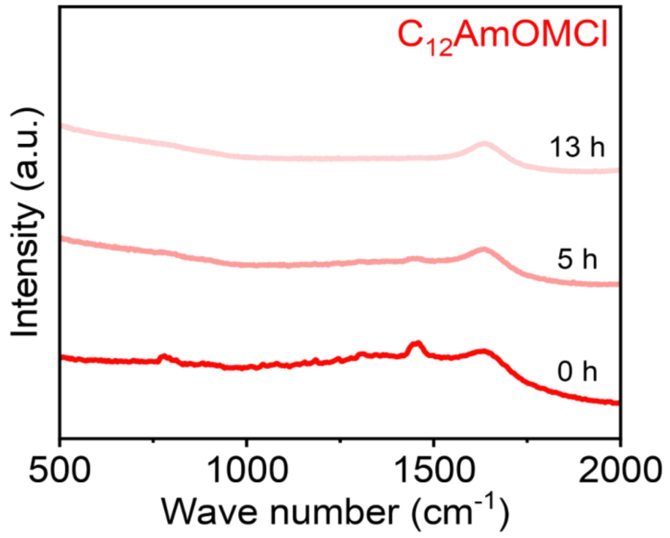

Figure S25. *In situ*-Raman data of C_{12}AmOMCl. Raman spectra of C_{12}AmOMCl aqueous solutions after 5-h physisorption and 8-h electrosorption treatment on ROX carbon electrodes.

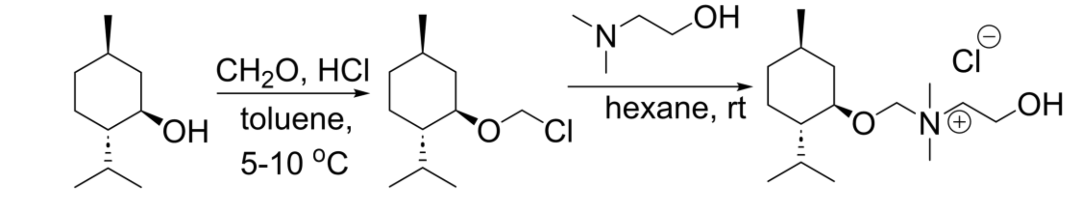

(1*R*,2*S*,5*R*)-(–)-menthol **OMCl** **$C_{2\text{-}OH}$AmOMCl** 99.2%

Figure S26. Synthesis of $C_{2\text{-}OH}$AmOMCl.

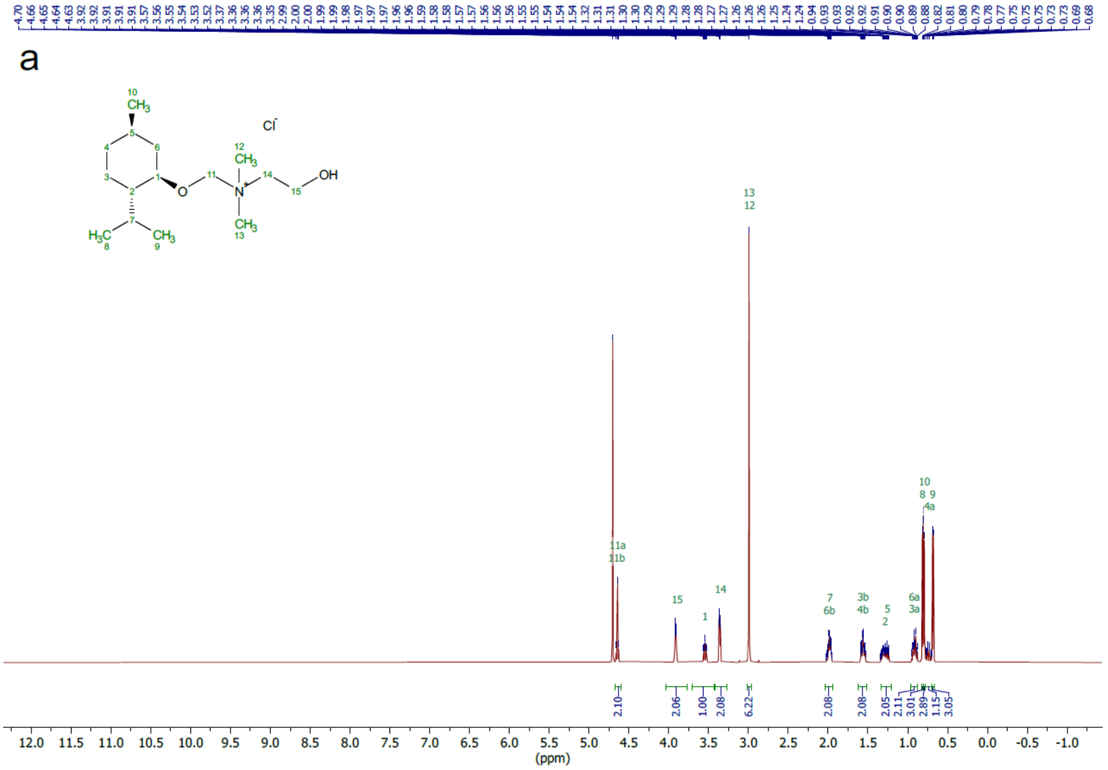

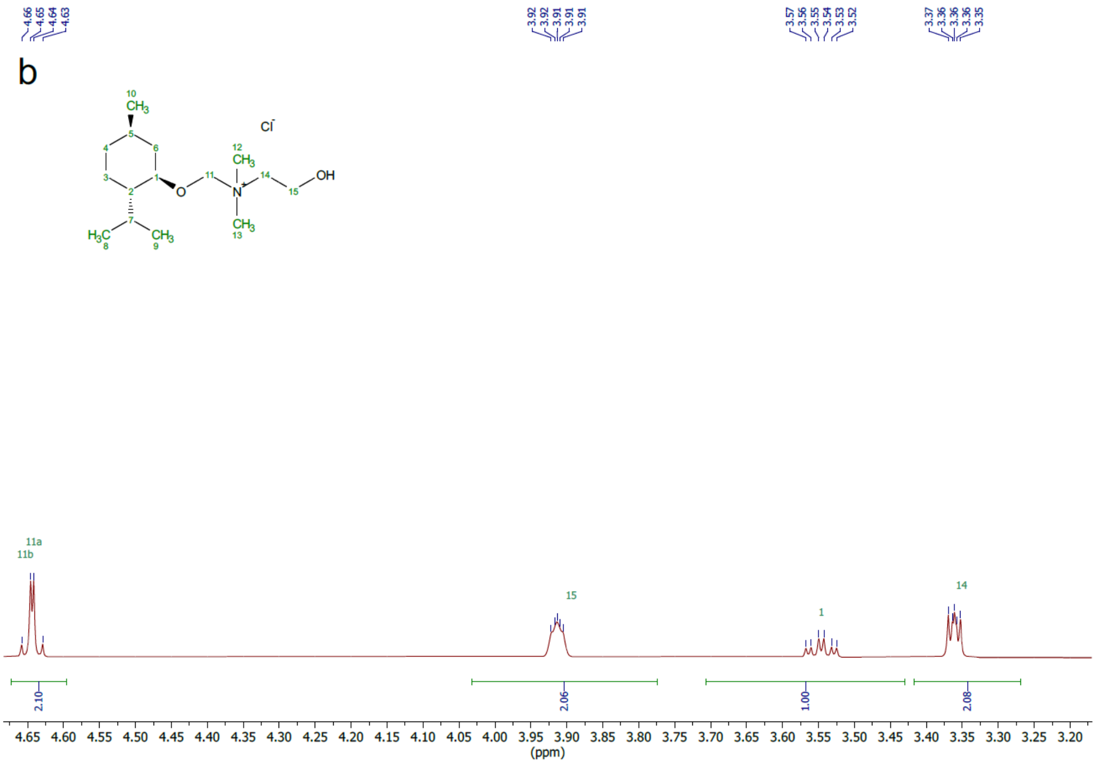
b
Cl-
OH
11a
11b
15
1
14
2.10
2.06
1.00
2.08
(ppm)

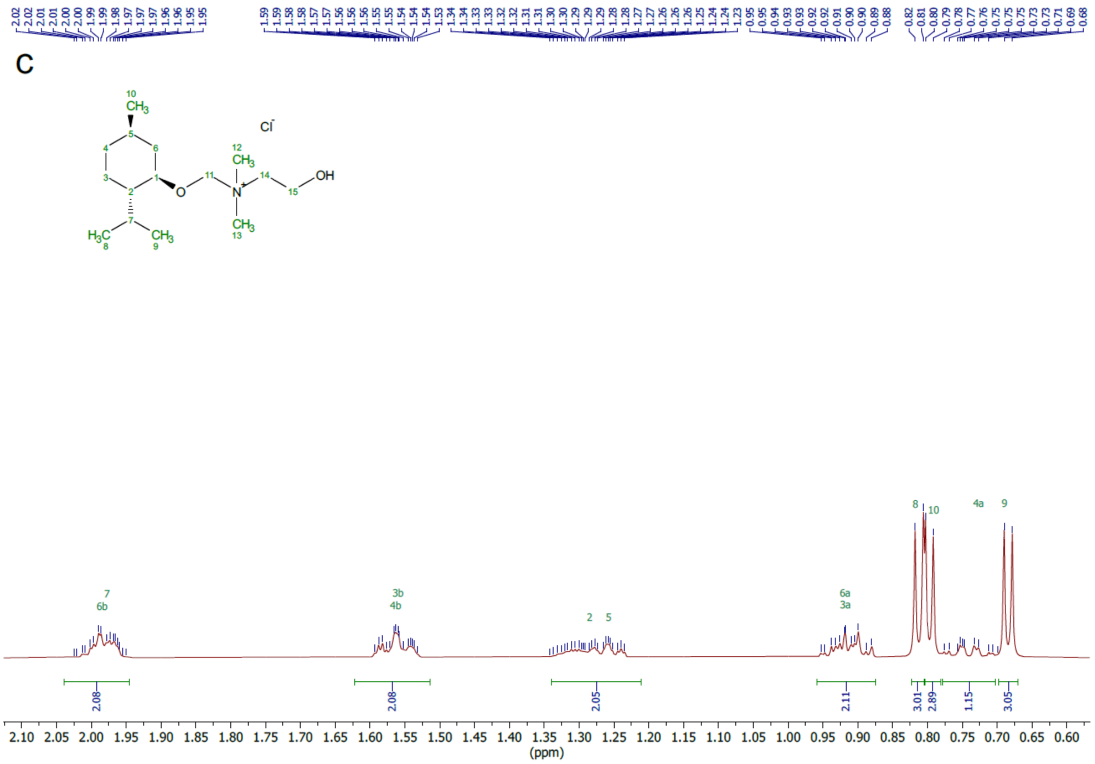
c
Cl-
OH
7
6b
3b
4b
2 5
6a
3a
8
10
4a
9
2.08
2.08
2.05
2.11
3.01
2.89
1.15
3.05
(ppm)

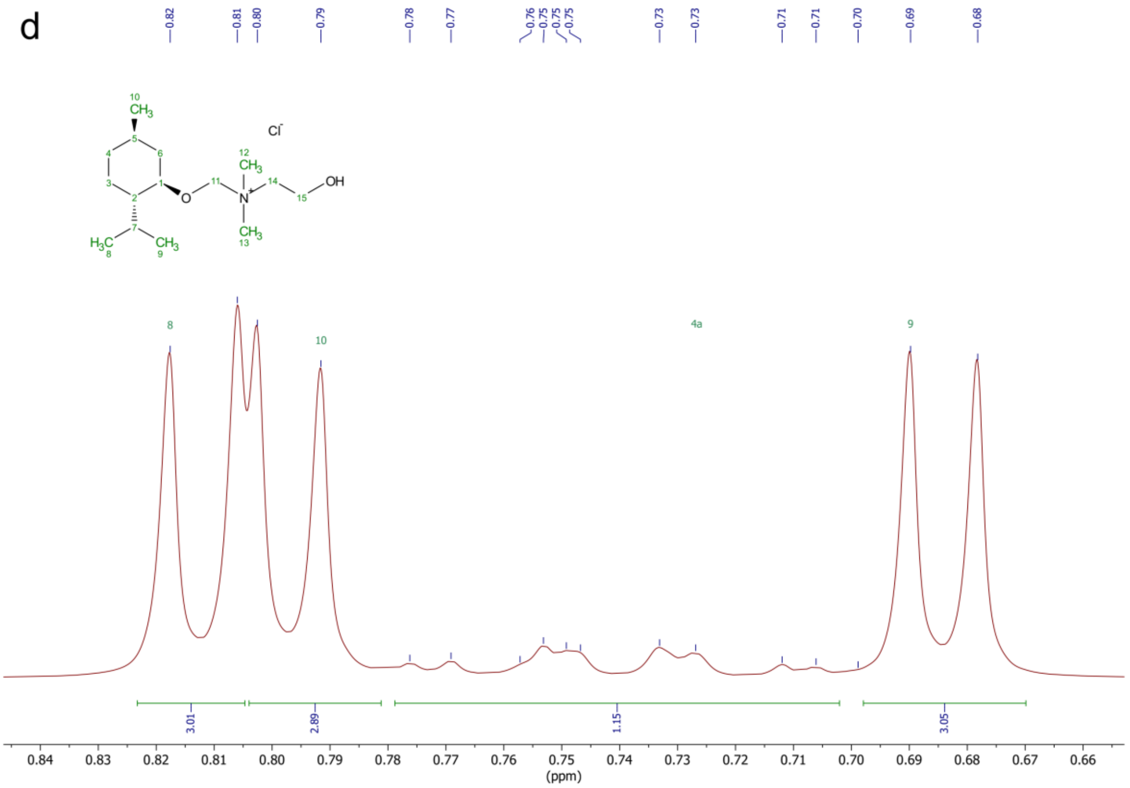

Figure S27. ^{1}H NMR (600 MHz) spectra for $C_{2\text{-}OH}$AmOMCl in D_2O+TMS. a) region -1.0-12.0 ppm, b) region 3.20-4.65 ppm, c) 0.60-2.10 ppm, and d) 0.66-0.84 ppm.

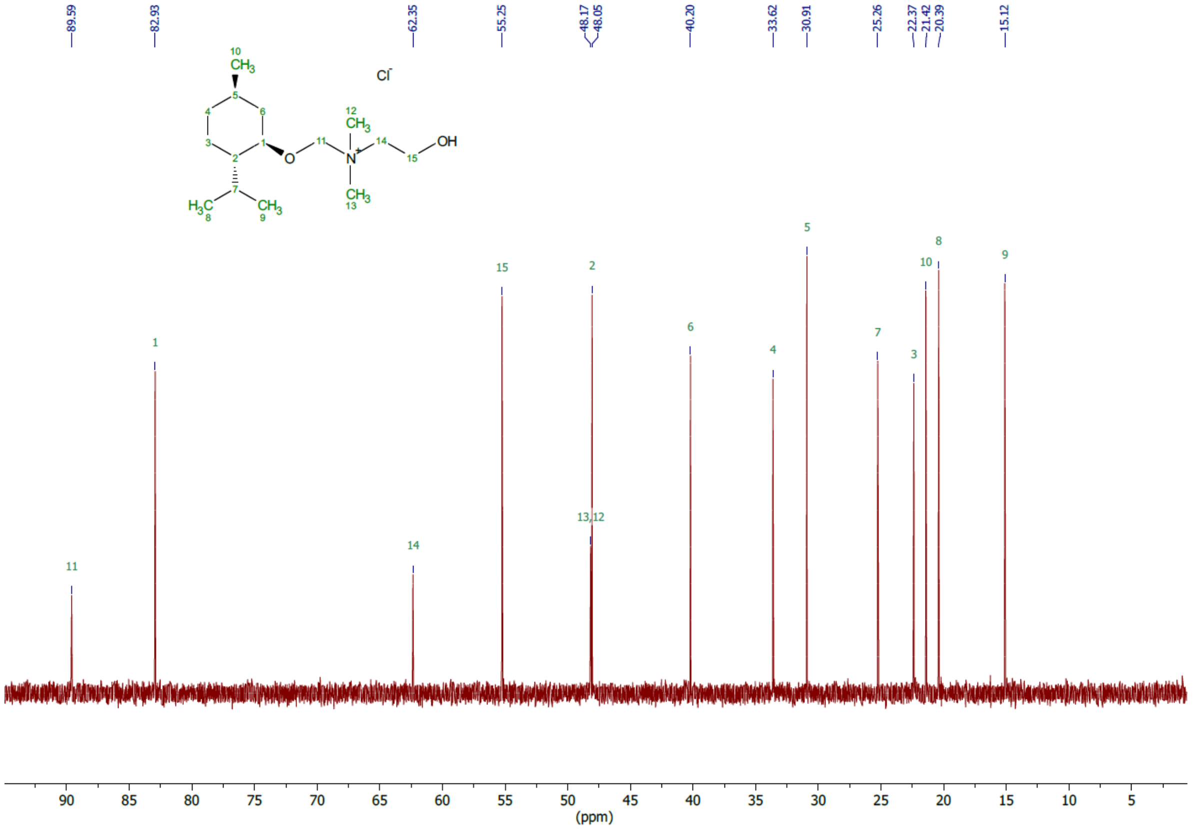

Figure S28. $^{13}C\{^{1}H\}$ NMR (151 MHz) spectra for $C_{2\text{-}OH}$AmOMCl in D_2O+TMS.

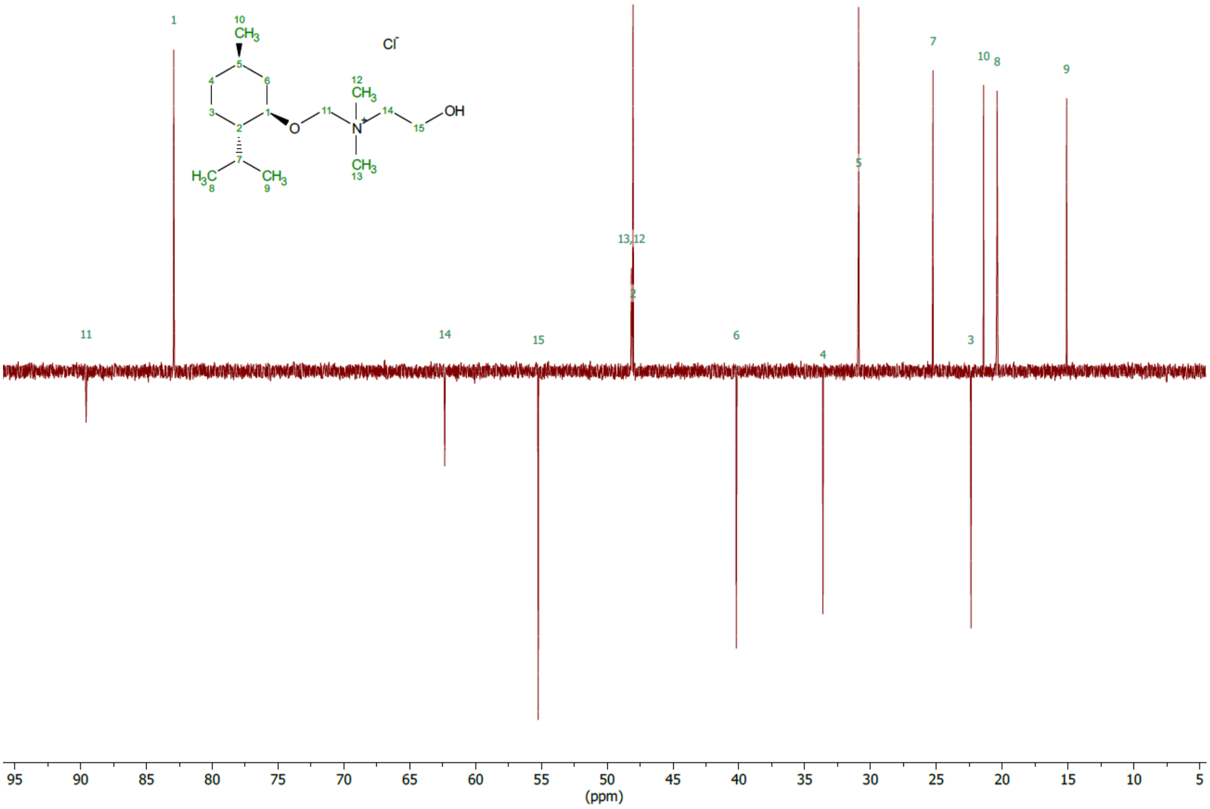

Figure S29. ^{13}C DEPT-135° NMR experiments (151 MHz, D_2O) for $C_{2\text{-}OH}$AmOMCl.

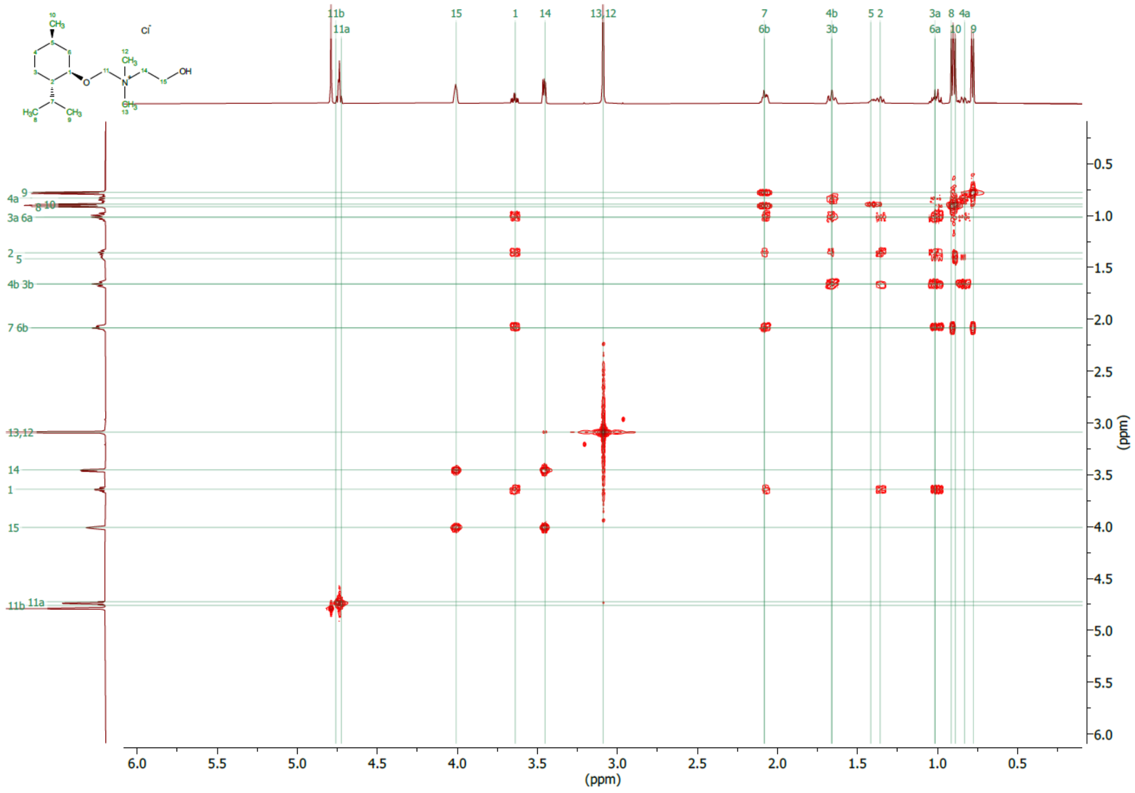

Figure S30. $^1H,^1H$ COSY experiment (600 MHz) for $C_{2\text{-}OH}$AmOMCl in D_2O.

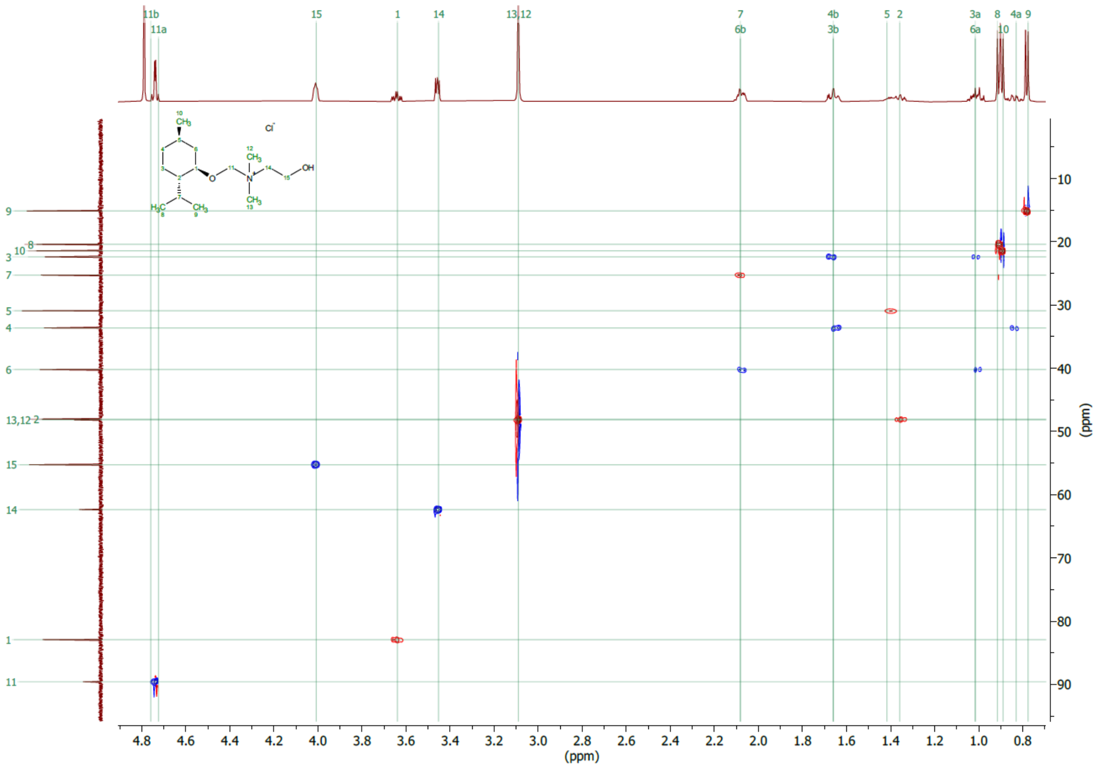

Figure S31. $^{1}H,^{13}C$ multiplicity-resolved HSQC (CH_2-blue, CH/CH_3-red) experiments for $C_{2\text{-}OH}$AmOMCl in D_2O.

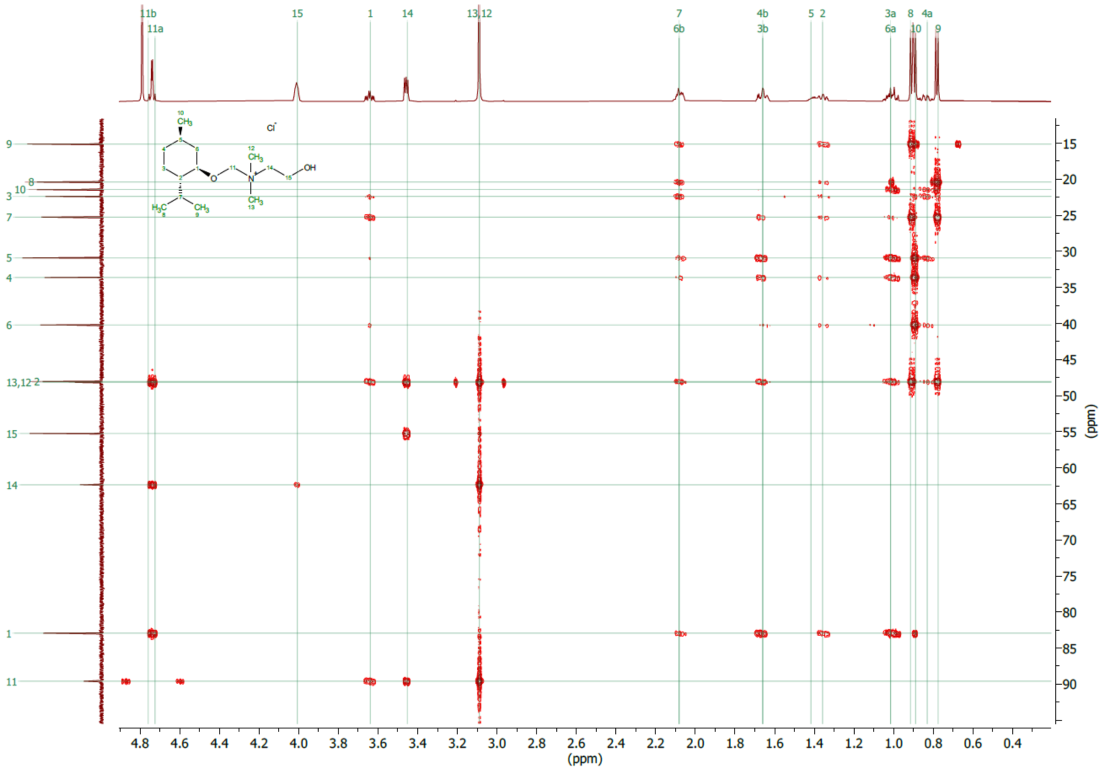

Figure S32. $^{1}H,^{13}C$ multiplicity-resolved HMBC experiments for $C_{2\text{-}OH}$AmOMCl in D_2O.

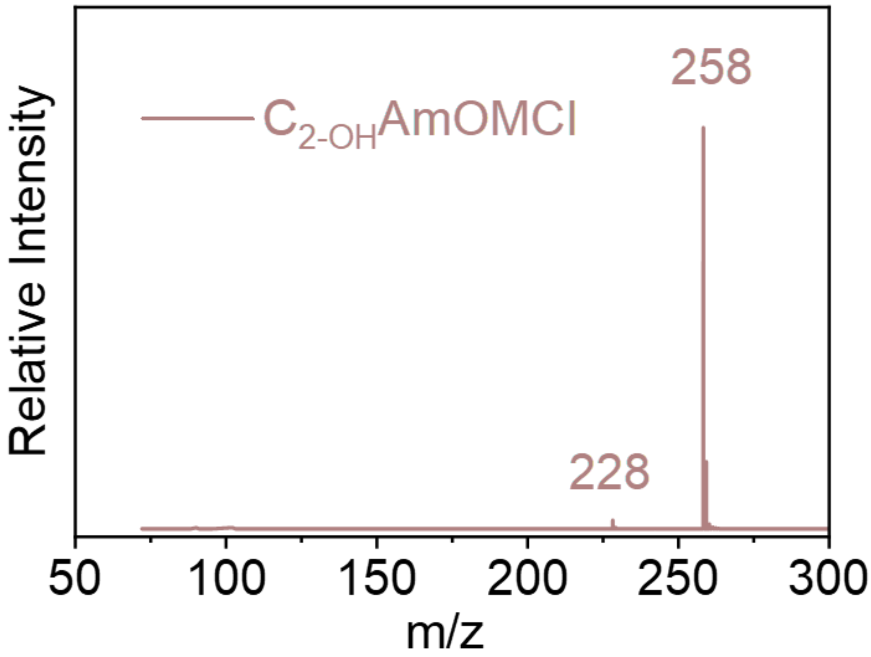

Figure S33. Mass spectrum of $C_{2\text{-}OH}$AmOMCl.

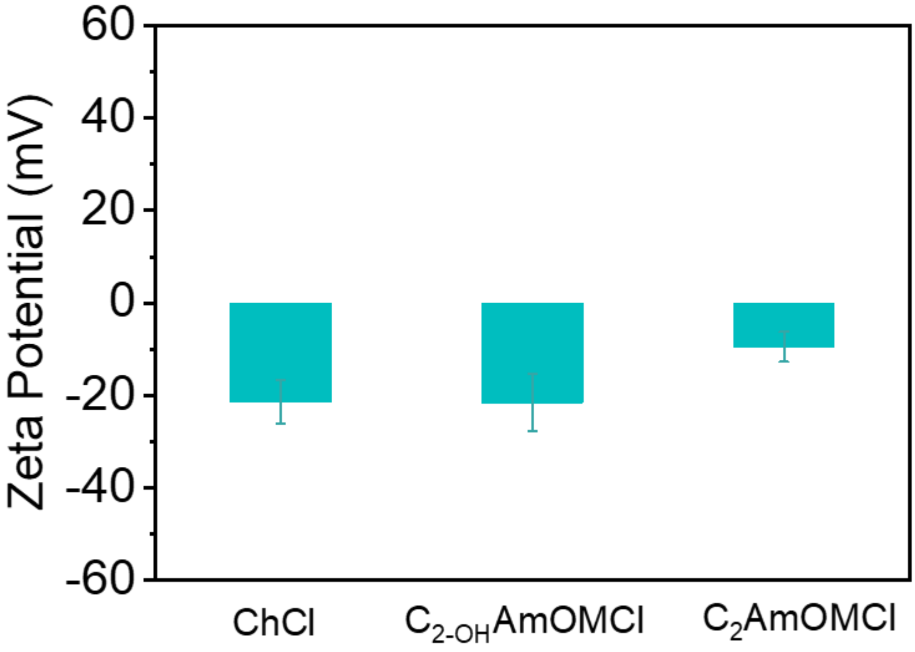

Figure S34. The comparison of zeta potentials (ROX treated with ChCl, $C_{2\text{-}OH}$AmOMCl, and C_2AmOMCl).

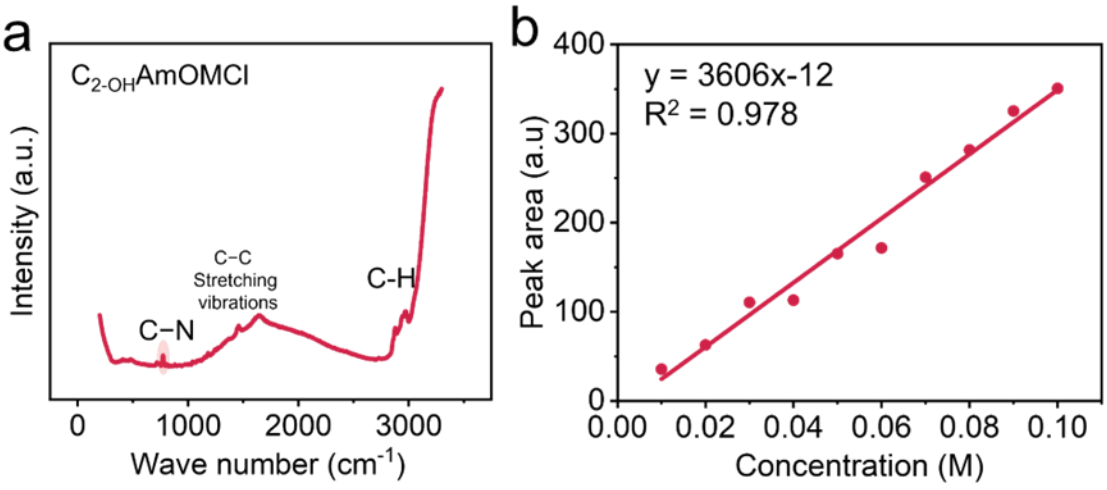

Figure S35. Raman data for $C_{2\text{-}OH}$AmOMCl. a) Raman spectra and b) the fitting line of peak area (red zone) vs. concentration of aqueous $C_{2\text{-}OH}$AmOMCl solutions.

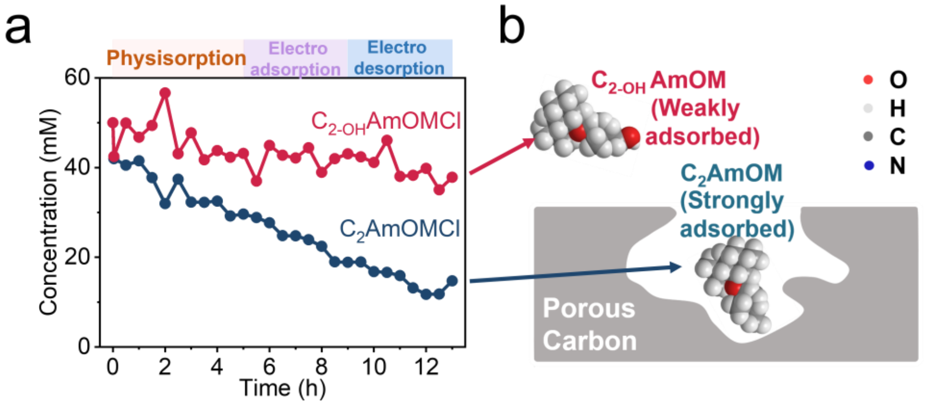

Figure S36. The comparison of C_2AmOMCl and $C_{2\text{-}OH}$AmOMCl. a) Concentration profiles vs. time for C_2AmOMCl and $C_{2\text{-}OH}$AmOMCl aqueous solutions in a ROX-based symmetric capacitor. b) Schematic diagram of the adsorption behaviors of C_2AmOM and $C_{2\text{-}OH}$AmOM cations in porous carbon (ROX).

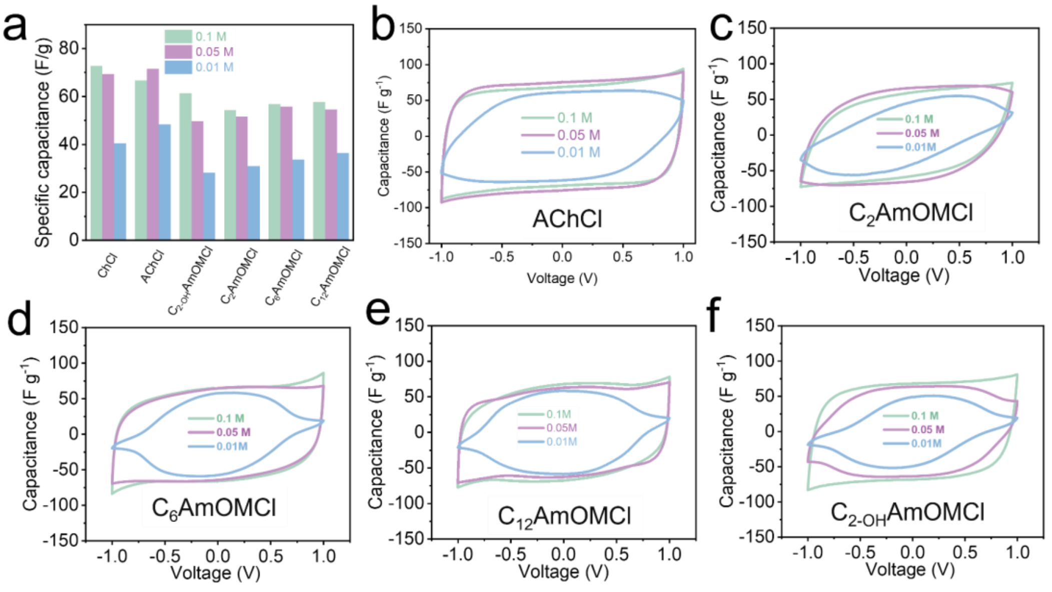

Figure S37. Capacitance evaluation. a) Capacitance comparison of ROX-based symmetric capacitors in different concentrated aqueous electrolytes (the capacitance values for ChCl electrolytes from our former report[1]). The cyclic voltammetry curves of ROX-based symmetric capacitors in 0.1, 0.05, and 0.01 M, b) AChCl, c) C_2AmOMCl, d) C_6AmOMCl, e) C_{12}AmOMCl, and f) $C_{2\text{-}OH}$AmOMCl electrolytes at a scan rate of 1 mV s^{-1}.

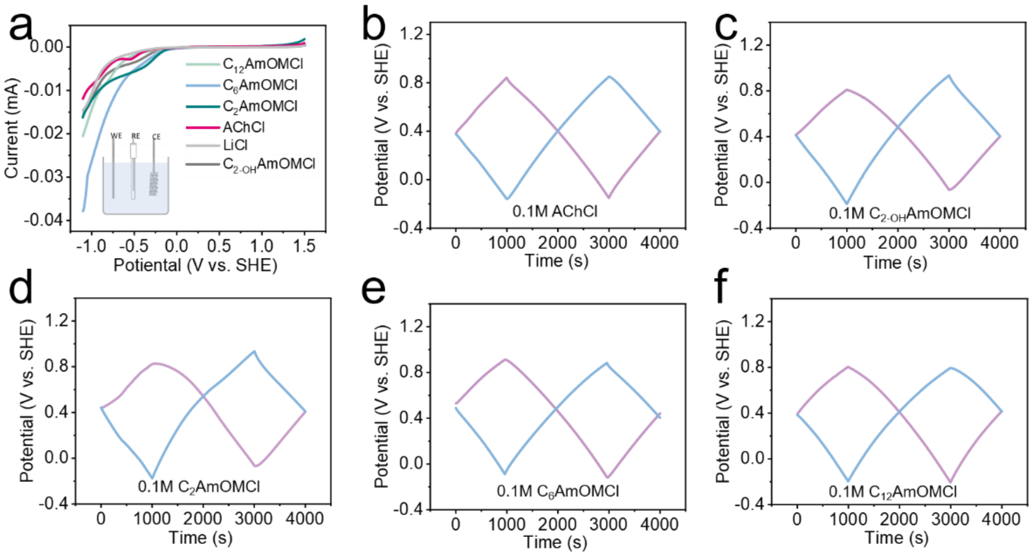

Figure S38. LSV measurement. a) The electrochemical stabilities of 0.1 M different aqueous solutions and the scheme of the 3-electrode cell for LSV measurement. b-f) The potential changes of the working electrode (E_{we}, purple curves) and counter electrode (E_{ce}, blue curves) of the ROX-based system capacitor in different aqueous electrolytes during cyclic voltammetry measurement.

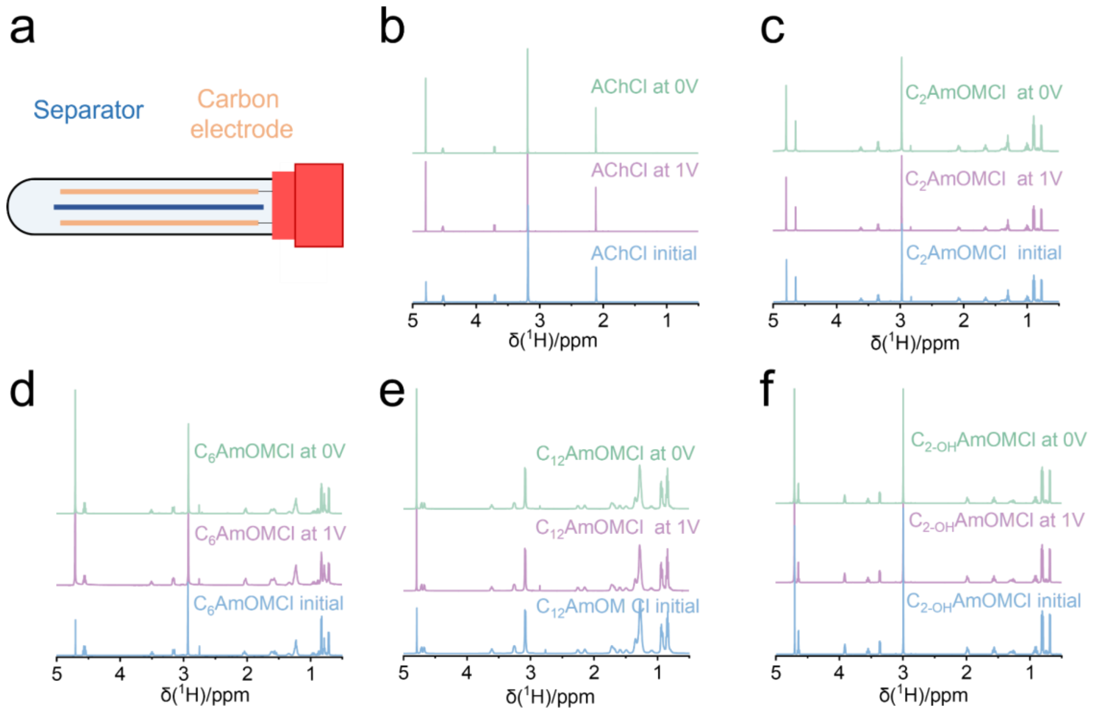

Figure S39. *Ex situ*-liquid NMR data. a) Scheme of the tube cell for *ex situ*-NMR experiments. b-f) ^{1}H liquid NMR spectra of 0.1 M AChCl, C_2AmOMCl, C_6AmOMCl, C_{12}AmOMCl, and $C_{2\text{-}OH}$AmOMCl in ^2H_2O for initial, electrically adsorbed (1 V), and desorbed (0 V) electrolytes in ROX-based symmetric capacitors.

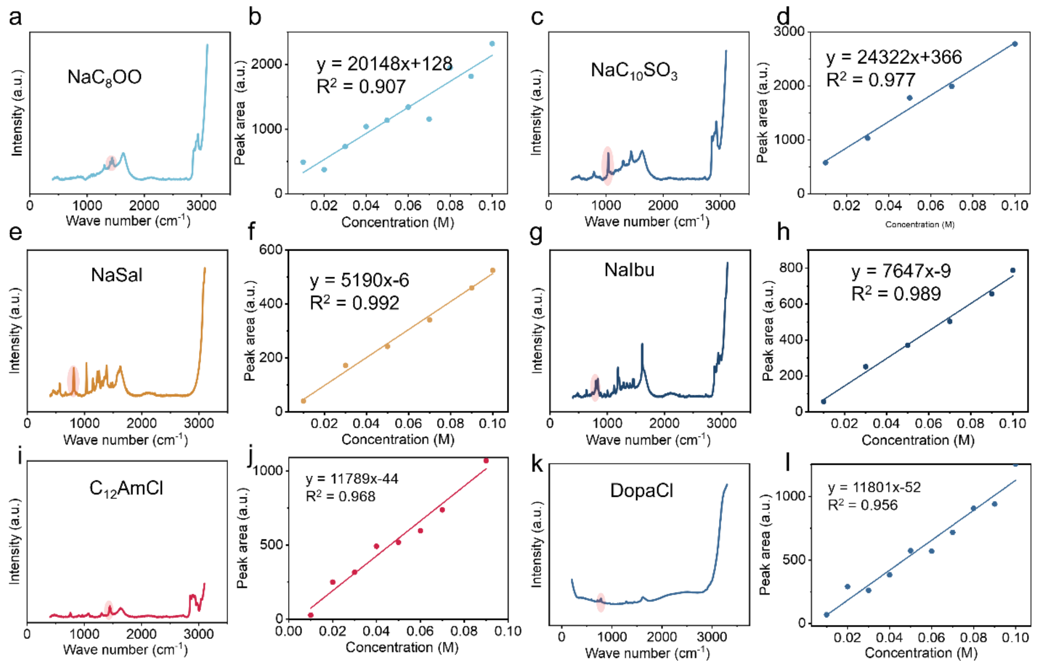

Figure S40. Raman data for NaC_8OO, $NaC_{10}SO_3$, NaSal, NaIbu, C_{12}AmCl, and DopaCl. a, c, e, g, i, k) Raman spectra and b, d, f, h, j, l) the fitting line of peak area (red zone) vs. concentration of aqueous solutions of NaC_8OO, $NaC_{10}SO_3$, NaSal, NaIbu, C_{12}AmCl, and DopaCl.

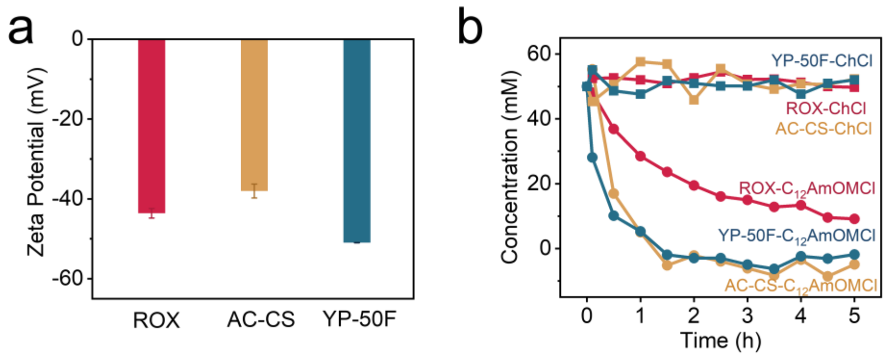

Figure S41. The interaction investigation of AC-CS and YP-50F nanoporous carbons. a) The zeta potentials of ROX, AC-CS, and YP-50F nanoporous carbons. b) Concentration profiles vs. time for 0.1 M ChCl and C_{12}AmOMCl aqueous solutions induced by electrolyte ion physisorption in ROX, AC-CS, and YP-50F nanoporous carbons.

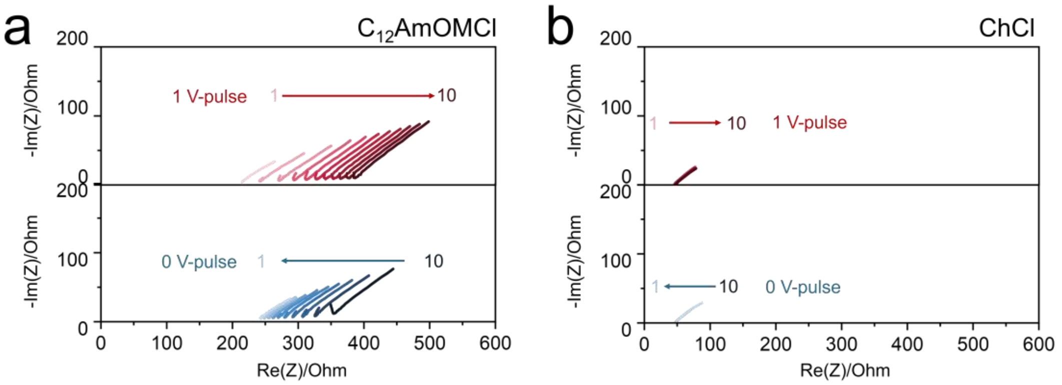

Figure S42. The experimental impedance data of D-Cap in 4-terminal devices. The impedance changes of D-Cap during voltage pulsing (10 times of 1 V-pulses and 10 times of 0 V-pulses) to the M-Cap (t_p = 20 s, t_r = 20 s) in a) 0.1 M C_{12}AmOMCl and b) 0.1 M ChCl electrolytes.

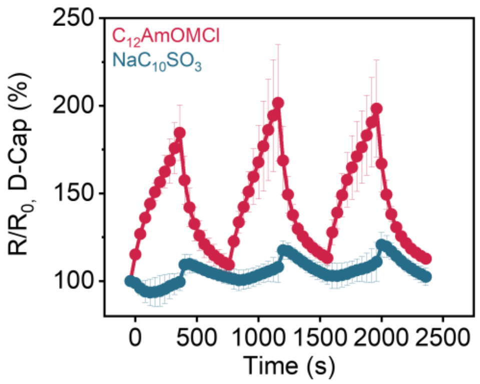

Figure S43. The switching resistance retention in C_{12}AmOMCl and $NaC_{10}SO_3$ electrolytes. The switching resistance retention of D-Cap during voltage pulsing to the M-Cap (t_p = 20 s, t_r = 20 s) in 0.1 M C_{12}AmOMCl and 0.1 M $NaC_{10}SO_3$ electrolytes.

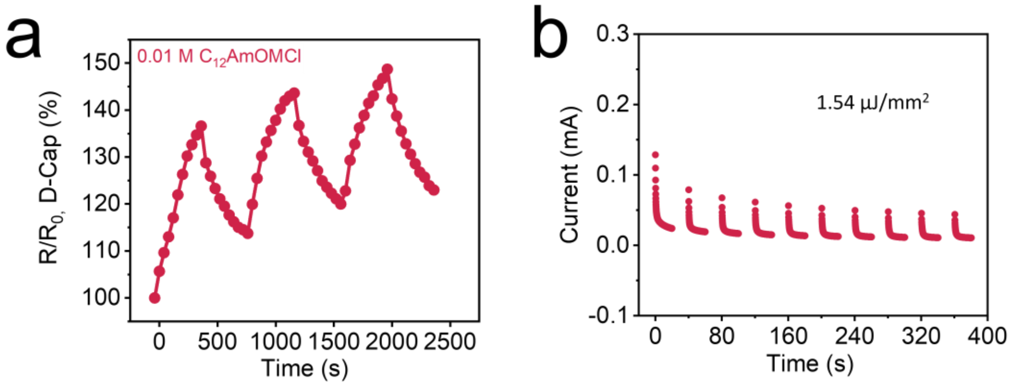

Figure S44. The investigation of switching energy. a) The switching resistance retention (R/R_0) of D-Cap during voltage pulsing to the M-Cap (t_p = 20 s, t_r = 20 s) in 0.01 M C_{12}AmOMCl electrolyte. b) I-t curves of M-Cap during 10 times of 1 V-pulses (t_p = 20 s, t_r = 20 s) in 0.01 M C_{12}AmOMCl electrolyte and the average switching energy of 1 pulse.

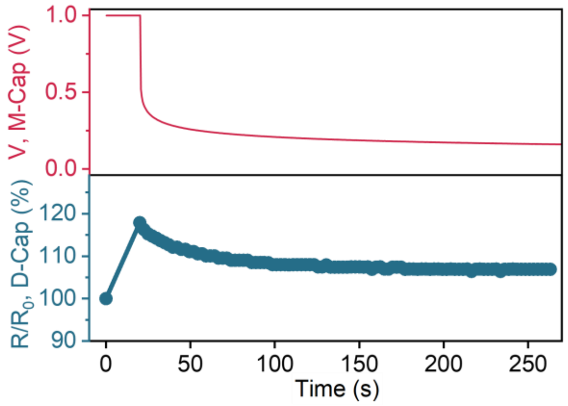

Figure S45. The resistance state of D-Cap after 1 time of 1 V-pulses (t_p = 20 s) to the M-Cap in a 0.1 M C_{12}AmOMCl electrolyte.

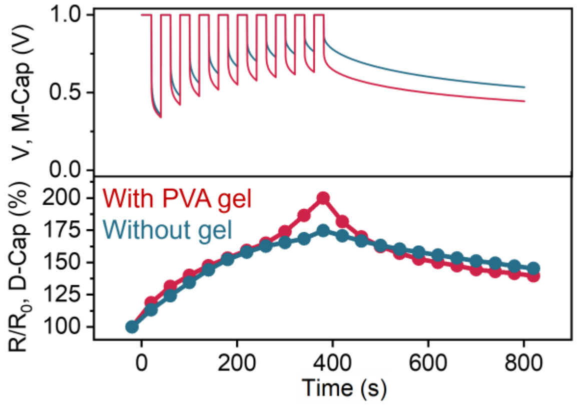

Figure S46. The resistance retention comparison with/without polyvinyl alcohol (PVA) gel. The resistance state of D-Cap after 10 times of 1 V-pulses (t_p = 20 s, t_r = 20 s) to the M-Cap in a 0.1 M C_{12}AmOMCl electrolyte with/without PVA gel.

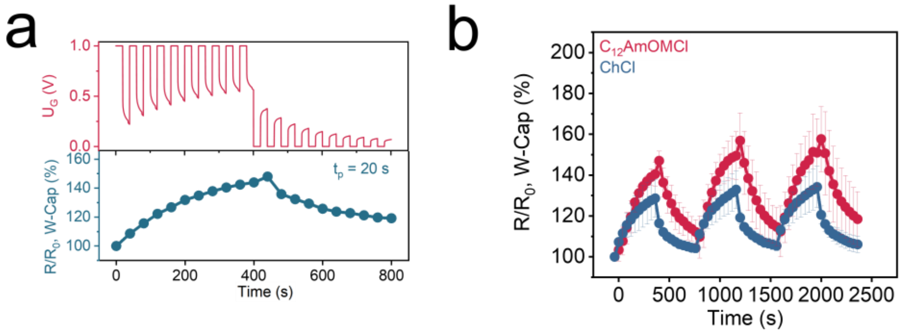

Figure S47. The resistance changes in 3-terminal devices. a) The resistance retention (R/R_0, R_0: the initial resistance of W-Cap) of the W-Cap during voltage pulsing (10 times of 1 V-pulses and 10 times of 0 V-pulses) to U_G (t_p =20 s, t_r =20 s) in a 0.1 M C_{12}AmOMCl electrolyte. b) The resistance retention of D-Cap during voltage pulsing to U_G in 0.1 M C_{12}AmOMCl and ChCl electrolytes.

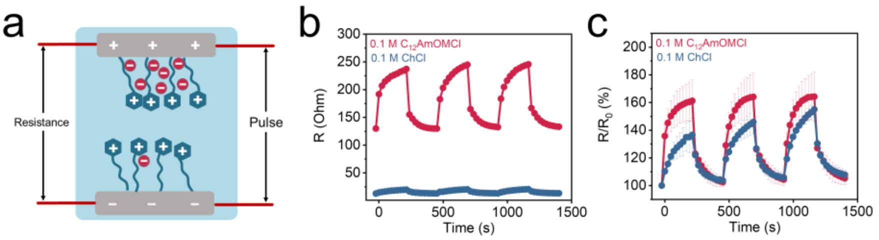

Figure S48. The resistance changes in 2-terminal devices. a) Schematic diagram of a 2-terminal ionic memristor device in a C_{12}AmOMCl electrolyte. b) The actual values of resistance changes of 2 electrodes in 0.1 M C_{12}AmOMCl and 0.1 M ChCl electrolytes after each voltage pulsing (10 times of 1 V-pulses and 10 times of 0 V-pulses, t_p =20 s, t_r =20 s) to these two electrodes. c) The relative resistance retention (R/R_0, R_0: the initial resistance of the 2-terminal devices).

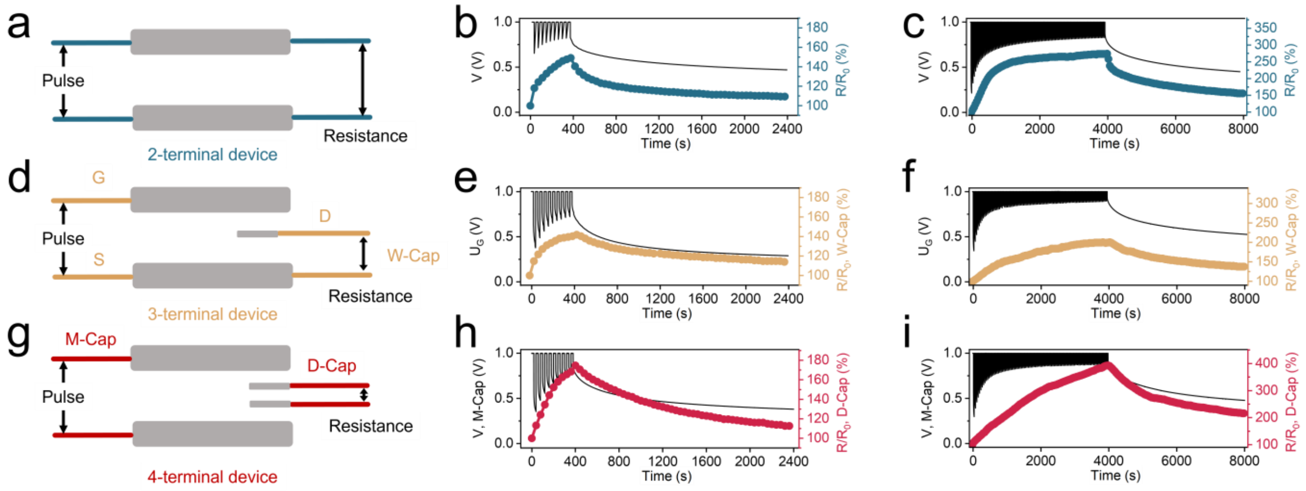

Figure S49. The comparison of resistance retention in 2-, 3-, and 4-terminal devices. a) Schematic diagram of a 2-terminal memristor device. The resistance changes of 2 electrodes after b) 10 and c) 100 times of 1 V-pulses (t_p =20 s, t_r =20 s) applied to these two electrodes in a 0.1 M C_{12}AmOMCl electrolyte. d) Schematic diagram of a 3-terminal memristor device. The resistance changes of W-Cap after e) 10 and f) 100 times of 1 V-pulses (t_p =20 s, t_r =20 s) applied to G and S electrodes in a 0.1 M C_{12}AmOMCl electrolyte. g) Schematic diagram of a 4-terminal memristor device. The resistance changes of D-Cap after h) 10 and i) 100 times of 1 V-pulses (t_p =20 s, t_r =20 s) applied to D-Cap in a 0.1 M C_{12}AmOMCl electrolyte.

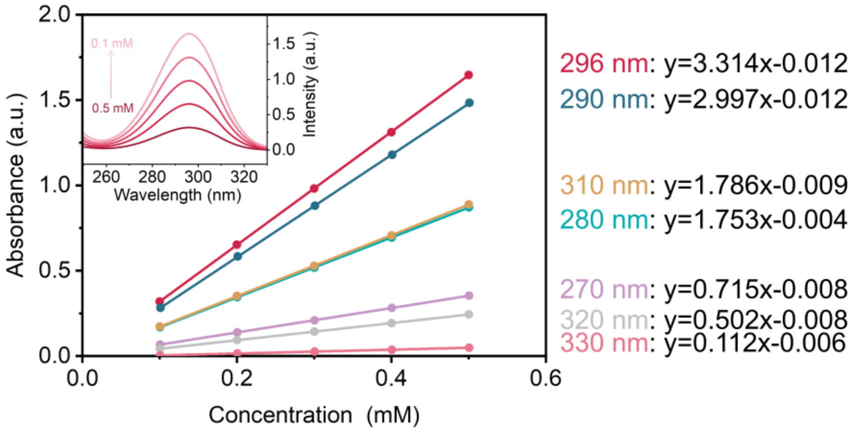

Figure S50. UV/Vis spectra of NaSal solutions and the calibration curves under various wavelengths.

Table S1. Chemical information of all synthesized ionic compounds and purchased ChCl and AChCl. Abbreviation, yield, cationic active substance content, empirical formula, and size[5] for all synthesized ionic compounds with a (1*R*,2*S*,5*R*)-(–)-menthol moiety, commercial ChCl and AChCl.

Abbreviation	Structure	Yield[a,b] (%)	Surfactant content[c] (%)	Empirical formula	Size (nm)
$C_2AmOMCl$	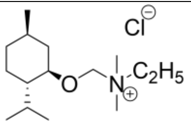	99.9	99.8[d]	$C_{15}H_{32}NOCl$	1.24×1.03×0.59
$C_6AmOMCl$	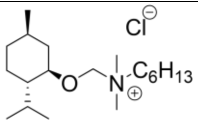	99.4	99.7[e]	$C_{19}H_{40}NOCl$	1.73×1.02×0.59
$C_{12}AmOMCl$	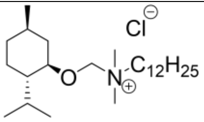	97.9	99.8[f]	$C_{25}H_{52}NOCl$	2.48×1.03×0.59
$C_{2\text{-}OH}AmOMCl$	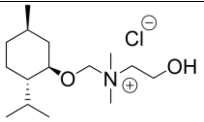	99.2	-	$C_{15}H_{32}NO_2Cl$	1.34×1.02×0.59
ChCl	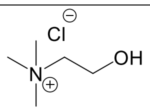	-[g]	-	$C_5H_{14}NOCl$	0.86×0.58×0.62
AChCl	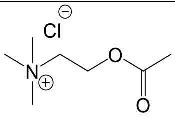	-[g]	-	$C_7H_{16}NO_2Cl$	1.10×0.58×0.62

[a] Isolated yield after purification and drying; [b] Accuracy ± 0.5%; [c] Accuracy ± 0.1%; [d] Lit data[3] for = 97.0%; [e] Lit data[3] or = 97.6%; [f] Lit data[3] for = 99.8%; [g] the compound was purchased commercially.

References

[1] P. Li, Y. Bräuniger, J. Kunigkeit, H. Zhou, M. R. Ortega Vega, E. Zhang, J. Grothe, E. Brunner, S. Kaskel, *Angew. Chem. Int. Edit.* **2022**, *61*, e202212250.

[2] ISO 2871-2:2010, "Surface active agents — Detergents — Determination of cationic-active matter content"

[3] J. Pernak, J. Feder-Kubis, *Chem. Eur. J.* **2005**, *11*, 4441–4449.

[4] P. Li, N. Unglaube, H. Zhou, S. Michel, X. Dong, X. Xu, A. Birnbaum, G. K. Auernhammer, Y. Xia, J. Grothe, S. Kaskel, *Chem. Eng. J.* **2023**, *477*, 146898.

[5] M. Mantina, A. C. Chamberlin, R. Valero, C. J. Cramer, D. G. Truhlar, *J. Phys. Chem. A* **2009**, *113*, 5806–5812.

Author Contributions

S.K. and J.G. directed the project. P.L. designed and carried out the electrochemical measurement, material characterization, *in situ*-Raman test, and wrote the paper. J.F.-K and M.Z.-B. synthesized and analyzed the ammonium-based ionic compounds. J.F.-K authored the section on the synthesis and the biological context of bioactive compounds and revised the manuscript. J.K. and P.L carried out MAS NMR experiments. E.B and J. K. finished the analyses of MAS NMR in the manuscript.
